# Safe(r)-by-design principles in the thermoplastics industry: guidance on release assessment during manufacture of nano-enabled products

**DOI:** 10.3389/fpubh.2024.1398104

**Published:** 2024-07-05

**Authors:** Polly McLean, James Hanlon, Apostolos Salmatonidis, Karen S. Galea, Finlay Brooker, Cristiano Citterio, Daniele Magni, Socorro Vázquez-Campos, Davide Lotti, Matthew S. P. Boyles

**Affiliations:** ^1^Institute of Occupational Medicine, Edinburgh, United Kingdom; ^2^Ricardo, Glasgow, United Kingdom; ^3^LEITAT Technological Center, Terrassa, Spain; ^4^LATI Industria Termoplastici S.p.A., Vedano Olona, Italy; ^5^Centre for Biomedicine and Global Health, School of Applied Sciences, Edinburgh Napier University, Edinburgh, United Kingdom

**Keywords:** safe(r)-by-design (SbD), nano-enabled products (NEPs), nanomaterials, nanoparticles, nano-objects, exposure assessment, 3D printing, additive manufacturing

## Abstract

**Background:**

The application of nanomaterials (NMs) and nano-enabled products (NEPs) across many industries has been extensive and is still expanding decades after first being identified as an emerging technology. Additive manufacturing has been greatly impacted and has seen the benefits of integrating NMs within products. With the expansion of nanotechnology, there has been a need to develop more adaptive and responsive methods to ascertain risks and ensure technology is developed safely. The Safe(r)-by-Design (SbD) concept can be used to establish safe parameters and minimise risks during the materials’ lifecycle, including the early stages of the supply chain. Exposure monitoring has advanced in recent years with the creation of standardised protocols for occupational exposure assessment of nano-objects and their aggregates and agglomerates (NOAA).

**Methods:**

To aid in the development of an online SbD-supporting platform by the EU-funded project SAbyNA, we adopt a Europe Standard for monitoring release of NOAA to identify if a greater release of NOAA is associated with incorporation of NMs within NEPs compared to a polymer matrix alone. Case studies included filaments of polypropylene (PP) with nano-Ag or polycarbonate (PC) with single-walled carbon nanotubes (SWCNTs). NMs were received in masterbatch, and therefore previously modified to align with SbD interventions. Results were collected in line with European Standard recommendations: monitoring particle concentrations using direct reading instruments (DRI), sampling for offline chemical and morphological analysis, and collecting contextual information.

**Results and discussion:**

Based on the criteria described in the European standard (BS EN 17058), data from both case studies identified that inhalation exposure relating to NM was “unlikely”. Despite this, during the production of the SWCNT-PC filaments, some noteworthy observations were made, including several DRI activity measurements shown to be higher than background levels, and material morphologically similar to the reference SWCNT/polymeric masterbatch observed in offline analysis. The data collected during this campaign were used to discuss choices available for data interpretation and decision-making in the European Standard for monitoring release of NOAA and also to facilitate the development of SAbyNA’s user-friendly industry platform for the SbD of NMs and NEPs.

## Introduction

The risks associated with nanomaterials (NMs) have been known for some time now, and although there have been and continue to be great advances to support risk assessment (RA) of NMs, processes are still hindered by uncertainties in both exposure and hazard assessment methods and how to fully utilise the two in combination (1–3). Moreover, with continued and expanding development of nanotechnology, there is an increasing use of nano-enabled products (NEPs). These include materials such as carbon nanotubes (CNTs), graphene, graphene oxide, silica, silver (Ag), titanium dioxide, and zinc oxide, which are commonly reported NMs used to enhance products in 3D printing (additive manufacturing) (4). With this expansion, there is a rising concern about how we will be able to adequately assess the risks posed by exposure to these materials, a concern that may, in part, be diminished with the application of safe(r)-by-design (SbD) principles. SbD strategies have received much attention in recent years. This is partially due to the inability of regulatory processes to provide accurate and timely assessment of the diverse and fast-paced innovation often seen in nanotechnology industries (5). The integrated use of SbD can assist in early-stage innovation and/or early-stage product processing, offering timely interventions that identify factors of risk and allow establishment of safe working conditions. Risks are minimised at all stages of the material lifecycle, including the early stages of the supply and value chain (6, 7). Although SbD may be applied to any stage of product development and should consider both hazards and exposure, if time is invested in the early stages of innovation, the implementation of intervention strategies may lead to cost-effective and timely mitigation of risks.

An important input parameter for SbD process is the measurement of potential exposure, and here empirical data are important. For effective use of empirical data, there is a need for harmonised approaches to exposure monitoring, and suitable methodology should be available to ensure that information can be collected and correlated to allow good decision-making. In recent years, there have been advances in exposure assessment of NMs with the creation of standardised protocols for occupational exposure assessment of nano-objects and their aggregates and agglomerates (NOAA). Within Europe, this is largely covered by the standards prepared by the European Committee for Standardisation (CEN) and the British Standards Institution (BSI) (8–11), whereas in the USA, protocols have been outlined by the National Institute for Occupational Safety and Health (NIOSH) (12). Supporting approaches are also provided by the Organisation for Economic Co-operation and Development (OECD) (13). In general terms, the aim of measuring occupational exposure could be for a number of outcomes: to determine how effective control measures are, to show compliance with occupational exposure limits (OELs), to generate data to support risk assessment (RA) (11), or to include in chemical safety reports (CSRs) under REACH. As there are currently no European OELs for NMs, the focus for occupational exposure monitoring is for use in RA. Exposure data for NMs can still be used to contribute to control banding exercises even in the absence of exposure limits (14) or to compare to Nano Reference Values (NRVs)—monitoring standards developed by the German Institute for Occupational Safety and Health (IFA) and Dutch Social Economic Council for use in the absence of OELs (15), and even without OELs, use of exposure data for RA can be strengthened by the application of a probabilistic approach; exposure data can be used either in parallel with dose–response assessment information and risk characterisation criteria—defining uncertainty and variability, providing an understanding as to what has influenced the final risk estimate (16), or used independently in probabilistic exposure modelling, to determine worst-case scenarios when comparing different industrial processes (17). This approach has been evidenced in the assessment of TiO_2_ (16), organic pigments applied in the automotive industry (18), and in the assessment of paint manufacturing processes (17). For certain materials, however, recommended exposure limits (RELs) values have been set that can be used until such time as OELs are set in regulation, including for CNT, Ag, and TiO_2_ (19–21).

In the assessment of exposure to NOAA, it is expected that the assessment is done in comparison to background levels; these can either be taken at a different location or a different time in relation to the NM handling or processing. Practically, the current BSI Standard BS EN 17058 (11) suggests three levels (or tiers) of assessment: initial, basic, and comprehensive. An *initial assessment* (Tier 1) is, in general, an information-gathering exercise with no monitoring performed, and the outputs used suggest risk management measures (RMMs), implemented in line with precautionary approaches, such as those suggested in control banding and risk management tools [e.g., Nanosafer (22), Stoffenmanager Nano (23), CB Nanotool (24)]. The *basic assessment* (Tier 2) will use measurement instruments, but these should be portable, require suitably qualified persons to undertake assessment, and include collection for offline analysis and time-resolved readings. Decision rules would be implemented in this tier (11), comparing direct reading instrument (DRI) data to either background concentrations or NRV (25–27), and then combined with the results of offline analysis to justify no evidence for exposure; this approach has many similarities to the NIOSH Nanoparticle Emission Assessment Technique (NEAT) 2.0 assessment (12, 28). The use of simple, portable DRIs allows assessment of how well RMMs (such as LEVs) are working and can identify high-risk processes specific to the site of interest. This is something the *initial assessment* is not able to do. The *comprehensive assessment* (Tier 3) is less cost-effective compared to Tier 2 and therefore less amenable for early innovation SbD but aims to characterise worker exposure and therefore has an emphasis on personal breathing zone (PBZ) sampling alongside the similar three-pronged approach of (1) monitoring of particle concentration using DRIs, (2) sampling for offline chemical analysis, and (3) collection of contextual information. As the instruments involved in Tier 3 are more sophisticated, they typically tend to be less manoeuvrable and cannot simply be attached to workers due to size, weight, and their requirement to be plugged into main power. Due to the added complexity of the equipment used and the subsequent data analysis, a greater level of expertise is required and hence results in elevated costs, meaning this is often impractical for companies to use within the development stages of designing processes involving NMs.

In a Horizon 2020 funded project—simple, robust, and cost-effective approaches to guide industry in the development of safer nanomaterials and nano-enabled products (SAbyNA)—SbD is being supported with the development of a user-friendly industry platform for the SbD of NMs and NEPs. The platform development uses case studies in the additive manufacturing and paint industries to test and apply strategies for SbD. In this article, we present the occupational exposure assessment, performed in line with BSI (11), for the additive manufacturing case studies. BSI highlights the use of this Standard to assess nano-objects and their NOAA; they acknowledge, however, that the approach may also be applied to measure particles released from nanocomposites, or NEPs; here we specifically address NEPs. The release of NOAA was assessed during the production of matrix containing NMs, either polypropylene (PP) with nano-Ag, polycarbonate (PC) with single-walled (SW)CNTs, or a matrix formed of either PP or PC alone; the use of these test materials allows for distinction of release specifically related to NM incorporation. Using the data collected, we outline how these assessment data are being used to support the development of SbD tools, and we discuss the options available for data collection and interpretation when using the Standard recognised in Europe for monitoring release and characterisation of NOAA, and how these impact on the decision rules used to confirm when particle exposure has occurred and is associated with the presence of NMs in NEPs.

## Materials and methods

### Nanomaterials

The NMs incorporated into polymer matrices included nano-Ag (agpure^®^ in a polypropylene masterbatch, 6,500 ppm Ag, discrete Ag particles have a spheroidal diameter of 15 nm) and SWCNT (provided in a dispersion by OCSiAl-Europe Sarl (Luxembourg), composed of polyol ester matrix 75% and SWCNT 25%, outer diameter 1.6 nm, fibre length > 5 μm, approximate aspect ratio 5,000).

### Process and workplace description

Workplace sampling was performed at a company that manufactures high-performance thermoplastic compounds, including filaments used for additive manufacturing. The filaments produced included nano-Ag with PP and other additives (which cannot be disclosed under propriety rights but do not include further ENMs) (Case Study 1) and SWCNTs with PC and other additives (also protected under propriety rights but also did not include further ENMs) (Case Study 2). SWCNT loading in the final compound is 0.4% w/w, while nano-Ag loading in the compound is 500 ppm (0.05% w/w). For small-scale production, the weighing of additives and filament manufacturing takes place in the pilot plant area (around 98 m^2^). The additives are weighed on precision scales and then poured manually inside the mixer by one operator. In the plant, the raw materials arrive at the mixer by different routes, but for small-scale production are added manually with a paddle by the operator. Windows/doors were open in the facility to provide natural ventilation. The industrial facility consists of five extruders, usually running simultaneously. There were five operators working in the extrusion department (one with each extruder) and a process manager. During the measuring survey, other extruders were in operation, but only one was used with ENMs. The plant was around 3,000 m^3^, with two floors. There were two large doors on each side of the plant that were opened during production. The operator worked continuously in close proximity to the extruder during the entire process. This included feeding the base polymer, other additives, and ENM (mixture obtained after weighing and mixing), sometimes manually, and checking that the extruded plastic flowed easily in and out of the cooling bath. The base polymer was fed automatically from the top feeder located on a mezzanine, although the process for feeding the feeder was performed manually where dust was generated. Additional feeders were in use for the mixture of base polymer, minor additives, and further non-nano-additives. There were multiple feeders in the extruder, some located on the second floor, around 5 m from the instrument inlets, and another one on the first floor, closer to the direct reading instruments. The extruders had local exhaust ventilation (LEV), and there was no general ventilation, although a few chimneys with forced venting on the roof top ensure some air circulation. Above the various areas of the extruder, there is a pipeline equipped with several arms, each of which collects the gaseous emissions generated during the compounding process; such aspirations are located close to the extruder’s mouths, which are the only openings from which particulate matter and chemicals generated by the thermal decomposition of the polymer and its additives would be extruded.

### Measurement strategy

Measurements were taken during weighing, mixing, cleaning, extrusion, and final filament production exposure scenarios, each using the thermoplastic compound alone (as a blank) or nano-enabled thermoplastic compound (Ag or SWCNT). For example, in the filament production of nano-Ag and PP, pellets produced via extrusion were fed into a Microex 3D filament (Eur.Ex.Ma) extrusion line to produce filaments suitable for 3D printing in a 98 m^2^ room. Windows/doors were open in the facility to provide natural ventilation. The temperature in the facility was 27 ± 2°C, and the humidity was 64 ± 2%. This process involved the manual filling of the hopper, stirring the hopper, adjusting the flow of the filament, and changing the spool. Heat was produced from the instrument during the melting of the pellets, and often the casing of the instrument would need to be lifted to make adjustments. Cleaning was performed nearby for around 14 min at the beginning of this exposure scenario. Background measurements included sampling at the same location before the activity, sampling during the activity when no nano-product was used, and sampling at a far-field (FF) location during the activity. Full details of each exposure scenario where measurements were taken are provided in the Supplementary material. Measurements followed the Tier 3 method outlined in BS EN 17058:2018 (11). Briefly, a suite of DRIs were used in combination with offline sampling of filters collected at the PBZ of the worker and at fixed locations in the near-field (NF) and FF. The DRIs chosen collected information on the particle number concentration (PNC), particle size distribution (PSD), and lung-deposited surface area. Throughout the measurement campaign, contextual information was collected. This can include any issues during the process, such as temperature and humidity, worker activity in the NF and FF, activities performed by workers for personal monitoring, and other activities being performed in the FF (such as other processes, equipment, or vehicles); all are time-stamped to enable correlation with the measurements taken.

### Direct reading instruments

The instruments used in the measurement campaign adhere to BSI (10) mass, surface area, or particle number; we included a range of instruments that provide either mass or number concentration. Real-time measurements were collected in the NF using five instruments, these being: Fast Mobility Particle Sizer (FMPS Model 3,091, 5–560 nm, 10.0 L/min, TSI Inc), Aerodynamic Particle Sizer Spectrometer (APS Model 3,321, 500 nm to 20 μm, 5.0 ± 0.2 L/min, TSI Inc), DustTrak DRX (Model 8,533, 100 nm to 15 μm, 3.0 L/min, TSI Inc.; provides size-segregated mass fraction concentrations corresponding to PM1, PM2.5, respirable, PM10 and PM Total size fractions, and can detect aerosol concentrations within a range of 0.001–150 mg/m^3^), Condensation Particle Counter (CPC Model 3,007, 5 nm to 1 μm, 0.7 L/min, TSI Inc) and Diffusion Size Classifier mini (DiSCmini V2.0, 10–700 nm, 1.0 ± 0.1 L/min, Testo AG). All instruments were set to collect concentrations with a time resolution of 1 s. Conductive silicon tubing (TSI Inc.) was attached to instruments, with inlets placed at BZ height on a stand directly above the hopper of the instrument for the duration of sampling to represent the “worst-case” personal exposure. In the FF location, a separate CPC was used to measure simultaneous background concentrations with time resolution of 60 s. Data generated by DRIs were processed using an “exposure assessment app” developed in-house by IOM; a combination of a Windows app and Excel macros was used. The Windows app is used to select the different data files, ensure that all the data are correctly matched up with time stamps, and combine them into an Excel template file. Macros within the Excel template then separate the data into the various time sections for separate analysis and to generate the tables and graphs needed.

PNCs were described using mean values, standard deviations, and maximum and minimum values. Differences between the mean PNC during the nano-activity and the background PNC were determined using (1) the sequential method (or “NF” approach), (2) measurement during the same activity when no NOAA is present, and (3) the simultaneous method (or “FF” approach). Any transient peaks in the time series were investigated further as required.

The decision rule followed to assess the significance of all DRI data is described in Eq. 1:

(1) Mact>Mbgr+3×SDbgr

where *M*act is the mean concentration of airborne particles during activity; *M*bgr is the mean background concentration of airborne particles (which can be applied according to any of the background determination methods identified above); *SD*bgr is the standard deviation of the background concentration.

### Offline analysis

PBZ, NF, and FF samples were collected on either 25 mm polyvinyl chloride (PVC) filters mounted in Higgins-Dewell plastic cyclones (Casella) or 25 mm PC filters mounted in plastic cowls (SKC Ltd.) to measure the respirable aerosol fraction. The cyclones and cowls were attached to a pump (SKC Ltd.) set to 2.2 L/min. Flow rates were checked before and after sampling using a digital flowmeter (Model 4,100, TSI Inc.).

Filter samples (PBZ and NF) were analysed by scanning electron microscopy (SEM) (Hitachi S-2600 N) and EDX (energy-dispersive X-ray spectroscopy) (Oxford Instruments X-Max), along with inductively coupled plasma atomic emission spectroscopy (ICP-AES) (Thermo Scientific iCAP Duo 6,500). Filter samples and reference materials were analysed by image and elemental profiling using a modification of an internal Standard Operating Procedure (SOP), which is adopted from ISO (2019). In preparation for SEM/EDXS analysis, a portion of each filter or tape sample was excised and mounted onto a 13-mm-diameter aluminium SEM stub using adhesive carbon tabs and coated (Edwards S150B sputter coater) with a thin layer of gold to enhance the conductivity of the surface and the imaging resolution. The filter is scanned at 2000× magnification. Fields are analysed for the presence of particles/fibres that have the potential to have originated from the process, i.e., SWCNT-based. Images were recorded at various magnifications to best represent the distribution, size, and shape of particles observed, and elemental analysis was carried out for chemical composition. ICP-AES analysis was for the presence of silver (for the nano-Ag/PP filaments) and iron (Fe) for the SWCNT-PC filament, as it has previously been identified that at 15% w/w, Fe is the major impurity in these SWCNTs. The methodology followed corresponded to the technique recommended by the Occupational Safety and Health Administration: OSHA ID-121: “Metal and Metalloid Particulates in Workplace Atmospheres (Atomic Absorption).” The reporting limit for metals by this method, and therefore relevant to our tracer elements, was 0.6 μg.

### Categorising likelihood of exposure induced by the nanomaterial activity

Particulate exposure was categorised as “likely”, “presumable”, “possible/not excluded”, or “unlikely” according to criteria outlined in Table 1 [adapted from BSI (11) and Bekker et al. (29)].

**Table 1:** Criteria for determining the probability of exposure through quantitative and qualitative measurements.

Offline analysis^a^—NM BG^b^	Offline analysis^a^—NM activity^c^	Online monitoring (DRI)^d^	Conclusion (likelihood of exposure induced by the nanomaterial activity)
−	+	Significant	Likely
+	+	Significant	Presumable
−	+	Non-significant	Possible/not excluded
+	+	Non-significant	
−	−	Non-significant	Unlikely
−	−	Significant	

(+) Indicates a positive confirmation of the specific NM being present, and (−) indicates the presence of the specific NM was not confirmed.

a SEM/EDX, ICP-OES.

b process conducted with matrix not containing nanomaterial.

c process conducted with matrix containing nanomaterial.

d level of exposure compared to background, significantly identified when the activity exceeded background level—concluded by decision rule (DR).

## Results

### Case study 1: nano-Ag/PP filaments

During the different stages of filament production, various exposure scenarios were monitored for particle release. These were weighing, mixing, cleaning, extrusion, and final filament production. For each of these stages, DRI and offline measurements were performed during the processing of filaments formed using masterbatch containing or lacking nano-Ag. The overview of results for Case Study 1—filament made from nano-Ag and PP—is shown in Supplementary Table S1, which includes measurement data of all instruments used and for all stages monitored, as well as provides cut-off values based on background values determined by the sequential method, and by sampling of the same activity with no nano-Ag present in the masterbatch. We have used Exposure Scenarios 8 and 9 (Filament production of PP filaments with and without nano-Ag) to discuss this in more detail, as this exposure scenario included a third background setting method, that of simultaneous FF measurements. Results from filament production using PP with nano-Ag are summarised in Table 2.

**Table 2:** Summary data of exposure scenario 9: filament production of nano-Ag and PP.

DRI		DRI results			Offline analysis results		Probability of exposure conclusion (likelihood of exposure induced by the nanomaterial activity)
Type	Size range detected	Average	Cut-off	Significance of result	NM BG	NM activity	
FMPS (#cm^3^)	5–560 nm	488,977	1575682^a^	X	X	X	Unlikely
			2863679^b^	X			
APS (#cm^3^)	500 nm–20 μm	1,548	1974^a^	X	X	X	Unlikely
			2953^b^	X			
CPC (#cm^3^)	5 nm–1 μm	29,712	87,799^a^	X	X	X	Unlikely
			150018^b^	X			
			51685^c^	X			
DustTrak (mg/m^3^)	100 nm–15 μm	0.048	0.087^a^	X	X	X	Unlikely
			0.107^b^	X			
DiSCmini (#cm^3^)	10–700 nm	17,686	51598^a^	X	X	X	Unlikely
			128306^b^	X			

Includes detection of particles present when measured with different DRIs, incorporating background controls and using NMs to provide a probability of exposure relating to NM presence in products; output of DRI is in a number of particles per cm^3^, except DustTrak, which is in particulate mass concentrations (mg/m^3^); total activity time: 27 min, 57 s; NM: nanomaterial, NM BG: samples collected when using masterbatch not containing NM; NM activity: samples collected when using masterbatch containing NM; X = a non-significant finding (i.e., the release detected by DRI during use of NM did not exceed cut-off values, or no evidence of the specific NM was observed in offline analysis).

a Using background value determined via the sequential method.

b Using background value determined via sampling of the same activity with no NOAA present.

c Using background value determined via the simultaneous method.

In general, it was found that the probability of exposure of particles above those of background was “unlikely”, and this was also true for other stages of production (Supplementary Table S1); in all stages of production of nano-Ag/PP filaments, the potential of exposure to released material was low, both when nano-Ag was included and when excluded, according to DRI, and ICP-AES filter samples were collected during the filament manufacturing; these samples were below the LOD for Ag. Cut-off values were generally higher when using the method, compared to the control using masterbatch not containing nano-Ag, and in the one example possible, the cut-off value was lowest using the simultaneous assessment.

**Figure 1:**
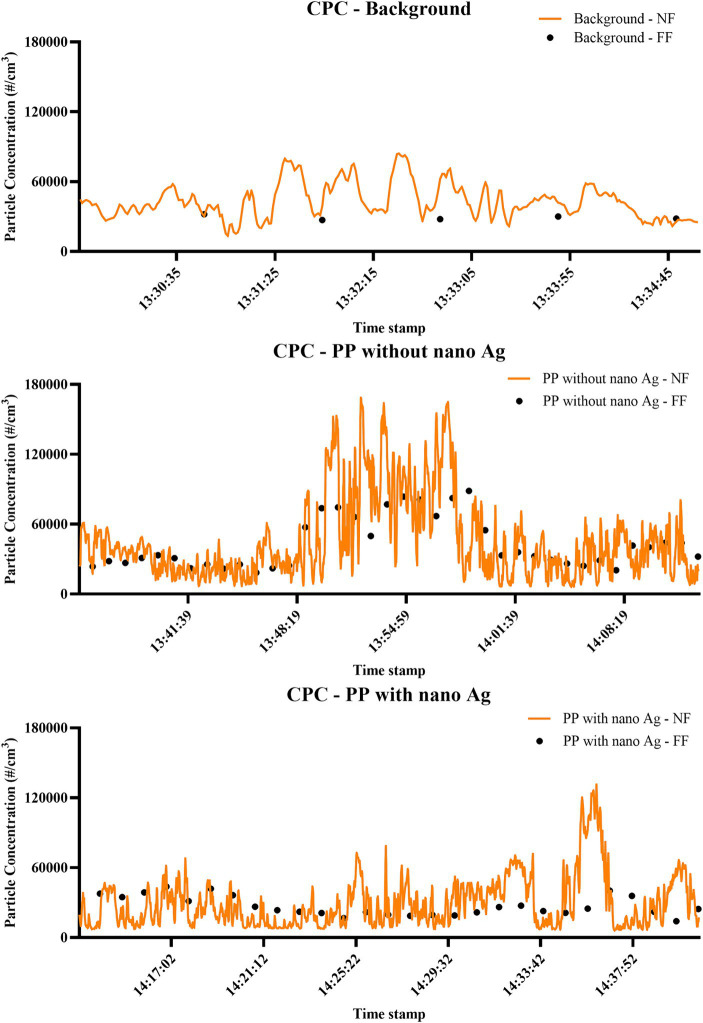
Time-relevant particle detection during PP filament production. Measurements performed by CPC; concentration is expressed as particle number distribution (#/cm^3^); conducted during background measurements; filament production using PP only; and filament production using nano-Ag with PP.

Data in Table 2 are averaged particles over time; however, measurements in each exposure scenario were also found to provide particle release peaks at various points, these were typically only for a matter of seconds, and as such, the average particle release over time was focused on for the analysis (all averaged data shown in Supplementary material). In our representative exposure scenario (filament production of PP filaments with and without nano-Ag), a number of peaks of a slightly longer period were observed, both in the processing of PP filament and PP with nano-Ag filament. In PP filament production without Ag, peaks reached up to approximately 120,000 particles/cm^3^ when measured by CPC (Figure 1), and up to 0.17 mg/m^3^, 4,900 particles/cm^3^, 3,500,000 particles/cm^3^, and 180,000 particles/cm^3^ for DustTrak, APS, FMPS, and DiSCmini, respectively (time-stamped data for all instrumentation— Supplementary Figure S1). In comparison to the contextual information identified, release recorded on CPC, FMPS, and DiSCmini was likely associated with activity at a nearby extrusion line unrelated to the activity being monitored, while measurements from DustTrak and APS were linked to bath lid opening and hopper stirring and emptying. In PP filament production with masterbatch containing nano-Ag, transient peaks of approximately 160,000 particles/cm^3^ were observed by CPC (Figure 1), and 2,500,000 particles/cm^3^, 120,000 particles/cm^3^, by FMPS, and DiSCmini, respectively (Figure 1), and up to 0.17 mg/m^3^, 4,900 particles/cm^3^, 3,500,000 particles/cm^3^, 180,000 particles/cm^3^ for DustTrak, APS, FMPS, and DiSCmini, respectively (time-stamped data for all instrumentation—Supplementary Figure S1); contextual information link these to bath lid opening and hopper stirring and emptying. For reference, background measurements (Supplementary Figure S1) would be consistently below 70,000 particles/cm^3^, 0.10 mg/m^3^, 1,500 particles/cm^3^, 1,600,000 particles/cm^3^, and 50,000 particles/cm^3^ for CPC, DustTrak, APS, FMPS, and DiSCmini, respectively.

**Figure 2:**
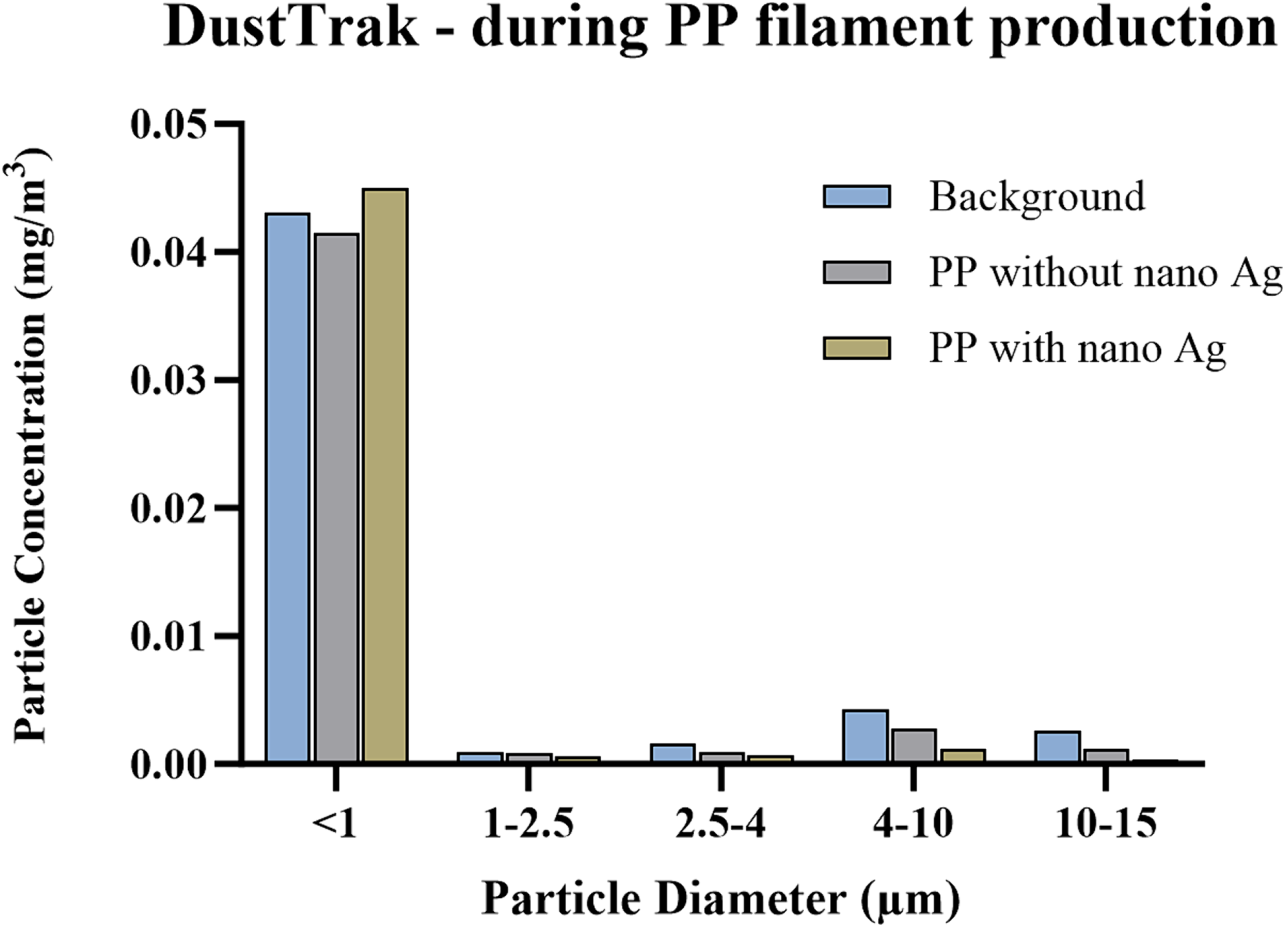
Size-segregated particle mass concentrations during PP filament production, measured by DustTrak. Concentration is expressed as particle mass concentration (mg/m^3^); particle size bins include <1 μm, 1–2.5 μm, 2.5–4 μm, 4–10 μm, 10–15 μm; conducted during background measurements; filament production using PP only; and filament production using nano-Ag with PP.

**Figure 3:**
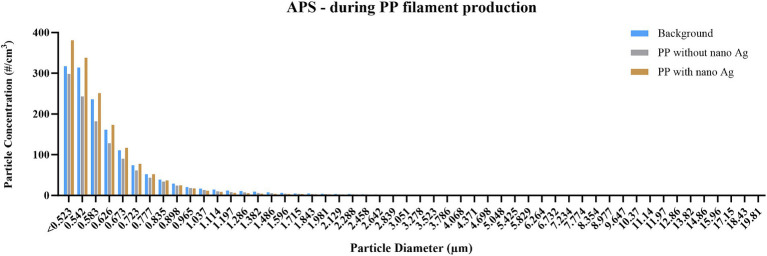
Particle size distribution during PP filament production, measured by APS. Concentration is expressed as particle number distribution (#/cm^3^); particles are detected between 0.5 and 20 μm, using 52 size channels, and 1 s resolution; measurements conducted during background measurements; filament production using PP only; and filament production using nano-Ag with PP.

**Figure 4:**
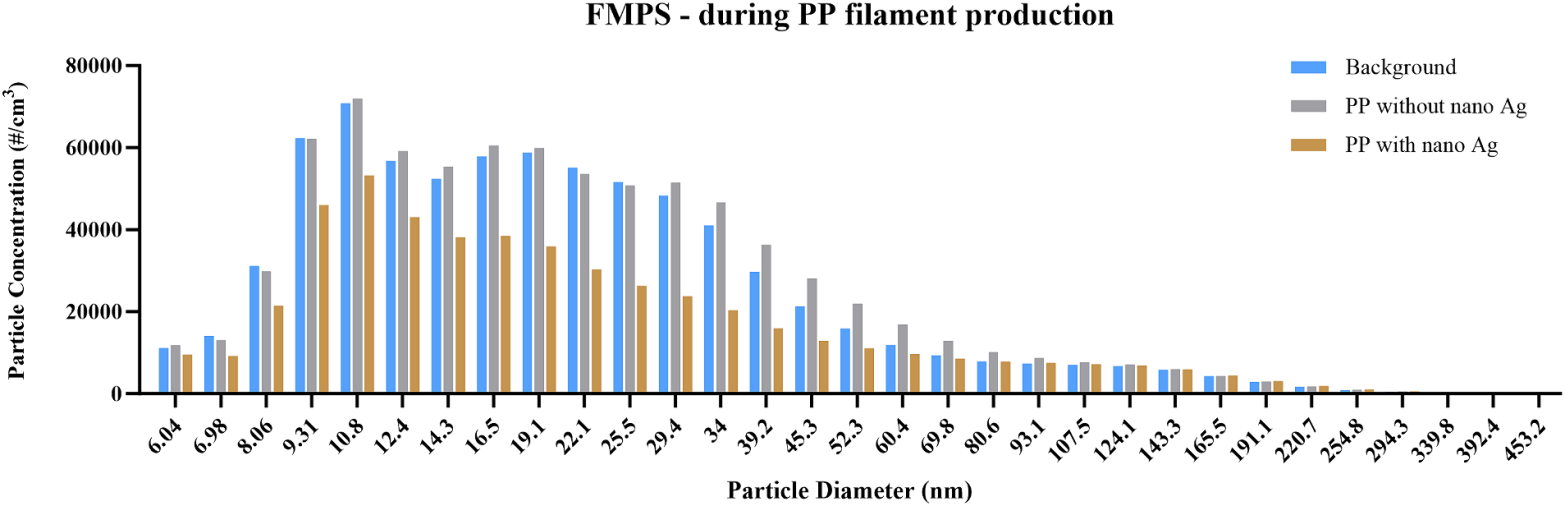
Particle size distribution during PP filament production, measured by FMPS. Concentration is expressed as particle number (#/cm^3^); particles detected between 5 and 560 nm, using 32 size channels, and 1 s resolution; measurements conducted during background measurements; filament production using PP only; and filament production using nano-Ag with PP.

For instruments that provide PSD, or size-segregated particle mass concentrations, released particles were analysed accordingly. DustTrak measurements (Figure 2) identified that 82, 89, and 94% of particles detected were below 1 μm in measurements of background, PP, and PP + nano-Ag, respectively, and that release within this size fraction was between 42 and 45 μg/m^3^, in all three cases. In assessment by APS (Figure 3), particles were confirmed to be within this same size range. Most detected were below 1 μm, with the number of particles detected increasing as the size fraction was reduced; the highest proportion of particles released were within the <523 nm fraction, with 317, 298, and 381 particles per cm^3^ for background, PP, and PP + nano-Ag, respectively. In FMPS analysis (Figure 4), the PSD followed a Gaussian probability distribution, with the highest proportion of particles in each scenario being approximately 10 nm. Although the production of filament with PP and nano-Ag appeared to have a slightly lower release of particles at this smaller size fraction.

### Case study 2: SWCNT-PC filaments

PC filament production was monitored for particle release during weighing, mixing, cleaning, extrusion, and final filament production and assessed by DRI and offline analysis, including ICP-AES and SEM with EDX. Filament production was performed using masterbatch which either contained or was absent of SWCNTs. The overview of results for Case Study 2—filament made of SWCNT with PC, is in Supplementary Table S2, including averaged, maximum, and minimum readings from each activity and all DRI, with cut-off values, relating to the previously described backgrounds used in decision logic, are based on the measurements performed before and after processes or on measurements conducted during processing of the masterbatch which contained no NM. As previously observed for the production of nano-Ag/PP filaments, the probability of exposure to particles containing SWCNTs was concluded “unlikely” according to the decision logic. Again, we have focused on Exposure Scenarios 8 and 9 (filament production of PC filaments with and without SWCNTs); these included simultaneous FF measurements, which allowed us to compare all three methods for setting cut-off values. However, we also highlight certain observations from other exposure scenarios. These observations did not affect the final conclusion of “unlikely” release associated with the addition of SWCNT to the masterbatch but were still considered noteworthy. These include DRI measurements above background levels in weighing, mixing, and extrusion and notable SEM observations in the extrusion process. Due to transient release peaks generally lasting only a few seconds (Supplementary Figure S1), average particle release over time was again used for the general analysis. In Table 3, it can be seen that average particle measurements during filament production of PC filaments containing SWCNT were consistently below each of the defined cut-off values, and no significant, as per decision logic, observations were made in offline analysis, resulting in it being “unlikely” that exposure would occur relating to the addition of SWCNT to filaments. Again, cut-off values were higher when using the method compared to the control using masterbatch not containing SWCNT, and the cut-off value that required simultaneous assessment provided the lowest value. As also noted in Case Study 1, the background PNC levels were particularly high for this activity due to high humidity and high energy processes occurring in the FF.

**Table 3:** Summary data of exposure scenario 9: filament production of SWCNT and PC.

DRI		DRI results			Offline analysis results		Conclusion (likelihood of exposure induced by the nanomaterial activity)
Type	Size range detected	Average	Cut-off	Significance of result	NM BG	NM activity	
FMPS (#cm^3^)	5–560 nm	1,051,122	1575682^a^	X	X	X	Unlikely
			6734336^b^	X			
APS (#cm^3^)	500 nm–20 μm	NA	1974^a^	NA	NA	NA	Unlikely
			NA	NA			
CPC (#cm^3^)	5 nm–1 μm	44,155	87,799 ^a^	X	X	X	Unlikely
			159367^b^	X			
			51685^c^	X			
DustTrak (mg/m^3^)	100 nm–15 μm	0.025	0.087^a^	X	X	X	Unlikely
			0.061^b^	X			
DiSCmini (#cm^3^)	10–700 nm	37,873	51598^a^	X	X	X	Unlikely
			456583^b^	X			

Includes detection of particles present when measured with different direct reading instruments (DRI), incorporating background controls and using nanomaterials to provide a probability of exposure relating to NM presence in products; output of DRI is in a number of particles per cm^3^, except DustTrak, which is in mg/m^3^; total activity time: 37 min, 59 s; NM: nanomaterial, NM BG: samples collected when using masterbatch not containing NM; NM activity: samples collected when using masterbatch containing NM; X = a non-significant finding; due to instrument failure, not all comparisons were possible for APS analysis.

a Using background value determined via the sequential method.

b Using background value determined via sampling of the same activity with no NOAA present.

c Using background value determined via the simultaneous method.

**Figure 5:**
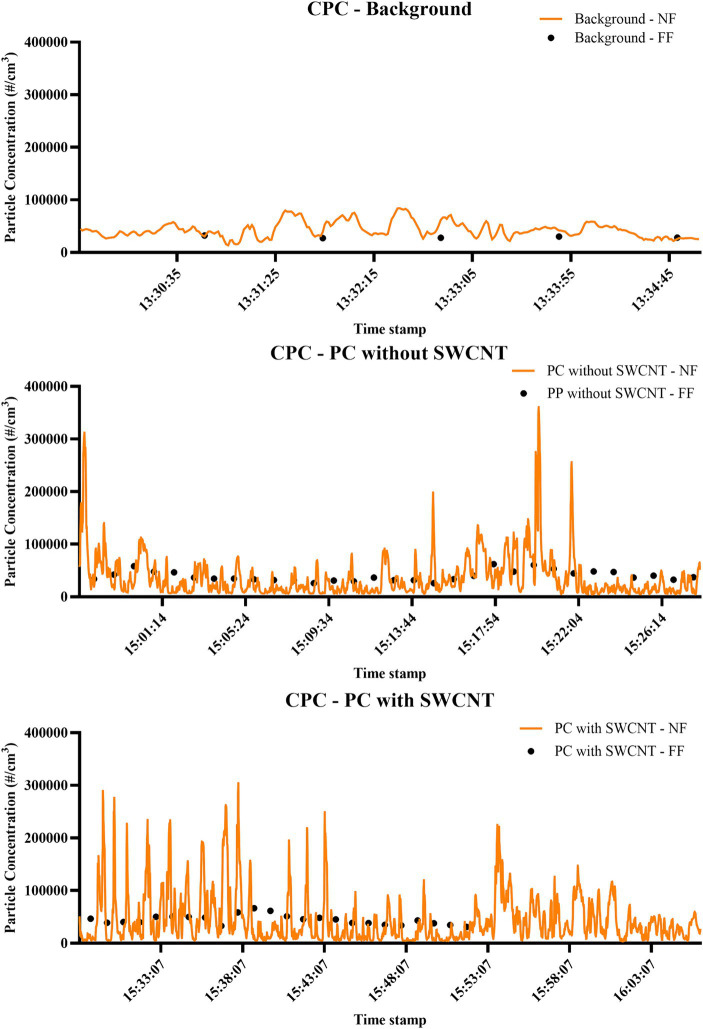
Time-relevant particle detection during PC filament production. Measurements performed by CPC; concentration is expressed as particle number distribution (#/cm^3^); conducted during background measurements; filament production using PC only; and filament production using SWCNT with PC.

The data in Table 3 are representative of averaged release values over time; however, in our representative exposure scenario (filament production of PC filaments with and without SWCNT), peaks were still observed in both processing situations when SWCNT were present or absent, as shown in temporal data from the NF CPC (Figure 5) and other DRI in Supplementary material (Supplementary Figure S1). In PC filament production without SWCNT, intermittent spikes of particle release were measured at up to approximately 250,000 particles/cm^3^ when measured by CPC, and up to 3,500,000 particles/cm^3^ and 2,500,000 particles/cm^3^ were observed by FMPS and DiSCmini, respectively. APS measurements showed no such spikes but were slightly raised during the first few minutes, dropping from approximately 700 particles/cm^3^ to 300 particles/cm^3^. Contextual information linked the initial release recorded by the APS to the sample being added to the hopper and measurements in CPC, DiSCmini, and FMPS to PC being added to the hopper, the spool being changed, and the bath being opened and stirred. In PC filament production with SWCNT, very sporadic spikes were, again, observed and measured at up to approximately 300,000 particles/cm^3^ by the CPC and up to 25,000,000 particles/cm^3^ and 900,000 particles/cm^3^ by the FMPS and DiSCmini, respectively. Contextual information linked these to a number of impromptu activities with filament production, including spool adjustment, spool removal, and powder being added. For comparison, the same background data given before demonstrated consistent measurements of below 70,000 particles/cm^3^, 0.10 mg/m^3^, 1,500 particles/cm^3^, 1,600,000 particles/cm^3^, and 50,000 particles/cm^3^ for CPC, DustTrak, APS, FMPS, and DiSCmini, respectively.

**Figure 6:**
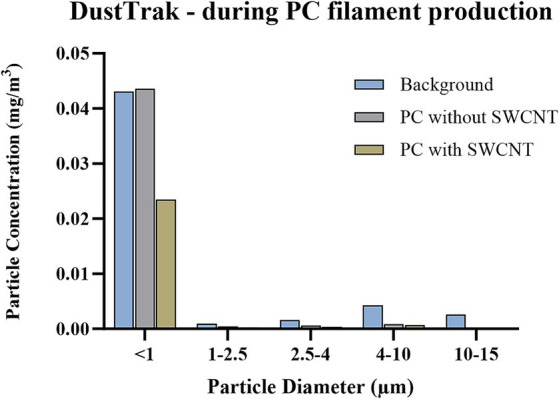
Size-segregated particle mass concentrations during PC filament production, measured by DustTrak. Concentration is expressed as particle mass concentration (mg/m^3^); particle size bins include <1 μm, 1–2.5 μm, 2.5–4 μm, 4–10 μm, 10–15 μm; conducted during background measurements; filament production using PC only; and filament production using SWCNT with PC.

**Figure 7:**
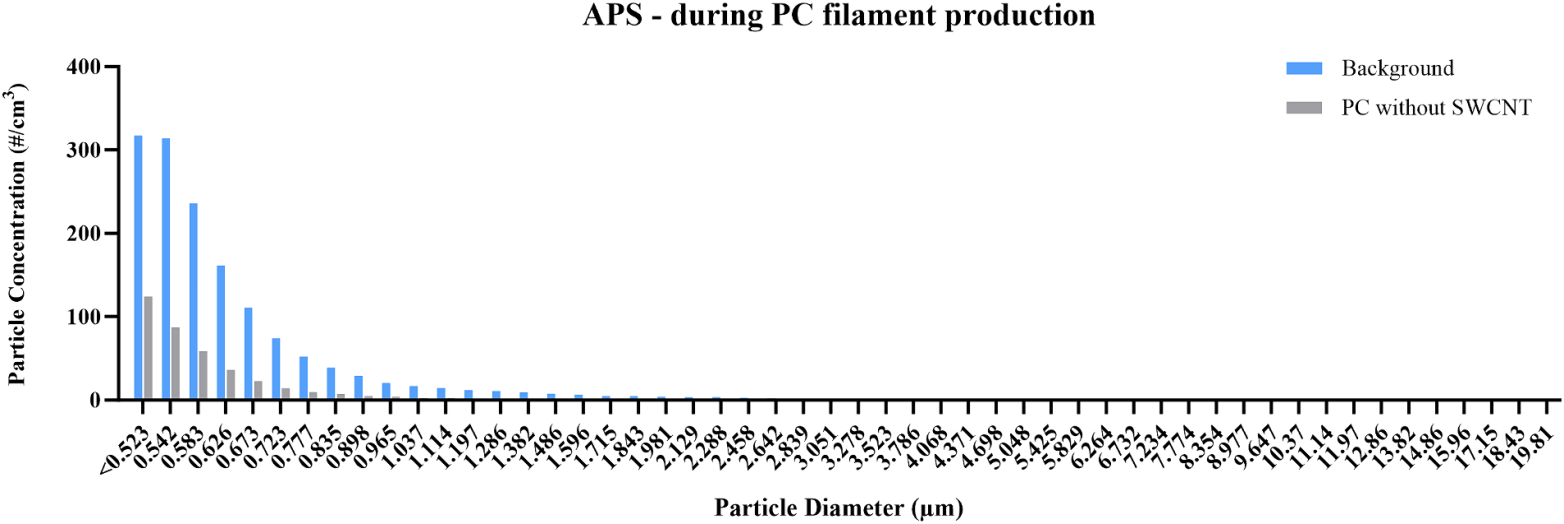
Particle size distribution during PC filament production, measured by APS. Concentration is expressed as particle number distribution (#/cm^3^); particles detected between 0.5 and 20 μm, using 52 size channels, and 1 s resolution; measurements conducted during background measurements; and filament production using PC only. Due to instrument failure, no measurements were obtained for filament production using SWCNT with PC.

**Figure 8:**
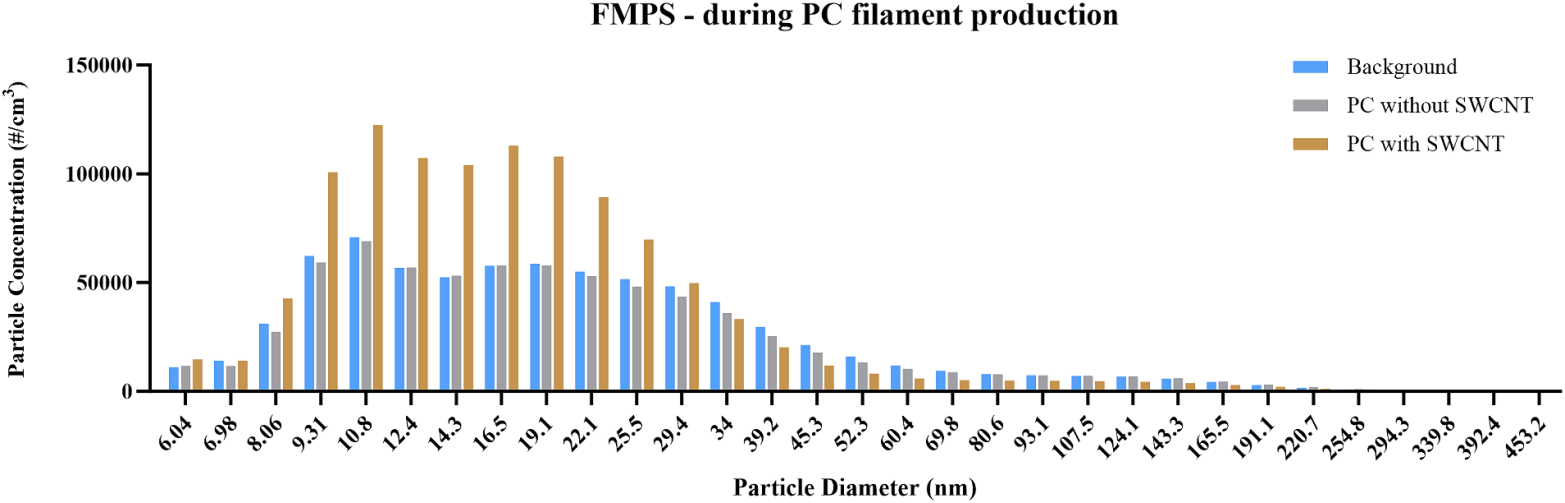
Particle size distribution during PC filament production, measured by FMPS. Concentration is expressed as particle number (#/cm^3^); particles detected between 5 and 560 nm, using 32 size channels, and 1 s resolution; measurements conducted during background measurements; filament production using PC only; and filament production using SWCNT with PC.

In the mass-based assessment by DustTrak (Figure 6), over 80% of material detected during background reading was shown to be <1 μm, while over 90% of material was <1 μm when processes of either filament type were performed. However, the mass detected in this size range was reduced from 43.1 μg/m^3^ to 39.1 μg/m^3^ to 23.5 μg/m^3^ when measuring background, PC only, and PC with SWCNT, respectively. Due to instrument failure, measurement by APS was only achieved for background and PC; this confirmed the <1 μm size fraction, and also that the greatest number of particles detected was during background measurements (Figure 7). Of those particles <500 nm detected by FMPS (Figure 8), a Gaussian probability distribution was again observed, with particles being predominantly 10–20 nm in diameter. In this size fraction, however, a greater number of particles were observed for filament production of PC with SWCNT, while the background and PC only were shown to be similar.

**Figure 9:**
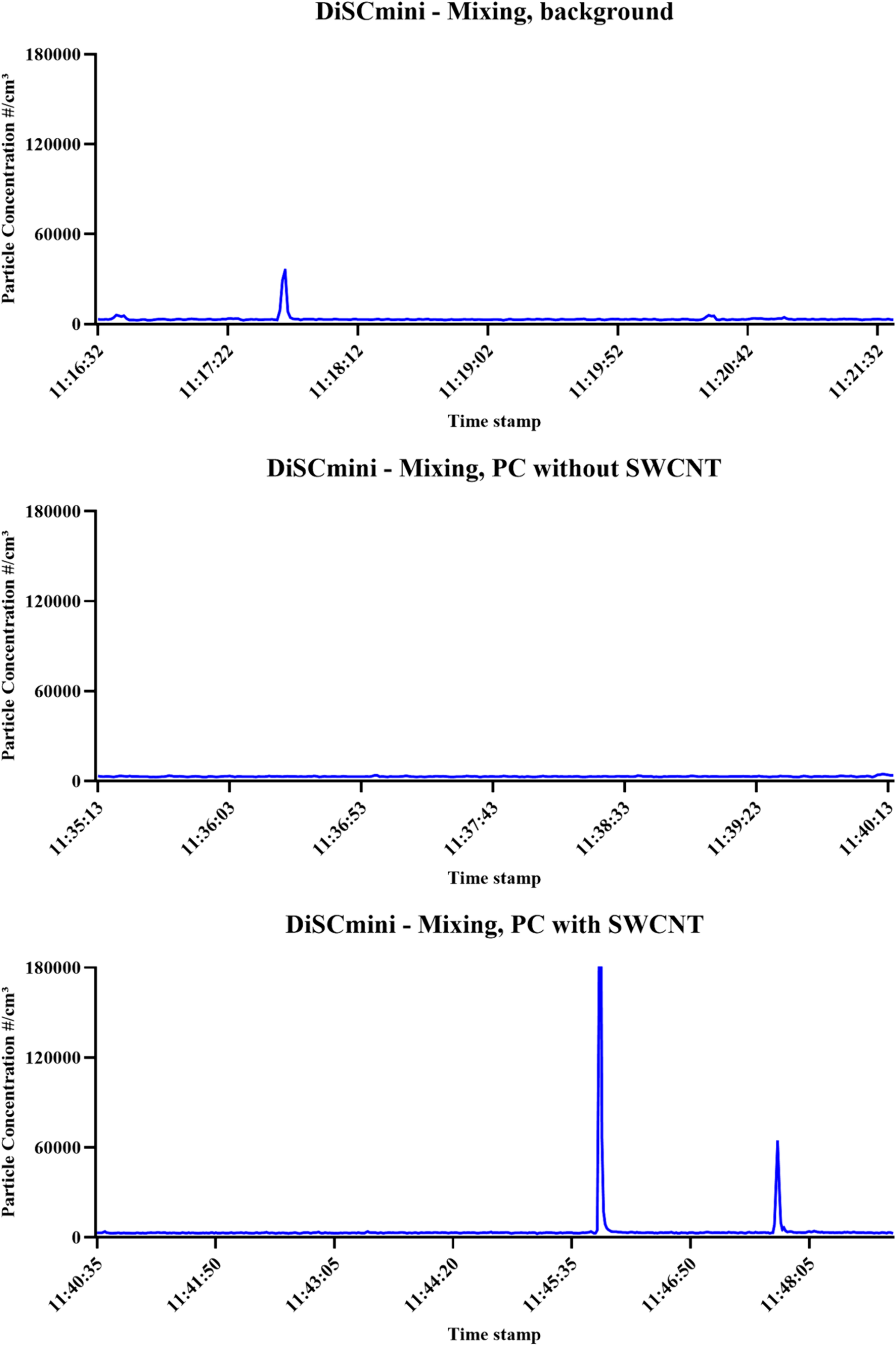
Time-relevant particle detection during PC filament production. Measurements performed by DiSCmini; concentration is expressed as particle number (#/cm^3^); conducted during background measurements; filament production using PC only; and filament production using SWCNT with PC.

As can be observed in Supplementary Table S2 and Supplementary Figures S2–S4, on several occasions the averaged particle release value for an activity was shown to be greater than one of the cut-off values; these were observed in weighing, mixing, extrusion, and cleaning after extrusion. These, however, were inconsistent across measurement devices. Most of these observations occurred in comparison to the sequential method of background setting and included measurements by CPC and DustTrak in weighing of PC (not observed by FMPS, APS, or DiSCmini), by CPC in weighing of PC + SWCNT (not observed in FMPS, APS, DustTrak, or DiSCmini), by CPC, DustTrak, and DiSCmini in extrusion of PC (not observed by FMPS or APS), by CPC and DiSCmini in extrusion of PC + SWCNT (not observed in FMPS, APS, or DustTrak), and by CPC when cleaning post-extrusion (not observed in FMPS, APS, DustTrak, or DiSCmini). For measurements taken by CPC and DustTrak during extrusion, contextual information identified no other impact on the process other than the task being undertaken. In one instance, the averaged particle release was found to be greater than the cut-off value set according to the background associated with the process being conducted without NM. This was observed for DiSCmini measurements during the mixing of PC + SWCNT (Figure 9), while not in CPC, FMPS, APS, or DustTrak. The peak observed from 11:45:52 to 11:45:55 during the mixing of PC + SWCNT was up to approximately 300,000 particles/cm^3^ and not linked to any clear events in contextual information, while the second peak of approximately 50,000 particles/cm^3^ was linked to the mixture being bagged. The spike observed in background measurements of up to 36,000 particles/cm^3^ was linked to a worker nearby conducting unrelated activities. Offline analysis (SEM with EDX, and ICP-AES) provided no evidence of particle release associated with SWCNT-containing matrix.

**Figure 10:**
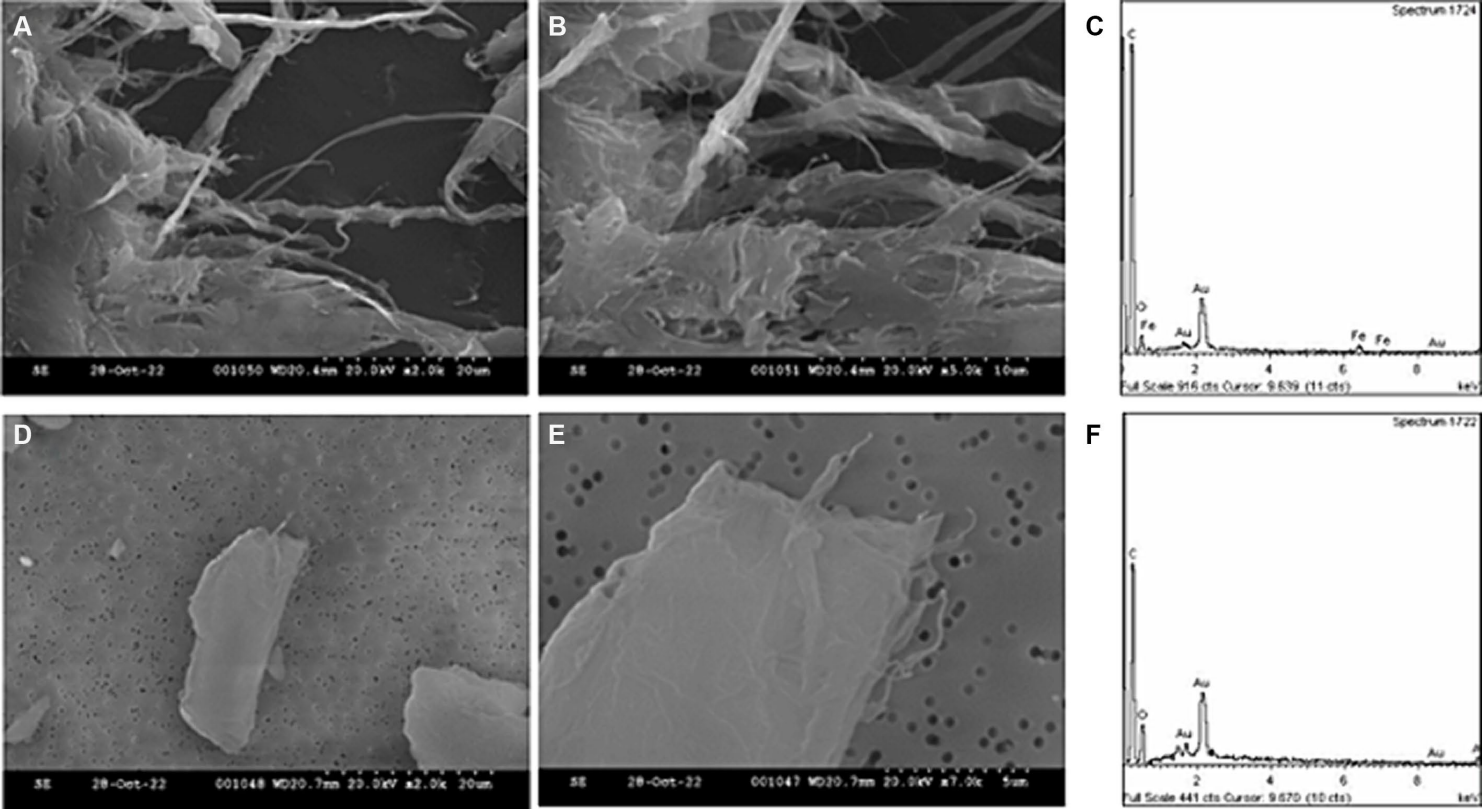
SEM and EDX analysis of SWCNT reference material, and PC filament production. SEM images include SWCNT/polymeric matrix masterbatch **(A,B)** with accompanying EDX spectra **(C)**, and particles deposited on filter during personal sampling **(D,E)**, with accompanying EDX spectra **(F)**.

During the extrusion of the SWCNT-PC filaments, collection and subsequent SEM analysis of personal filter samples showed the presence of large particles that exhibited morphology that was similar to that of the SWCNT/polymeric matrix masterbatch; note that the masterbatch is not individual CNTs in line with existing SbD considerations, the masterbatch is formed of SWCNTs embedded in polymer matrix. A range of particle sizes and morphologies were observed when assessing each stage of filament production, extrusion, and production by SEM, and all were found to be carbon-based (Supplementary Tables S4, S5). With personal sampling during the extrusion process, objects were identified by SEM that were likened to the morphology of the SWCNT feed material (commercially available SWCNT dispersed and embedded within a polymeric matrix), being large (tens of microns), flat, and with fibrous strands of a few hundred nanometres outlining the edges (Figure 10). Note that in either case, these were not observed as single SWCNT fibres, and no free SWCNTs were observed. Elemental analysis by EDX demonstrated a small Fe peak in the SWCNT/polymeric masterbatch reference material (Fe being used as a tracer for the SWCNTs), while no peak was observed for the objects collected during personal sampling of the extrusion process. Confirmation of SWCNT presence would require further analysis by, for example, transmission electron microscopy (TEM), which would also be a technique better suited for analysis of the smaller size fractions observed in FMPS measurement data.

## Discussion

In this measurement campaign, the release of NOAA was monitored using two case studies relating to the high-tech thermoplastics industry, specifically additive manufacturing. All stages of filament production of a matrix were assessed, with filament produced from either PP alone, PP with nano-Ag, PC alone, or PC with SWCNTs. With these differences in composition, we were provided with an opportunity to quantitatively identify when the inclusion of an NM and therefore the production of an NEP may result in extraneous release of particles compared to either background measurements or measurements of the same activity without the nano-additive. Here, we discuss this measurement campaign, in general, and comment on the likelihood of exposure based on our findings, we describe how these data are helping to facilitate the development of new resources that enable industry to apply SbD strategies and discuss the data in the context of using different measurement methods (i.e., instrumentation), using specific protocols relating to the tiered testing approach, and using different data analysis strategies to set background levels.

### Measurement campaign

In each filament production, nano-Ag/PP filaments and SWCNT-PC filaments, the potential of exposure to either nano-Ag or SWCNT was “unlikely”, particularly when compared to real-time measurements of either PP or PC alone. Hence, it is mostly related to process-generated (nano)particles rather than engineered NMs. ICP-AES filter samples were collected during the filament manufacturing, and these samples were < LOD for Ag and for Fe (marker for SWCNT presence) in all collected filter samples; the LOD could be improved by using other techniques such as inductively coupled plasma mass spectrometry (ICP-MS). During SEM analysis of a personal filter sample collected during extrusion of the SWCNT-PC filaments, one large particulate was identified which exhibited a morphology that was similar to that of the reference SWCNT/polymeric matrix masterbatch. The masterbatch consists of SWCNT dispersed and embedded within a polymeric matrix, and when observed under SEM, it has fibrous protrusions along its edge. These protrusions, however, are not necessarily CNTs. The detected release during the extrusion process had these similar features. However, while the masterbatch displayed evidence of Fe, the tracer metal for SWCNT presence, through EDX analysis, the material detected during the extrusion sampling did not. During PC filament production, on a number of occasions, a particle release was shown to be greater than background values (according to specific cut-off values). When setting the cut-off to sequential background measurements, increases were shown both when SWCNT were present in the masterbatch and when absent from the masterbatch. However, this was not consistently shown across all DRI instruments, and neither were they linked to offline analysis. During weighing of PC alone and PC with SWCNT, particle release was also measured; CPC analysis demonstrated an increase compared to background levels determined by sequential background. The peaks observed were similar in particle number and, therefore, considered a process-related event and not specifically related to the presence of SWCNTs. The one event evident, which provided a particle release that was greater when processing material that contained NM compared to the cut-off value calculated from the process performed without NM—during DiSCmini personal monitoring of the mixing activity—was not confirmed by offline analysis. Therefore, overall, release was categorised as “unlikely”.

For both case studies described, the background PNC levels were particularly high. As described earlier, it is believed this is due to high humidity and other high-energy processes occurring in the FF. Background levels as high as those observed in our examples are likely to contribute to uncertainty with regard to the overall conclusions. Furthermore, the concentrations of NMs present in the NEPs we describe are very low (chosen as an SbD option, thereby only using the lowest amount required to provide optimal properties of the material produced) and may require particularly sensitive methods of analysis in order to detect any release. A further hurdle in campaigns to measure exposure to NMs is that often processes are not performed in isolation, and this was true for both our case studies. It is also not always feasible to conduct repeat measurements of the activities due to constraints both with regards to the process (here we examined on a pilot scale, therefore there is not enough material to perform testing multiple times) and to the facility (costs incurred, and site activities are disrupted to allow measurement campaigns to be performed). This is a predicament discussed by others conducting similar assessments (30). Should repeat measurements be possible, conclusions from these measurement campaigns would be more robust, allowing better understanding of transient peaks and would provide reproducibility in baseline measurements.

Although the category for exposure probability was determined to be “unlikely” for material-related NMs, we did observe peaks due to process-related release. Therefore, recommendations can be made to improve the process. As the activities measured were on a pilot scale, these recommendations are 2-fold: initial recommendations to implement on the pilot scale and subsequent considerations when scaling up manufacturing. Suggestions made for initial improvements considered the hierarchy of control and included enhancing local exhaust ventilation (LEV) systems (i.e., shape of hood, position, and exhaust air velocity) to enclose the activity as much as possible, and where improvements to LEV systems are not possible or in the process of implementation, then enhancing the use of PPE (i.e., gloves, coverall, eye protection), hygiene measures (e.g., hand washing), and further restricting access during high-energy activities would be necessary. When scaling up manufacturing, investing in greater automation would greatly reduce the possibility of exposure to respirable particles, for example, automatic weighing machines and pneumatic transport from the mixer to the extrusion line.

The measurement protocol used here provides the collection of data on particle concentration and size using DRIs and morphology and chemical analysis using offline analysis, which is all contextualised to the site-specific environment and activities ongoing concurrently with those being assessed; this approach is based on existing Standards, including BSI (11) and OECD (13). Collected data, however, could also be available for control banding exercises or in more dedicated frameworks suggested for exposure management of NMs, such as the Nanomaterial Occupational Exposure Management Model (NOEM) proposed by Juric et al. (15). Here, the data collected during this measurement campaign were also used to facilitate the development of an SbD platform and to support SbD decision-making within the SAbyNA project. It is not possible to cover these specific activities here, as this study will supplement other project outputs, but it will briefly contribute to the following. These highly resolved data were introduced to specially designed (input-) templates to cover specific necessities of the platform in terms of dataset resolution and their metadata. The resulting data then fed the platform with the realistic values necessary to calibrate prediction models on emissions and the potential of exposure (fate models). These outputs are utilised to support decisions on optimal SbD solutions or to optimise the SbD approach applied during the studied case studies (tasks and processes), as well as in calculation of SbD efficiency in terms of emission reduction and quantification of risk reduction, resulting in a way forward to safer nano-processes and NEPs. The data produced here have addressed a specific life-stage (i.e., formulation and manufacture of nano-enabled filaments) and will be used to complement acquired data that cover other aspects of the life stage (3D printing, use-phase, end-of-life) with the aim of supporting the online platform to create a holistic and integrated strategy and provide the end-user with the maximum detailed information to facilitate decision-making. Collected data will also be formatted to open FAIR-aligned templates [such as those reported for the eNanoMapper database (31)] for their future application in relevant tools and models.

### Practical implementation of the tiered sampling strategy and selected methodology

There are various choices for DRI that are appropriate for different stages of a tiered sampling strategy, and it is not within capacity of this article to compare the selected DRI against other options; various reviews, guidance, and comparisons are already available (32–37). Instead, due to the added complexity of DRI used and of data analysis, and how this impacts on which stage industry may engage in assessment, we comment on how appropriate and useful it was to use methods relating to each stage of the tiered strategy. Given the aim of this study, to evaluate differences in exposure when processing a matrix containing NMs compared to a corresponding matrix without NM, the early Tier, an *initial assessment*, was considered redundant as we required quantitative comparisons. However, if it were to be observed here, it is likely that a decision to continue to later tiers would have been made. Although workplace conditions and activities would not raise concern, and with the use of masterbatch form of NMs, the potential for exposure would be considered “unlikely”, the fibrous nature and perceived high hazard of test material (SWCNT) would raise concerns in Tier 1 assessment; certainly, this would contribute to decisions on whether a measurement campaign would be suggested, but also would help prescribe the specific techniques that should be used, such as EM. When assessing the data generated here, performing Tier 3 and Tier 2 has reached the same summary results, i.e., in each case we would identify the probability of exposure as being “unlikely”. Moreover, the added complexity in Tier 3 is regarding DRIs only, as these are not particle-specific, and their use will often provide inconclusive results. However, with Tier 3 techniques, we have obtained additional information and have at times been able to link spikes in detected particle release to other unrelated activities or to when unexpected process interruptions occurred, when compared to contextual information, and were also able to better resolve exposure regarding PSD—albeit of little benefit here, given the classification of “unlikely” exposure. If this were not the case and an exposure was confirmed, this information would then have been useful.

For the purposes of SbD, performing the *basic assessment* (Tier 2) is a useful start, as it would allow a company to obtain information about probability of exposure, as this provides PNC values for comparison and further assessment, provides information from offline analysis, and can provide information on whether control measures are adequately efficient or if improvements need to be made. However, given any cause for concern, a Tier 3 study should be considered as a follow-up; the results presented here from Tier 3 have allowed a high level of confirmation that an exposure associated with the development of a NEP has not occurred and thus ensured that exposure is as low as reasonably and practically possible (ALARP). Vaquero et al. (38) also question the usefulness of DRIs due to the interference of background readings and potentially particularly large agglomerates being recorded, while offline analysis offers more informative analysis. Using DRIs of the size, weight, and number deployed in this measurement campaign are also not capable of assessing personal exposure, despite this being a focus of Tier 3, and although efforts are made to provide results that represent personal exposure (i.e., placing instrument tubes as close as reasonably practicable to the worker’s breathing zone), this will inevitably result in different results than those collected via personal monitors. Vaquero et al. (38) do, however, acknowledge that DRIs still provide useful data for risk management, as real-time monitoring allows for quantitative judgements on engineering control efficiency, but suggest real-time chemically selective sensors will be vital for assessment of NM exposures in the workplace.

The techniques used here provide measurement according to particle mass, and others by particle number. The appropriate metric to use (i.e., concentrations based on mass, surface area, or particle number concentration) when assessing exposure to NOAA and how each may help in nanosafety assessment is often discussed. There is no clear consensus on whether measurement preference should be given to the use of DRIs or to offline chemical analysis, as each will have limitations. It has for some time been expected that number- and mass-based measurements are made when emissions are in the submicron (39), and as such, we have used DRI to attain these distinctions and evidenced the presence of particles predominantly <1 μm, according to mass with use of the DustTrak and according to particle number with the APS, with FMPS being able to further resolve distributions within this size fraction. It is considered preferable to assess respirable particles when understanding exposure levels of “nanopowder-related workplaces” (39), especially when considering that airborne nano-objects may attach to larger particles (40), which we consider here with the specific size bins of the DustTrak and the size distribution data of other DRI such as APS. Conversely, it is also possible to use methods that collect respirable fractions on filters using respirable samplers (41), for example, according to standard EN13205 (42). These are then available for offline analysis, either to examine morphology and chemical fingerprint of material in the respirable size range, as was done in this study, or to quantify by gravimetric analysis. There is, however, a certain level of unreliability expected with the use of DRI, and there are necessary assumptions made when using DRI, as the instruments often provide measurements of what it assumes are single particles. When nano-objects are likely to be agglomerated or aggregated, they also assume a fixed density, and that particles are spherical (40), and this will often not be the case. This justifies the use of offline analysis as EM methods will allow the distinction of morphological differences associated with those measurements taken by DRI (40, 43, 44). Again this was evident in our SEM data. These distinctions mean that DRI will not be wholly accurate, but will provide an understanding and evidence of when fluctuation within a relevant size distribution is observed in relation to a specific activity (39). Finally, what is vital, as identified by Bekker et al. (29), is to understand the environment where measurements are being taken; recording contextual information is important, as this allows a distinction to be made with confidence between particle releases associated with the activity being examined and unrelated activities being conducted elsewhere (as we show in our case studies), with this comes a need to record suitable background measurements.

### Use of background measurements

Typically, industrial facilities have far more parallel processes ongoing (29), and are subject to traffic movement, usually open windows, etc., resulting in particles coming from other sources, these can influence baseline particle concentrations more than would be seen in laboratories or clean rooms (29). Largely due to the many different potential sources of NPs that exist in occupational settings, such as soot clusters from diesel engines or other combustion sources within the facility, from vacuum pumps or heating units (29, 45). This causes higher background values, and due to the non-specific measurement method of DRIs, it is important to conduct background measurements relevant to each process step/location. This is an important consideration, particularly when we consider that the background measurement data collected is used within the decision rules set out in the EN 17058 standard (11) for determining if the activity measurements are significant or not. Moreover, if adhering to NRVs, the PN concentration is background corrected before alignment of emission with NRV (46), and so the choice of background measurement method would likely have a significant impact on the result of breeching NRVs or not.

Some guidance is given in the protocols described previously, and others have discussed the options in order of preference (47). EN 17058 (11) states background determination will need to be situation-specific but should include simultaneous (i.e., a second instrument, likely placed in a location away from direct influence from the process) or sequential measurements (i.e., before and/or after the process involving NOAA). These options were also described in work by Boccuni et al. (48), whereby the simultaneous method is described as the FF or the “spatial” approach and the sequential method is described as the NF or “time-series” approach. Whichever method is used, the standard also recommends sampling for offline characterisation to supplement the findings and give an assessment of possible contamination. In the work by Basinas et al. (47), a third method of background measurement is described, which involves measurement during the same activity when no NOAA is present.

We have been able to test all three background evaluation methods here, including sequential measurements, simultaneous measurement, and measurement of the same process but with a masterbatch that contained no nano-additives. Due to the, in general, low emissions recorded during activities, we found that regardless of method used, our measurements were typically below cut-off values defined by all backgrounds. When we observed increased activity-related particle release greater than background cut-off values, it was mostly when compared to the sequential backgrounds and often to measurements with CPC, which is particularly relevant to the previous discussion as it is observations such as this made with DRI key to Tier 2 assessment will dictate that a user move further and conduct a Tier 3 study (13). There is disparity between values generated by these different approaches; the use of “same activity with no NOAA present” (i.e., when filament was produced containing no nano-additive) consistently provided the higher background value compared to sequential measurements; for example, during filament production, we found a 1.8-fold, 1.5-fold, 1.2-fold, and 2.5-fold difference in background value between these two methods for FMPS, APS, DustTrak, and DiSCmini, respectively. CPC was the only DRI that we were able to use all three techniques; sequential measurements provided 1.7-fold increase in background value compared to simultaneous, while when using “same activity with no NOAA present” we observed a 2.9-fold and 1.7-fold increase compared to simultaneous and sequential, respectively. These differences highlight the importance of selecting which background comparison to make; note that it is equally important to link these observations to the contextual information collected.

Basinas et al. (47) consider the “same activity with no NOAA present” to be the most appropriate method to use in comparisons. This approach, although it ignores unrelated processes ongoing within the room/facility at the time of measurement, does provide a clear distinction of what the impact will be of including an NM in the process/product; we support this conclusion. This approach provided us with the highest background level, and although this is less likely to result in breach of an NRV (due to the high background correction), it is probably the most informative when addressing the release caused by the addition of NM to NEPs. Moreover, when we had observed increases in activity-related release data (e.g., in weighing PC and PC with SWCNT) as a result of comparisons to the sequential method of background setting, it was necessary to make committed comparisons between the specific time-stamped release data for each relevant activity, compare each to contextual information, and further to offline analysis before being able to reach a conclusion that release was at times process-related and not specifically caused by the presence of NM within the masterbatch; a background set to the “same activity with no NOAA present” circumvents some of this, and for our specific research question, this is probably the most suitable.

However, in some process(es) steps, the collection of background based on the “same activity with no NOAA present” is not possible (e.g., weighing, bagging, and dispensing nanopowders); therefore, other options should be used. In this case, Basinas et al. (47) note that measurement before/after activity approach is the least accurate method, as it does not account for process-generated NOAAs nor does it account for those produced elsewhere in the facility while the measurement is being conducted. Whereas measurement of background simultaneously in an FF location [FF approach (48)] is the more suitable alternative, as although it cannot differentiate between process-generated NOAA, it can account for NOAA produced elsewhere in the facility while the process is running. Therefore, fluctuations in particle concentration due to non-process-generated events such as staff moving around the facility [as evidenced by Boccuni et al. (48)], changes in airflow (e.g., due to doors or windows opening), or environmental changes (e.g., temperature or humidity) will be captured and can be corrected for in the subsequent data analysis. This recommendation is mirrored by the guidance given in the NEAT 2.0 methodology (28), whereby the simultaneous method is suggested.

In the case studies we describe here, the background PNC was notably high. We have already discussed that this may contribute to uncertainty. However, no guidance is available on the actions to be taken when this is the case, emphasising the need to collect offline samples and the importance of these samples when assessing likelihood of exposure.

### Offline analysis options

With regard to collecting samples for subsequent offline analysis (11), it suggests various possible options such as atomic force microscopy (AFM), electron energy loss spectroscopy (EELS), scanning probe microscopy (SPM), NF scanning optical microscopy (NSOM), total reflection X-ray fluorescence spectroscopy (TXRF), ICP-MS, and inductively coupled plasma atomic emission spectroscopy (ICP-AES). However, it is noted that electron microscopy (EM) is the most common technique (TEM or SEM). These have specific limitations: neither can really confirm the absence of NPs, given the limited field-of-view used during assessment; for example, in the work by Brouwer et al. (49), TEM was used qualitatively to indicate the presence of NOAA, as quantification of NOAA would be “very time consuming.” In 2012, discussions from a workshop on harmonisation of strategies to measure and analyse exposure to NOAA described quantitative TEM analysis as “not suitable for routine analysis” due to the cost and time required, as well as the methodology used at that time being subjective due to a lack of standardised methods (50). Moreover, neither will be able to sufficiently assess NMs embedded within a matrix (especially SEM), and it is very probable that this is what we would have collected during our measurement campaign. It has been reported that AFM is able to characterise particles in this manner (51). More recently, Raman spectroscopy has been developed for use in the field using portable samplers, which can detect specific nanomaterials if the raw material is available for calibration and have the option for further analysis such as SEM (52).

The metric requirements for BSI (11), as specified in BSI (10), are that time-integrated sampling of the respirable fraction should provide a mass concentration by chemical analysis (i.e., using AFM, EELS, SPM, NSOM, TXRF, ICP-MS, and ICP-AES), but where doubt exists on the presence of NOAAs within this mass concentration, or the chemical method is not sensitive enough for quantifying the mass concentration of NOAAs, the chemical analysis shall be completed by collecting a sample for EM (i.e., by use of energy-dispersive X-ray analysis [EDX]). Here we have first used ICP-AES for detection of chemical markers of the NM within NEPs, Ag and Fe (as a contaminant of the SWCNT); given the relatively high LOD for ICP-AES in comparison to the amounts of either Ag or Fe expected to be present, it is not surprising that we found no evidence of these markers, and in cases of NEPs, it is likely that ICP-MS would be required. We also collected the same samples for SEM analysis; this is a useful technique for observations of larger size fractions, but again, it was found unsuitable to resolve the smaller size fractions observed in our DRI data, so it should be combined with TEM analysis and not proposed as an alternative. Even with the limitations previously mentioned, TEM will provide a better opportunity to identify the association (matrix-bound) and disassociation of NM with matrix (53). Although there are still no nano-specific standards currently available for TEM analysis, adaptations of methods for asbestos analysis have been followed, such as the NIOSH 7402 method (54)—followed by Garcia et al. (55), MHDS 87 from the Health & Safety Executive in the UK (56), and OECD TG No. 125 (57). NIOSH have also described a method for TEM analysis for the detection of CNT/CNF, available in Chapter CN of the NIOSH Manual of Analytical Methods (NMAM) (58).

## Conclusion

A measurement campaign was completed for the assessment of excess particle emissions associated with the addition of NMs to filaments produced for the additive manufacturing industry. The campaign was structured according to the current European Standard for occupational exposure monitoring of NOAA (11) and generated data to be used in the development of the new SAbyNA SbD platform to assist companies in SbD decision-making processes. With the use of a range of recognised methods for assessing background emissions compared and contrasted, logical decisions could be made on particle release, and it was found that the addition of NMs (Ag to PP and SWCNT to PC) to filament production did not increase particle release. Process-related particle release was noted, and improvements to the RMMs were provided, including improving LEV systems and enhancing the use of PPE and hygiene measures.

In comparison to the various methodologies available, we identified that DRIs are heavily represented and available to address the higher tiers of the tiered exposure strategies, while offline measurements are potentially less reliable. The lack of suitable standards for assessing NOAA by EM has already been mentioned, and this is also true for chemical analysis. The only standardised methods available that have been recommended in nano-specific guidance are NIOSH Method 5,040 for elemental carbon analysis to quantify CNT/CNF release and NIOSH Method 7,300 for Ag or TiO_2_ concentration by ICP-AES (12), and as previously mentioned, these can be used alongside REL values set for CNT, Ag, and TiO_2_ until OELs are available and set in regulation (19–21). For all other NOAAs, no such RELs have been determined, and therefore limited information is available in the literature for the suitability of current methods for detecting NOAAs at achievable instrument detection limits. These absences are a major barrier to the implementation of robust NOAA exposure assessment. Particularly when we consider that the use of offline analysis is critical for the decision rules outlined in BSI (11). It has been proposed that to be considered a high-quality study conducting NOAA exposure assessment, it should be required that both DRIs and offline analysis should be used, or offline analysis can be sufficient alone if a “well-established chemical analysis method available and used to quantify release” (47). However, how feasible this is, is highly dependent on available chemical analysis methods showing suitable sensitivity for quantifying NOAAs as well as aerosol samplers showing suitable collection efficiencies for particles in the nanoscale. As such, currently, the optimal approach is the use of a combination of offline and online analysis.

## Data availability statement

The original contributions presented in the study are included in the article/Supplementary material, further inquiries can be directed to the corresponding author.

## Author contributions

PM: Conceptualization, Data curation, Formal analysis, Investigation, Methodology, Writing – original draft, Writing – review & editing. JH: Conceptualization, Data curation, Formal analysis, Investigation, Project administration, Writing – review & editing. AS: Writing – review & editing. KG: Writing – review & editing. FB: Data curation, Software, Writing – review & editing. CC: Investigation, Writing – review & editing. DM: Investigation, Writing – review & editing. SV-C: Funding acquisition, Writing – review & editing. DL: Conceptualization, Investigation, Writing – review & editing. MB: Conceptualization, Data curation, Formal analysis, Project administration, Supervision, Writing – original draft, Writing – review & editing.
