# Supplementary material for: Safe(r)-by-design principles in the thermoplastics industry: guidance on release assessment during manufacture of nano-enabled products
Source: Front Public Health. 2024 Jul 5;12:1398104. doi: 10.3389/fpubh.2024.1398104 (PMC11257848; doi:10.3389/fpubh.2024.1398104)
Supplement: Supplementary file 1 [file Data_Sheet_1.docx]

Supplementary Material

Safe(r)-by-Design principles in the thermoplastics industry; guidance on release assessment during manufacture of nano-enabled products.

Polly McLean^1*^, James Hanlon^1,2^, Apostolos Salmatonidis^3^, Karen S. Galea^1^, Finlay Brooker^1^, Cristiano Citterio^4^, Daniele Magni^4^, Socorro Vázquez-Campos^3^, Davide Lotti^4^, Matthew S.P. Boyles^1,5^

^1^Institute of Occupational Medicine, Edinburgh, UK

^2^Ricardo, Glasgow, UK

^3^LEITAT Technological Center, Terrassa, Spain;

^4^LATI Industria Termoplastici S.p.A., Vedano Olona (VA), Italy;

^5^Centre for Biomedicine and Global Health, School of Applied Sciences, Edinburgh Napier University, Edinburgh, UK

***Correspondence:**Polly.McLean@iom-world.org

Keywords: safe(r)-by-design (SbD), nano-enabled products (NEPs), nanomaterials, nanoparticles, nano-objects, exposure assessment, 3D printing, additive manufacturing.

# Exposure Scenarios

Processes examined were conducted across two separate rooms. Exposure Scenarios 1, 2 (relating to weighing), 3, 4, 5 (relating to mixing), 8, 9, and 10 (relating to filament production) were conducted in one room, while Exposure Scenarios 6 and 7 (relating to extrusion) were performed in another room at a separate plant location.

## Nano silver (Ag) and polypropylene (PP) filament manufacturing (case study 1)

**Exposure Scenario 1: Weighing of PP (blank).** A total of 9 kg of PP was weighed. The weighed PP is then used later in exposure scenario 3, when mixed with additives and dosed, or is fed directly, by pouring, into the extruder (exposure scenario 6). No nano Ag was used. This is a manual, open process and local exhaust ventilation (LEV) is used. The weighing area is 84 m^2^. The weighing took place in the morning (time stamp 10:25:51-10:28:21). This is a manual and open process with LEV used. Windows were open in the facility to provide natural ventilation, with additional ventilation from a downdraft table for small batches and LEV on top of the larger balance. The temperature in the facility was 27 ± 1 °C and relative humidity was 55 ± 2 %. One operator performed the weighing (scoop used for weighing) and one additional operator helped to retrieve chemicals. Gloves were not worn during this exposure scenario.

**Exposure Scenario 2: Weighing of nano Ag.** A total of 4.8 kg of nano Ag masterbatch was weighed. This is a manual and open process with LEV used. The weighing area is 84 m^2^. The weighing took place in the morning (time stamp 10:28:51-10:29:21). This is a manual and open process with LEV used. Windows were open in the facility to provide natural ventilation. The temperature in the facility was 27 ± 1 °C and relative humidity was 55 ± 2 %. One operator performed the weighing (scoop used for weighing). Gloves were not worn during this exposure scenario.

**Exposure Scenario 3: Mixing of PP.** A total of 4 kg of PP was mixed. The PP used here is mixed, or fed, by pouring, directly into the extruder (exposure scenario 6). This took place in the morning (time stamp 11:21:38-11:26:43). This process involved the operator adding, by pouring, the weighed PP manually. During the automated mixing process, the operator stands beside the mixing equipment. The operator transfers the contents of the mixing hopper into a bag. The room area for mixing is 98 m^2^. Windows were open in the facility to provide natural ventilation. The temperature in the facility was 29 ± 1 °C and relative humidity was 50 ± 1 %.

**Exposure scenario 4: Mixing of nano Ag and PP.** A total of 9 kg of nano Ag masterbatch, PP and a non-nano stabilizer were mixed. This took place in the morning (time stamp 11:27:06-11:33:35). This process involved the operator adding the weighed PP manually. During the automated mixing process, the operator stands beside the mixing equipment. The operator transfers the contents of the mixing hopper into a bag. The room area for mixing is 98 m^2^. Windows were open in the facility to provide natural ventilation. The temperature in the facility was 29 ± 1 °C and relative humidity was 50 ± 1 %.

**Exposure scenario 5: Cleaning equipment after mixing.** This involved the operator using a vacuum cleaner to clean the mixing equipment (time stamp 11:33:44-11:35:01).

**Exposure scenario 6: Extrusion of PP.** A total of 60 kg of PP compound was extruded. The extrusion took place in a facility which contained other operational extruders and was adjacent to one of the main access points to the plant where facility-related traffic would pass. This took place in the afternoon (time stamp 14:05:53-15:08:03). Windows/doors were open in the facility to provide natural ventilation. The temperature in the facility was 33 ± 2 °C and relative humidity was 40 ± 3 %. The PP and other components were fed automatically from the hopper located on a mezzanine, although the process for feeding the hopper was performed manually by pouring. Gloves and respiratory protective equipment (FFP3) were used by the operator during all of the extrusion processes.

**Exposure scenario 7: Extrusion of nano Ag and PP.** A total of 60 kg of nano Ag / PP compound were extruded. This extrusion took place in the same facility as exposure scenario 6 (time stamp 15:22:20-16:21:48). Windows/doors were open in the facility to provide natural ventilation. The temperature in the facility was 34 ± 1 °C and relative humidity was 36 ± 1 %. The PP + nano Ag mixture and other non-nano components were fed automatically from the hopper located on a mezzanine, although the process for feeding the feeder was performed manually, by pouring, where dust was generated. The extruded product was collected in 25 kg bags, with 1-2 operators manually handling bags.

**Exposure scenario 8: Filament production of PP.** The PP pellets produced in the extrusion step (Exposure scenario 6) were fed into the Microex 3D filament (Eur.Ex.Ma) extrusion line to produce filaments suitable for 3D printing. This took place in the same facility as mixing (Exposure scenarios 3 and 4), in the afternoon (time stamp 13:35:00-14:12:52). The room area for mixing is 98 m^2^. Windows/doors were open in the facility to provide natural ventilation. Temperature in the facility was 26 ± 2 °C and relative humidity was 70 ± 4 %. This process was performed manually by one operator and involved filling up hopper, stirring the hopper, adjusting the flow of the filament, and changing spool. Heat is produced from the instrument during melting of the PP pellets and often the casing of the instrument would need to be lifted to make adjustments. Activity at a nearby line in the same room was also observed.

**Exposure scenario 9: Filament production of nano Ag and PP.** The PP + nano Ag pellets produced in the extrusion step (Exposure scenario 7) were fed into Microex 3D filament (Eur.Ex.Ma) extrusion line to produce filaments suitable for 3D printing. This took place in the same facility as mixing (Exposure scenarios 3 and 4), in the afternoon (time stamp 14:12:53-14:40:50). The room area for mixing is 98 m^2^. Windows/doors were open in the facility to provide natural ventilation. Temperature in the facility was 27 ± 2 °C and relative humidity was 64 ± 2 %. This process involved manual filling of hopper, stirring the hopper, adjusting the flow of the filament, and changing spool. Heat is produced from the instrument during melting of the PP + nano Ag pellets and often the casing of the instrument would need to be lifted to make adjustments. There was also some cleaning being performed at the nearby line at the beginning of this exposure scenario.

**Exposure scenario 10: Cleaning after filament production.** This was performed after filament production and was performed for a short period of <5 min. This involved using HDPE (High Density Polyethylene, not modified) to remove the majority of compounds previously extruded; all other steps were similar to normal extrusion process of the machine.

## Single walled carbon nanotubes (SWCNT) and polycarbonate (PC) filament manufacturing (case study 2) - steps for case study 2 were conducted in same locations as case study 1 processes.

**Exposure scenario 1: Weighing of PC.** A total of 5.8 kg of PC was weighed. This amount is for feeding the minor additives in the formulation, with the rest of the PC fed directly into the extruder (exposure scenario 5/6). No SWCNT were used. The weighing took place in the morning (time stamp 10:32:16-10:38:16). The weighing area is 84 m^2^. Windows were open in the facility to provide natural ventilation. The temperature in the facility was 27 ± 1 °C and relative humidity was 55 ± 2 %. One operator performed the weighing (scoop used for weighing) and one additional operator to retrieve chemicals. Ventilation was provided by a downdraft table for small batches and LEV on top of the larger balance. Gloves were not worn during this exposure scenario.

**Exposure scenario 2: Weighing of SWCNTs.** A total of 1 kg g of SWCNTs concentrate was weighed. The weighing took place in the morning (time stamp 10:32:16-10:38:16). The weighing area is 84 m^2^. Windows were open in the facility to provide natural ventilation. The temperature in the facility was 27 ± 1 °C and relative humidity was 55 ± 2 %. One operator performed the weighing (scoop used for weighing). Gloves were not worn during this exposure scenario. Ventilation was provided by a downdraft table for small batches and LEV on top of the larger balance.

**Exposure scenario 3: Mixing of PC.** A total of 5.8 kg of PC was mixed with minor additives (not including nanoforms). This process involved the operator adding the weighed components manually by pouring. This took place in the morning (time stamp 11:35:13-11:40:15). During the automated mixing process, the operator stands beside the mixing equipment. The operator transfers the contents of the mixing hopper into a 25 kg bag. It was observed this dust was generated during this process. The room area for mixing is 98 m^2^. Windows were open in the facility to provide natural ventilation. The temperature in the facility was 29 ± 1 °C and relative humidity was 49 ± 1 %.

**Exposure scenario 4: Mixing of SWCNT and PC.** A total of 4.8 kg of PC was mixed with SWCNT concentrate and minor additives (not including nanoforms). This process involved the operator adding the weighed PC manually. This took place in the morning (time stamp 11:35:13-11:40:15). During the automated mixing process, the operator stands beside the mixing equipment. The operator transfers the contents of the mixing hopper into a bag. It was observed this dust was generated during this process. The room area for mixing is 98 m^2^. Windows were open in the facility to provide natural ventilation. The temperature in the facility was 29 ± 1 °C and relative humidity was 49 ± 1 %.

**Exposure scenario 5: Extrusion of PC.** A total of 60 kg PC compound were extruded. The extrusion took place in a facility (time stamp 09:01:21-10:08:55) which contained other operational extruders and was adjacent to one of the main access points to the plant where facility-related traffic would pass by (e.g. forklifts). Windows/doors were open in the facility to provide natural ventilation. The temperature in the facility was 28 ± 1 °C and relative humidity was 55 ± 4 %. The PC was fed automatically from the top feeder located on a mezzanine, although the process for feeding the feeder was performed manually, when a bag is tilted by an operator from the top of the feeder cup hopper, where dust was generated, additional two feeders were used for the mixture PC and minor additives and for a further non-nano additive. The obtained product was collected in 25 kg bags (granules). During this extrusion process, it was observed that there was a lot of activity at the nearby extrusion line, for products unrelated to the case study.

**Exposure scenario 6: Extrusion of SWCNT and PC.** A total of 60 kg SWCNT and PC were extruded. This extrusion took place (time stamp 10:11:05-11:06:15) in the same facility as exposure scenario 5. Windows/doors were open in the facility to provide natural ventilation. The temperature in the facility was 29 ± 2 °C and relative humidity was 54 ± 3 %. The PC and SWCNT was fed automatically from the top feeder located on a mezzanine, although the process for feeding the feeder was performed manually (when a bag is tilted by an operator from the top of the feeder cup hopper) where dust was generated, additional two feeders were used for the mixture PC + SWCNT + minor additives and for a further non-nano additive. The obtained product was collected in 25 kg bags (granules); no gloves were worn for handling the final product. During this extrusion process, it was observed that there was a lot of activity at the nearby extrusion line, for products unrelated to the case study.

**Exposure scenario 7: Cleaning after extrusion.** Performed after the extrusion of the SWCNT-PC filament and involved cleaning the extruder line with HDPE. This was performed for a short period (time stamp 10:08:55-10:11:05) with a spraying gun to remove residue from the pelletiser with gloves also worn by the operator.

**Exposure scenario 8: Filament production of PC.** The PC pellets produced in the extrusion step (Exposure scenario 5) were fed into the Microex 3D filament (Eur.Ex.Ma) extrusion line to produce filaments suitable for 3D printing. This took place in the afternoon (time stamp 14:47:05-15:28:08) in the same facility as mixing (Exposure scenarios 3 and 4). Windows/doors were open in the facility to provide natural ventilation. The room area is 98 m^2^. The temperature in the facility was 28 ± 1 °C and relative humidity was 60 ± 2 %. This process involved filling up hopper, stirring the hopper, adjusting the flow of the filament, and changing spool. Heat is produced from the instrument during melting of the PC pellets. The bath casing was opened at various points during the measurement due to issues encountered.

**Exposure scenario 9: Filament production of SWCNT and PC.** The PC+SWCNT pellets produced in the extrusion step (Exposure scenario 6) were fed into the Microex 3D filament (Eur.Ex.Ma) extrusion line to produce filaments suitable for 3D printing. This took place in the afternoon (time stamp 15:28:08-16:06:07) in the same facility as mixing (Exposure scenarios 3 and 4). Windows/doors were open in the facility to provide natural ventilation. The room area is 98 m^2^. The temperature in the facility was 28 ± 1 °C and relative humidity was 56 ± 1 %. This process conducted manually and involved filling up hopper, stirring the hopper, adjusting the flow of the filament, and changing spool. Heat is produced from the instrument during melting of the PC+SWCNT pellets. The bath casing was opened at various points during the measurement to manually adjust the polymer melt path during cooling, the extrusion process was shortly stopped during this adjustment.

# Summary Tables

Table S1 Summary data of case study 1, production of nano Ag and PP filament. Includes detection of particles by different direct reading instruments (DRI) during weighing, mixing, cleaning, extrusion, and final filament production; incorporates background controls to provide a cut-off value to allow identification of overall likelihood of exposure; analytical detection of chemical markers of released material measured by ICP-AES (Ag detection).

| **Exposure Scenario (ES)** | **ICP/AES (Ag)** | **Instrument (metric)** | **Average** | **Maximum** | **Minimum** | **Cut off value^a^** | **Cut off value^b^** | **Conclusion (Likelihood of exposure induced by the nanomaterial activity)** |
| --- | --- | --- | --- | --- | --- | --- | --- | --- |
| **Exposure Scenario 1: Weighing of PP (blank).**  **NF**: N/A **Polymer:** PP  **Activity:** Weighing **Duration:** 2 mins 30 secs **Control:** LEV | <LOD | FMPS (N, #cm^3^) | 53186 | 71796 | 42513 | 86854 | NA | unlikely |
|  |  | APS (N, 0.5 -20 μm) | 343 | 442 | 269 | 452 | NA |  |
|  |  | CPC NF (#cm^3^) | 5237 | 5840 | 4937 | 5564 | NA |  |
|  |  | DustTrak (mg/m^3^) | 0.023 | 0.084 | 0.018 | 0.039 | NA |  |
|  |  | DiSCmini (#cm^3^) | 3223 | 7395 | 2697 | 10790 | NA |  |
| **Exposure Scenario 2: Weighing of nano Ag.**  **NF**: Ag **Polymer:** n/a  **Activity:** Weighing **Duration:** 1 min **Control:** LEV | <LOD | FMPS (#cm^3^) | 52186 | 71796 | 42513 | 86854 | 69565 | unlikely |
|  |  | APS (0.5 -20 μm) | 343 | 422 | 275 | 452 | 428 |  |
|  |  | CPC NF (#cm^3^) | 5194 | 5603 | 4915 | 5565 | 5596 |  |
|  |  | DustTrak (mg/m^3^) | 0.022 | 0.043 | 0.018 | 0.039 | 0.047 |  |
|  |  | DiSCmini (#cm^3^) | 3141 | 6434 | 2732 | 10790 | 4064 |  |
| **Exposure Scenario 3: Mixing of PP.**  **NF**: N/A **Polymer:** PP **Activity:** Mixing **Duration:** 5 mins 5 secs **Control:** LEV | <LOD | FMPS (#cm^3^) | 67457 | 90548 | 53807 | 85343 | NA | unlikely |
|  |  | APS (0.5 -20 μm) | 285 | 361 | 226 | 367 | NA |  |
|  |  | CPC NF (#cm^3^) | 5273 | 5769 | 4921 | 5784 | NA |  |
|  |  | DustTrak (mg/m^3^) | 0.021 | 0.06 | 0.017 | 0.035 | NA |  |
|  |  | DiSCmini (#cm^3^) | 3132 | 8305 | 2629 | 10790 | NA |  |
| **Exposure Scenario 4: Mixing of nano Ag and PP.**  **NF**: Ag **Polymer:** PP **Activity:** Mixing **Duration:** 6 mins 19 secs **Control:** LEV | <LOD | FMPS (#cm^3^) | 66454 | 82872 | 47574 | 85343 | 84965 | unlikely |
|  |  | APS (#cm^3^) | 292 | 367 | 217 | 367 | 354 |  |
|  |  | CPC NF (#cm^3^) | 5278 | 5531 | 5006 | 5784 | 5822 |  |
|  |  | DustTrak (mg/m^3^) | 0.022 | 0.066 | 0.017 | 0.035 | 0.036 |  |
|  |  | DiSCmini (#cm^3^) | 3223 | 7395 | 2697 | 10790 | 4494 |  |
| **Exposure Scenario 5: Cleaning.**  **NF**: N/A **Product:** N/A **Activity:** Cleaning **Duration:** 17 secs **Control:** LEV | <LOD | FMPS (#cm^3^) | 66241 | 74985 | 56160 | 85343 | NA | unlikely |
|  |  | APS (#cm^3^) | 320 | 470 | 250 | 367 | NA |  |
|  |  | CPC NF (#cm^3^) | 5110 | 5320 | 4910 | 5784 | NA |  |
|  |  | DustTrak (mg/m^3^) | 0.026 | 0.094 | 0.018 | 0.035 | NA |  |
|  |  | DiSCmini (#cm^3^) | 3141 | 6434 | 2732 | 10790 | NA |  |
| **Exposure Scenario 6: Extrusion of PP.**  **NF**: N/A **Product:** Polypropylene **Activity:** Extrusion **Duration:** 1 hr 2 mins 10 secs **Control:** LEV | <LOD | FMPS (#cm^3^) | 156968 | 1500382 | 69504 | 442576 | NA | unlikely |
|  |  | APS (#cm^3^) | 319 | 1096 | 201 | 596 | NA |  |
|  |  | DustTrak (mg/m^3^) | 0.026 | 0.147 | 0.015 | 0.091 | NA |  |
|  |  | DiSCmini (#cm^3^) | 10884 | 39111 | 5608 | 11466 | NA |  |
| **Exposure Scenario 7: Extrusion of nano Ag and PP.**  **NF**: Ag **Product:** Polypropylene **Activity:** Extrusion **Duration:** 59 mins 28 secs **Control:** LEV | <LOD | FMPS (#cm^3^) | 178909 | 524997 | 65894 | 442576 | 373216 | unlikely |
|  |  | APS (#cm^3^) | 317 | 1096 | 201 | 596 | 486 |  |
|  |  | DustTrak (mg/m^3^) | 0.033 | 0.22 | 0.017 | 0.091 | 0.051 |  |
|  |  | DiSCmini (#cm^3^) | 8304 | 48286 | 5760 | 11466 | 22029 |  |
| **Exposure Scenario 8: Filament production of PP.**  **NF**: N/A **Product:** Polypropylene **Activity:** Filament production **Duration:** 37 mins 52 secs **Control:** LEV | <LOD | FMPS (#cm^3^) | 793420 | 3588506 | 109693 | 1575682 | NA | unlikely |
|  |  | APS (#cm^3^) | 1183 | 5138 | 636 | 1974 | NA |  |
|  |  | CPC NF (#cm^3^) | 45491 | 168598 | 6290 | 87799 | NA |  |
|  |  | DustTrak (mg/m^3^) | 0.047 | 0.176 | 0.028 | 0.087 | NA |  |
|  |  | DiSCmini (#cm^3^) | 29276 | 188347 | 3764 | 51598 | NA |  |
| **Exposure Scenario 9: Filament production of nano Ag and PP.**  **NF**: Ag **Product:** Polypropylene **Activity:** Filament production **Duration:** 27 mins 57 secs  **Control:** LEV | <LOD | FMPS (#cm^3^) | 488977 | 2894008 | 114120 | 1575682 | 2863679 | unlikely |
|  |  | APS (#cm^3^) | 1548 | 3259 | 925 | 1974 | 2953 |  |
|  |  | CPC NF (#cm^3^) | 29712 | 131264 | 6100 | 87799 | 150018 |  |
|  |  | DustTrak (mg/m^3^) | 0.048 | 0.176 | 0.028 | 0.087 | 0.107 |  |
|  |  | DiSCmini (#cm^3^) | 17686 | 150051 | 4162 | 51598 | 128306 |  |
| **Exposure Scenario 10: Cleaning.**  **NF**: Ag **Product:** Polypropylene **Activity:** Cleaning **Duration:** 15 mins 35 secs **Control:** LEV | N/A | FMPS (#cm^3^) | 658803 | 12418485 | 115408 | 1575682 | NA | unlikely |
|  |  | APS (#cm^3^) | 1144 | 1894 | 509 | 1974 | NA |  |
|  |  | CPC NF (#cm^3^) | 37166 | 311988 | 6233 | 87799 | NA |  |
|  |  | DustTrak (mg/m^3^) | 0.045 | 0.102 | 0.036 | 0.087 | NA |  |
|  |  | DiSCmini (#cm^3^) | 34456 | 1056697 | 4308 | 51598 | NA |  |

^a^ Using background value determined via sequential method.

^b^ Using background value determined via sampling of the same activity when masterbatch contained no NM.

Table S2 Summary data of case study 2, production of SWCNT and PC filament. Includes detection of particles by different direct reading instruments (DRI) during weighing, mixing, cleaning, extrusion, and final filament production; incorporates background controls to provide a cut-off value to allow identification of overall likelihood of exposure; analytical detection of chemical markers of released material measured by ICP-AES (Fe detection); scanning electron microscope (SEM) analysis of collected filters. Averaged activity values in bold or underlined are those in which the measured release during activity exceed cut off values based on a background value determined by the sequential or ‘no NOAA’ methods, respectively.

| **Exposure Scenario** | **ICP/AES (Fe)** | **SEM** | **Instrument (metric)** | **Average** | **Maximum** | **Minimum** | **Cut off value^a^** | **Cut off value^b^** | **Conclusion (Likelihood of exposure induced by the nanomaterial activity)** |
| --- | --- | --- | --- | --- | --- | --- | --- | --- | --- |
| **Exposure Scenario 1:** **Weighing of PC.**  **NF**: N/A **Polymer:** Polycarbonate **Activity:** Weighing **Duration:** 6 mins **Control:** LEV | <LOD | N/A | FMPS (N, #cm^3^) | 62973 | 142222 | 53357 | 86854 | NA | unlikely |
|  |  |  | APS (N, #cm^3^) | 398 | 606 | 298 | 452 | NA |  |
|  |  |  | CPC NF (#cm^3^) | **5865** | 6130 | 5458 | 5565 | NA |  |
|  |  |  | DustTrak (mg/m^3^) | **0.053** | 0.277 | 0.019 | 0.039 | NA |  |
|  |  |  | DiSCmini (#cm^3^) | 3220 | 4663 | 2591 | 10790 | NA |  |
| **Exposure Scenario 2: Weighing of SWCNTs.**  **NF**: SWCNT **Polymer:** N/A **Activity:** Weighing **Duration:** 5 mins **Control:** LEV | <LOD | N/A | FMPS (#cm^3^) | 63409 | 83482 | 53357 | 86854 | 81298 | unlikely |
|  |  |  | APS (#cm^3^) | 372 | 459 | 269 | 452 | 532 |  |
|  |  |  | CPC NF (#cm^3^) | **5855** | 6112 | 5537 | 5565 | 6187 |  |
|  |  |  | DustTrak (mg/m^3^) | 0.034 | 0.101 | 0.02 | 0.039 | 0.181 |  |
|  |  |  | DiSCmini (#cm^3^) | 3471 | 316483 | 2484 | 10790 | 7421 |  |
| **Exposure Scenario 3: Mixing of PC.**  **NF**: N/A **Polymer:** Polycarbonate **Activity:** Mixing **Duration:** 5 mins 2 secs **Control:** LEV | <LOD | N/A | FMPS (#cm^3^) | 63602 | 74323 | 53838 | 85343 | NA | unlikely |
|  |  |  | APS (#cm^3^) | 294 | 364 | 239 | 367 | NA |  |
|  |  |  | CPC NF (#cm^3^) | 4992 | 5188 | 4805 | 5784 | NA |  |
|  |  |  | DustTrak (mg/m^3^) | 0.021 | 0.039 | 0.017 | 0.035 | NA |  |
|  |  |  | DiSCmini (#cm^3^) | 3064 | 4663 | 2591 | 10790 | NA |  |
| **Exposure Scenario 4: Mixing of SWCNT and PC.**  **NF**: SWCNT **Polymer:** Polycarbonate **Activity:** Mixing **Duration:** 8 mins 28 secs **Control:** LEV | <LOD | N/A | FMPS (#cm^3^) | 63423 | 74523 | 52297 | 85343 | 74668 | unlikely |
|  |  |  | APS (#cm^3^) | 293 | 407 | 233 | 367 | 368 |  |
|  |  |  | CPC NF (#cm^3^) | 5025 | 5292 | 4707 | 5784 | 5212 |  |
|  |  |  | DustTrak (mg/m^3^) | 0.023 | 0.103 | 0.018 | 0.035 | 0.031 |  |
|  |  |  | DiSCmini (#cm^3^) | 4519 | 316483 | 2484 | 10790 | 3838 |  |
| **Exposure Scenario 5: Extrusion of PC.**  **NF**: N/A **Polymer:** Polycarbonate **Activity:** Extrusion **Duration:** 1 hr 7mins 34 secs  **Control:** LEV | <LOD | N/A | FMPS (#cm^3^) | 357761 | 6226485 | 92514 | 666690 | NA | unlikely |
|  |  |  | APS (#cm^3^) | 1406 | 7465 | 582 | 1591 | NA |  |
|  |  |  | CPC NF (#cm^3^) | **24906** | 99955 | 9653 | 16844 | NA |  |
|  |  |  | DustTrak (mg/m^3^) | **0.071** | 0.41 | 0.025 | 0.062 | NA |  |
|  |  |  | DiSCmini (#cm^3^) | **15459** | 32314 | 5396 | 15424 | NA |  |
| **Exposure Scenario 6: Extrusion of SWCNT and PC.**  **NF**: SWCNT **Polymer:** Polycarbonate **Activity:** Extrusion **Duration:** 56 mins 22 secs **Control:** LEV | <LOD | Extruder: no SWCNT  Hopper sample: no SWCNT  Personal sample: Similar morphology to SWCNT masterbatch, no Fe detected | FMPS (#cm^3^) | 262708 | 567101 | 149548 | 666690 | 1143603 | unlikely^c^ |
|  |  |  | APS (#cm^3^) | 1322 | 2627 | 617 | 1591 | 4496 |  |
|  |  |  | CPC NF (#cm^3^) | **17333** | 46522 | 9434 | 16844 | 66908 |  |
|  |  |  | DustTrak (mg/m^3^) | 0.045 | 0.091 | 0.029 | 0.062 | 0.239 |  |
|  |  |  | DiSCmini (#cm^3^) | **18266** | 37951 | 21.22 | 15424 | 30816 |  |
| **Exposure Scenario 7: Cleaning after extrusion.**  **NF**: N/A  **Polymer:** N/A **Activity:** Cleaning after extrusion  **Duration:** 10 secs **Control:** PPE only | <LOD | N/A | FMPS (#cm^3^) | 368208 | 680322 | 247091 | 666690 | NA | unlikely |
|  |  |  | CPC NF (#cm^3^) | **27577** | 42852 | 21658 | 16844 | NA |  |
|  |  |  | DustTrak (mg/m^3^) | 0.050 | 0.087 | 0.042 | 0.061 | NA |  |
| **Exposure Scenario 8: Filament production of PC.**  **NF**: N/A **Polymer:** Polycarbonate **Activity:** Filament production **Duration:** 31 mins 3 secs **Control:** LEV | <LOD | N/A | FMPS (#cm^3^) | 833129 | 35082061 | 95100 | 1575682 | NA | unlikely |
|  |  |  | APS (#cm^3^) | 396 | 830 | 268 | 1974 | NA |  |
|  |  |  | CPC NF (#cm^3^) | 36455 | 360443 | 4663 | 87799 | NA |  |
|  |  |  | DustTrak (mg/m^3^) | 0.041 | 0.072 | 0.027 | 0.087 | NA |  |
|  |  |  | DiSCmini (#cm^3^) | 35044 | 2711276 | 2852 | 51598 | NA |  |
| **Exposure Scenario 9: Filament production of SWCNT and PC.**  **NF**: SWCNT **Polymer**: Polycarbonate **Activity**: Filament production Duration: 37 mins 59 secs  **Control**: LEV | <LOD | NF: no SWCNT  FF: no SWCNT  Personal: no SWCNT | FMPS (#cm^3^) | 1051122 | 27859678 | 83489 | 1575682 | 6734336 | unlikely |
|  |  |  | CPC NF (#cm^3^) | 44155 | 304126 | 3555 | 87799 | 159367 |  |
|  |  |  | DustTrak (mg/m^3^) | 0.025 | 0.087 | 0.017 | 0.087 | 0.061 |  |
|  |  |  | DiSCmini (#cm^3^) | 37873 | 912037 | 1848 | 51598 | 456583 |  |

^a^ Using background value determined via sequential method.

^b^ Using background value determined via sampling of the same activity when masterbatch contained no NM.

^c^ Although classified as ‘unlikely’, SEM observations are noted.

# Raw data – real-time instruments


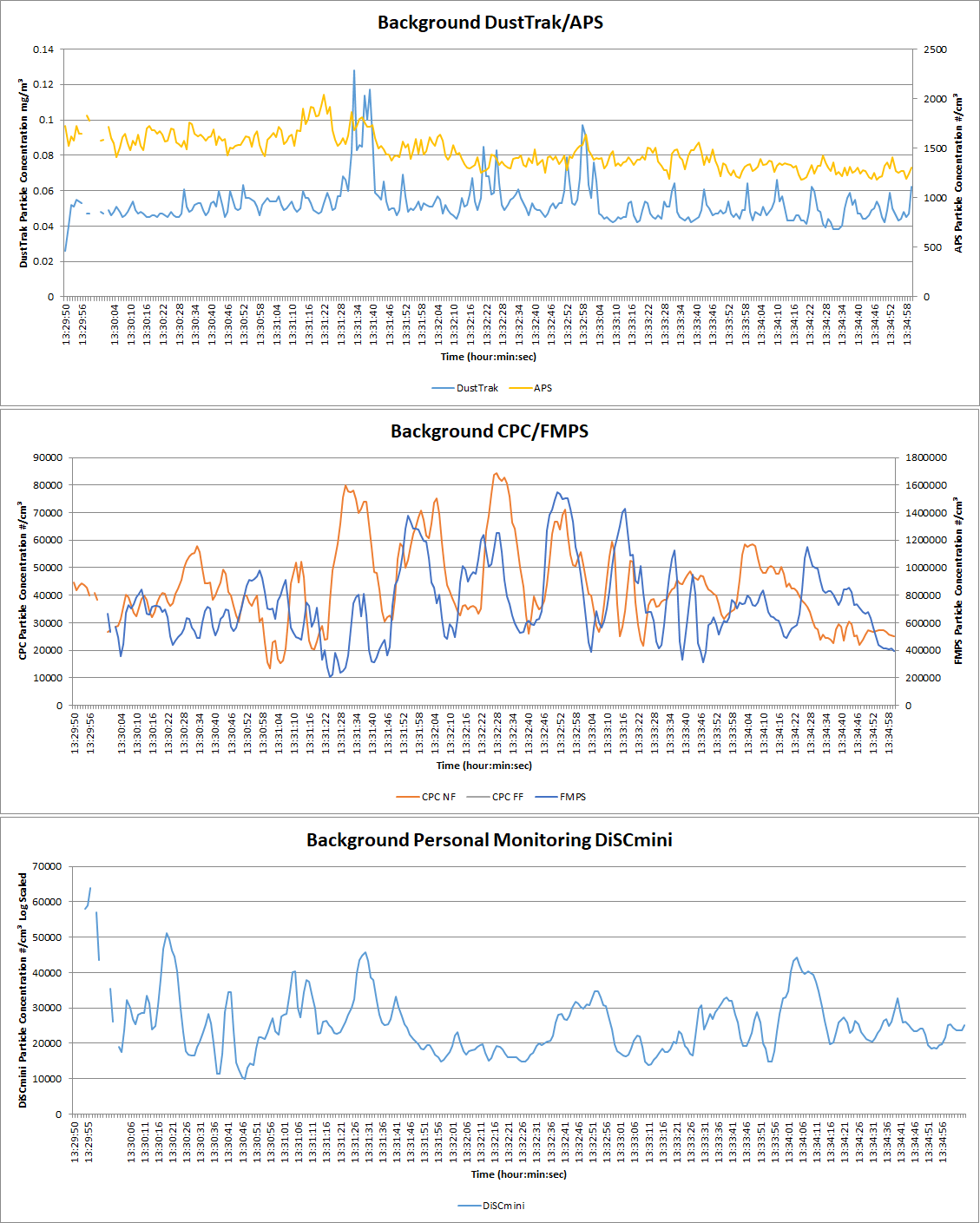


Figure S1a Time-stamped particle detection during filament production - background. Measurements performed by DustTrak, APS, FMPS, CPC and DISCmini; conducted during background measurements.


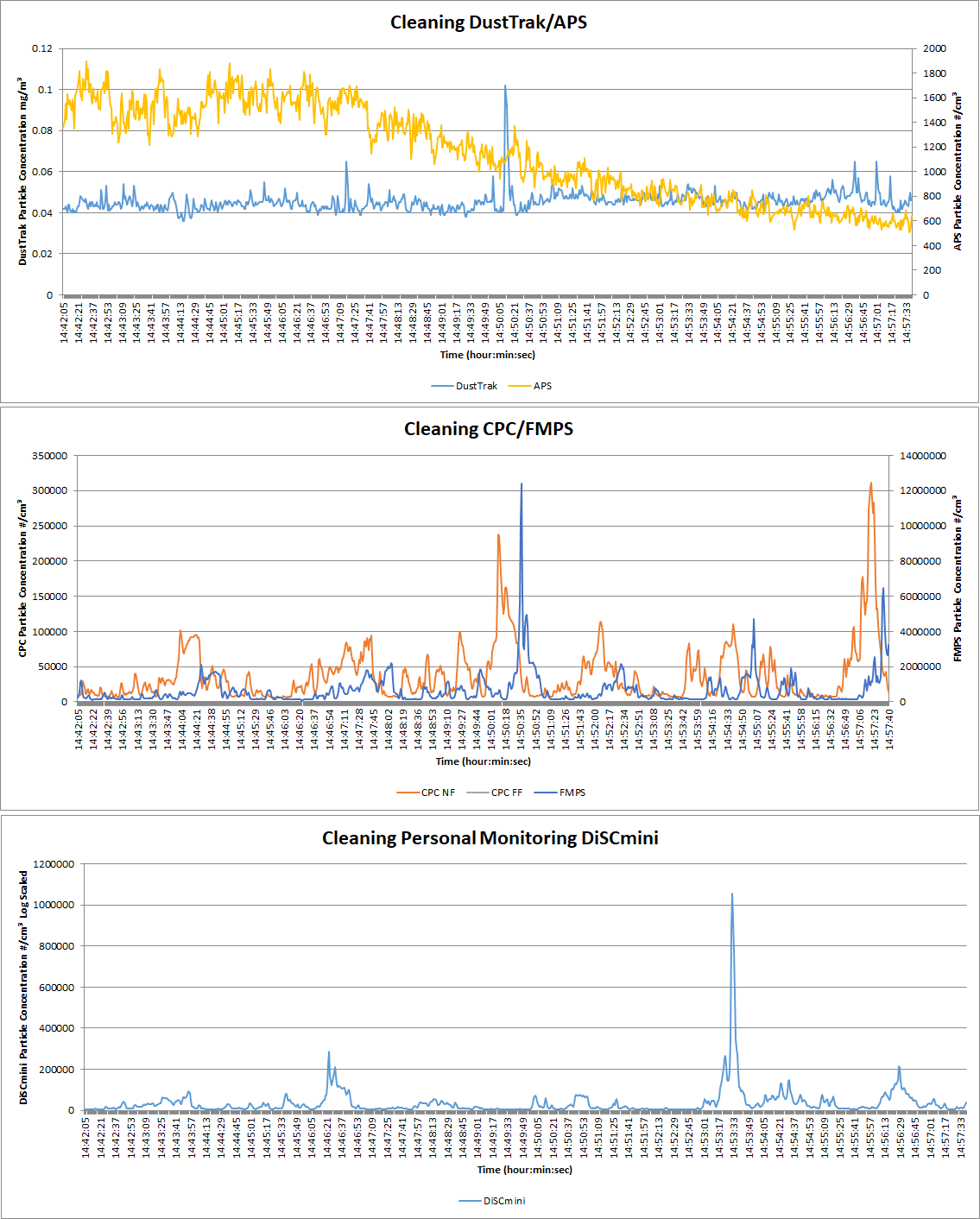


Figure S1b Time stamped particle detection during filament production - cleaning. Measurements performed by DustTrak, APS, FMPS, CPC and DISCmini; conducted during cleaning processes.


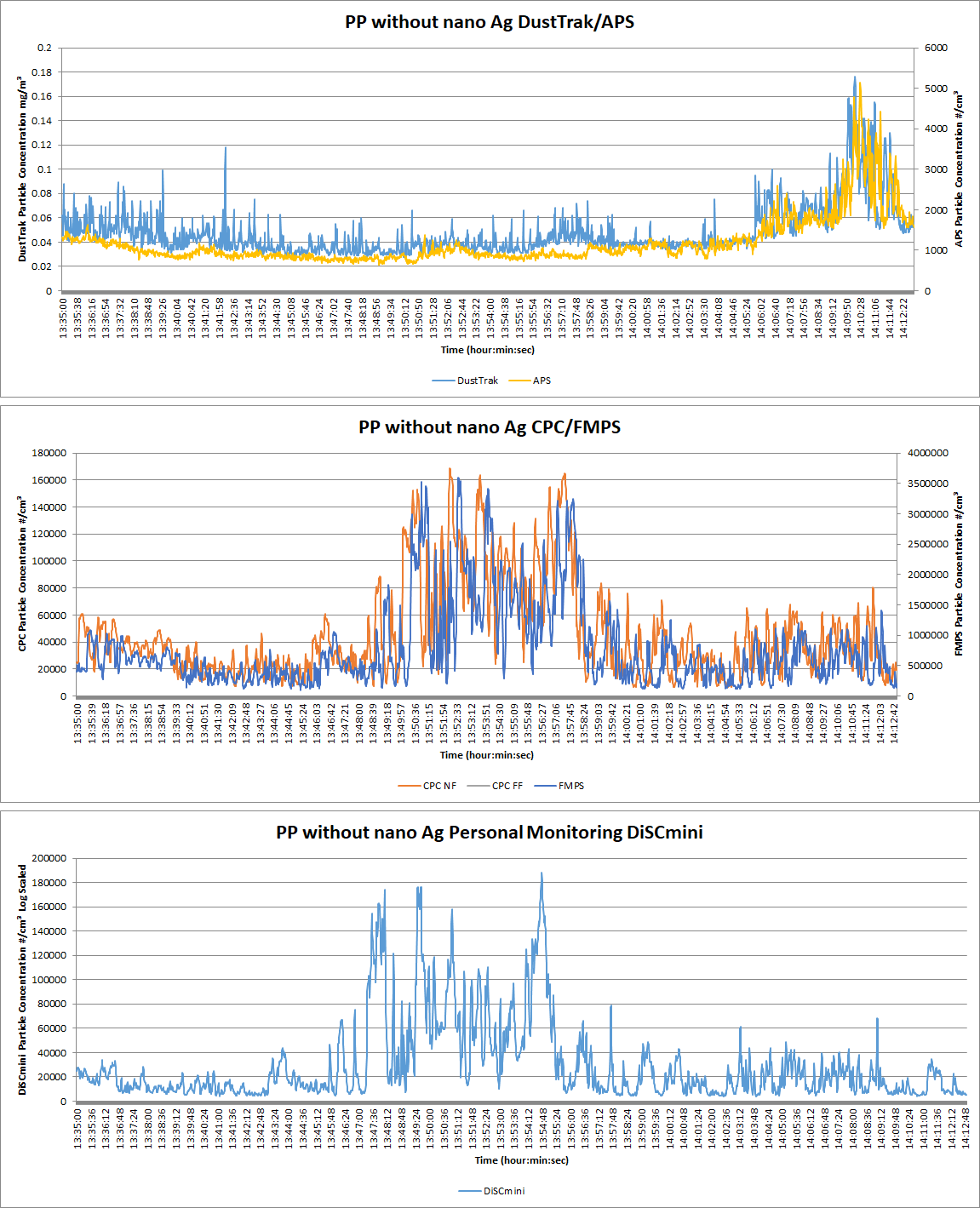


Figure S1c Time stamped particle detection during filament production – PP only. Measurements performed by DustTrak, APS, FMPS, CPC and DISCmini; conducted during filament production using PP only.


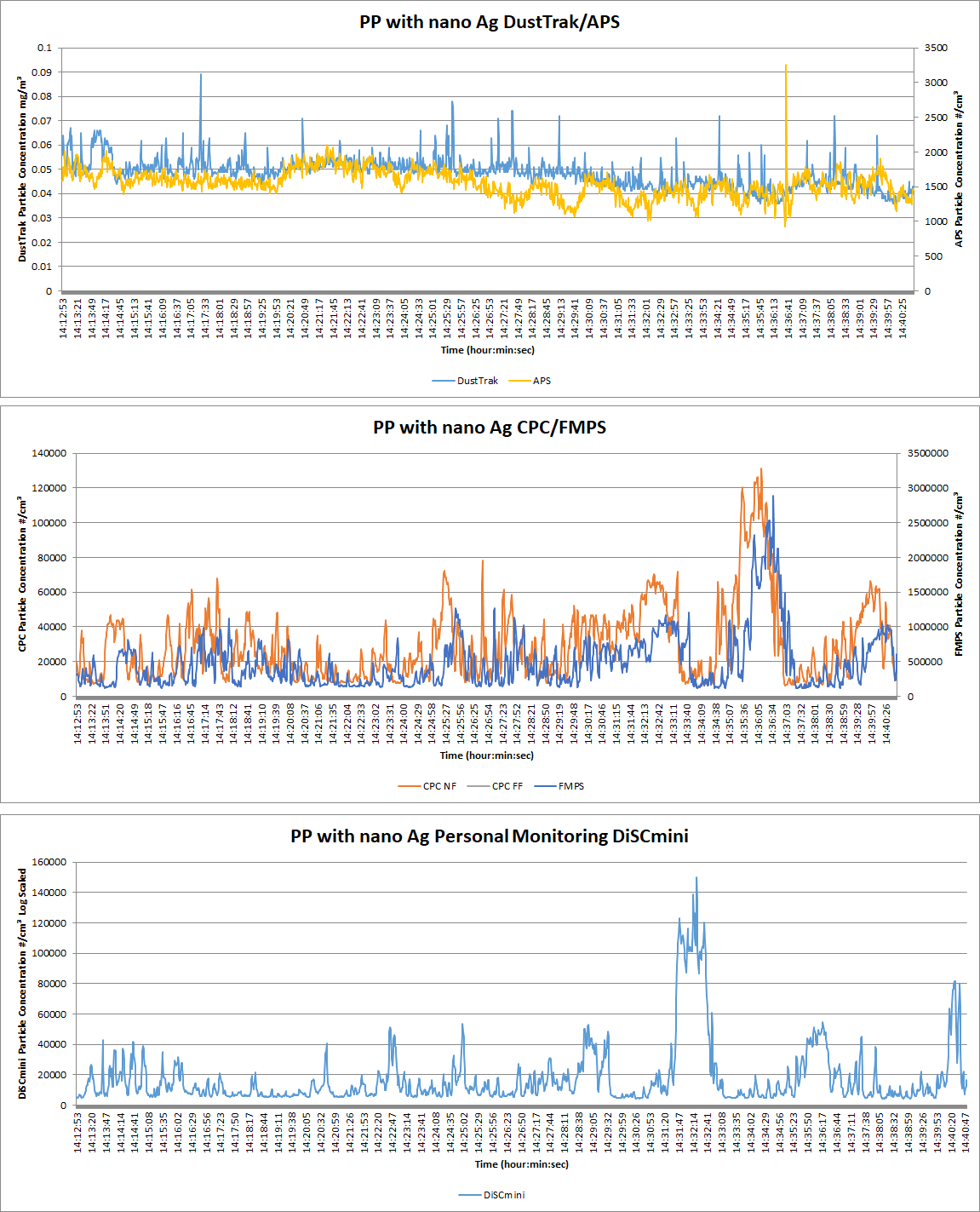


Figure S1d Time stamped particle detection during filament production – PP with nano-Ag. Measurements performed by DustTrak, APS, FMPS, CPC and DISCmini; conducted during filament production using nano-Ag with PP.


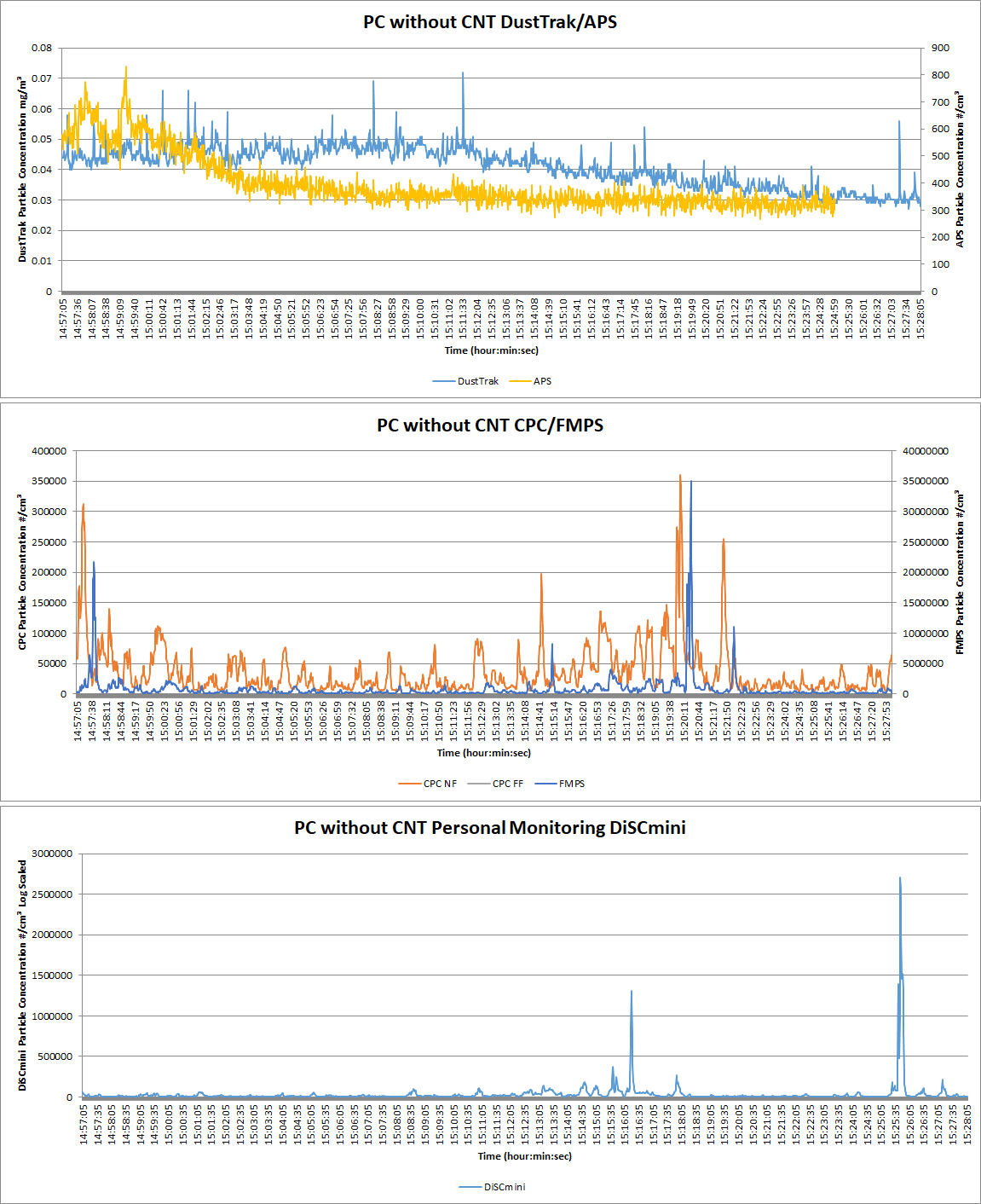


Figure S1e Time stamped particle detection during filament production – PC only. Measurements performed by DustTrak, APS, FMPS, CPC and DISCmini; conducted during filament production using PC only.


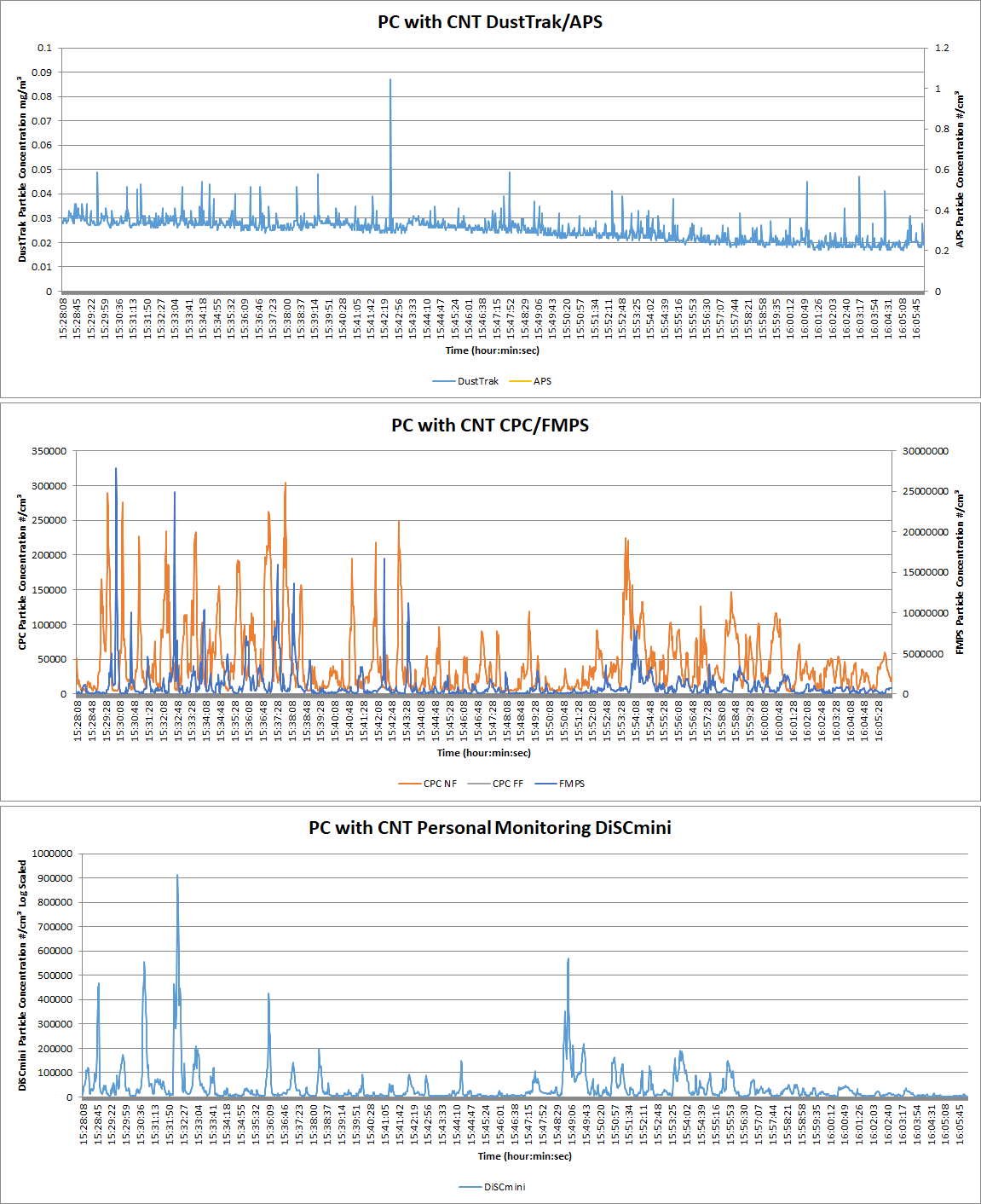


Figure S1f Time stamped particle detection during filament production – PC with SWCNTs. Measurements performed by DustTrak, APS, FMPS, CPC and DISCmini; conducted during filament production using SWCNT with PC.


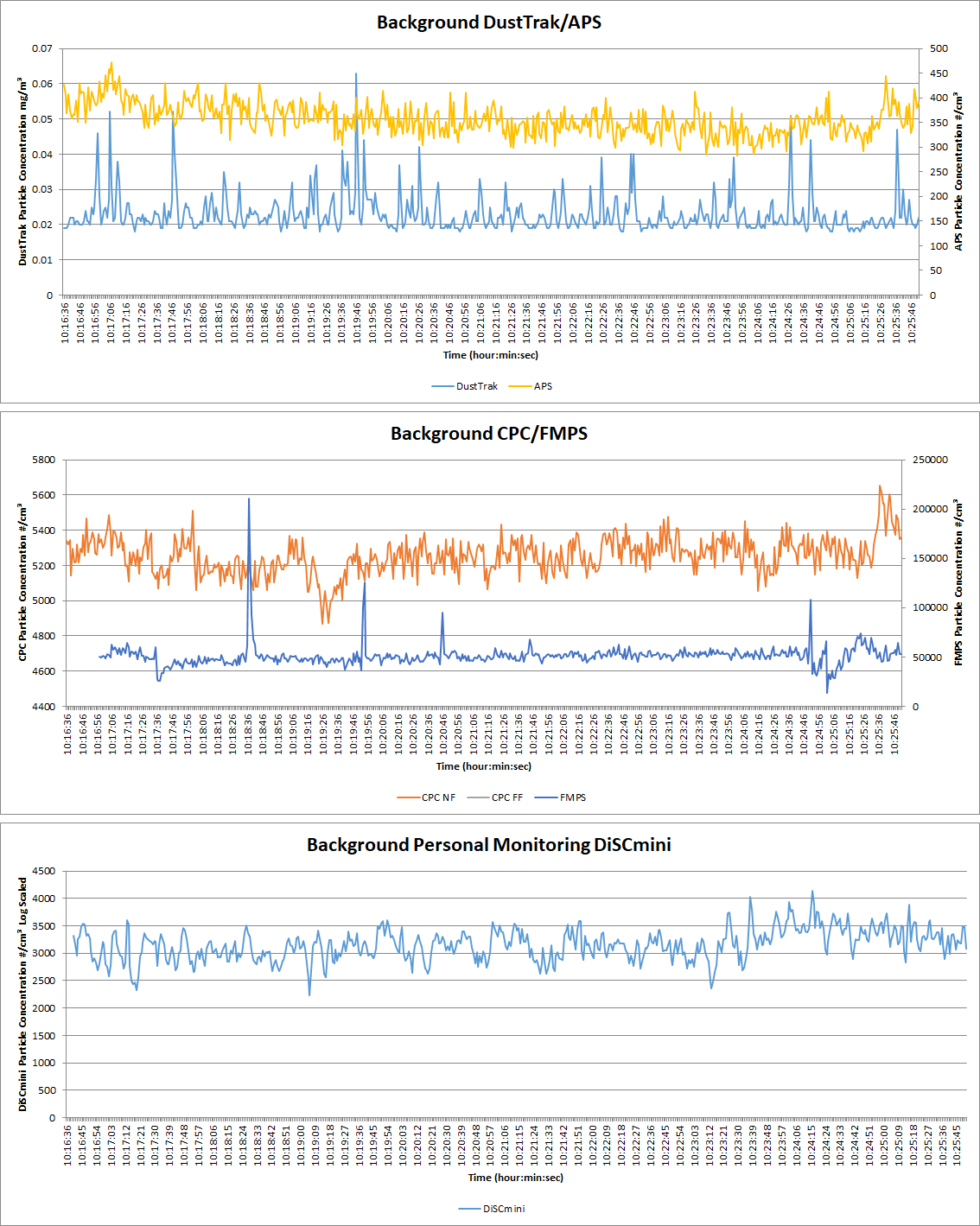


Figure S2a Time stamped particle detection during weighing – background 1. Measurements performed by DustTrak, APS, FMPS, CPC and DISCmini, conducted during weighing; includes background measurements before activity.


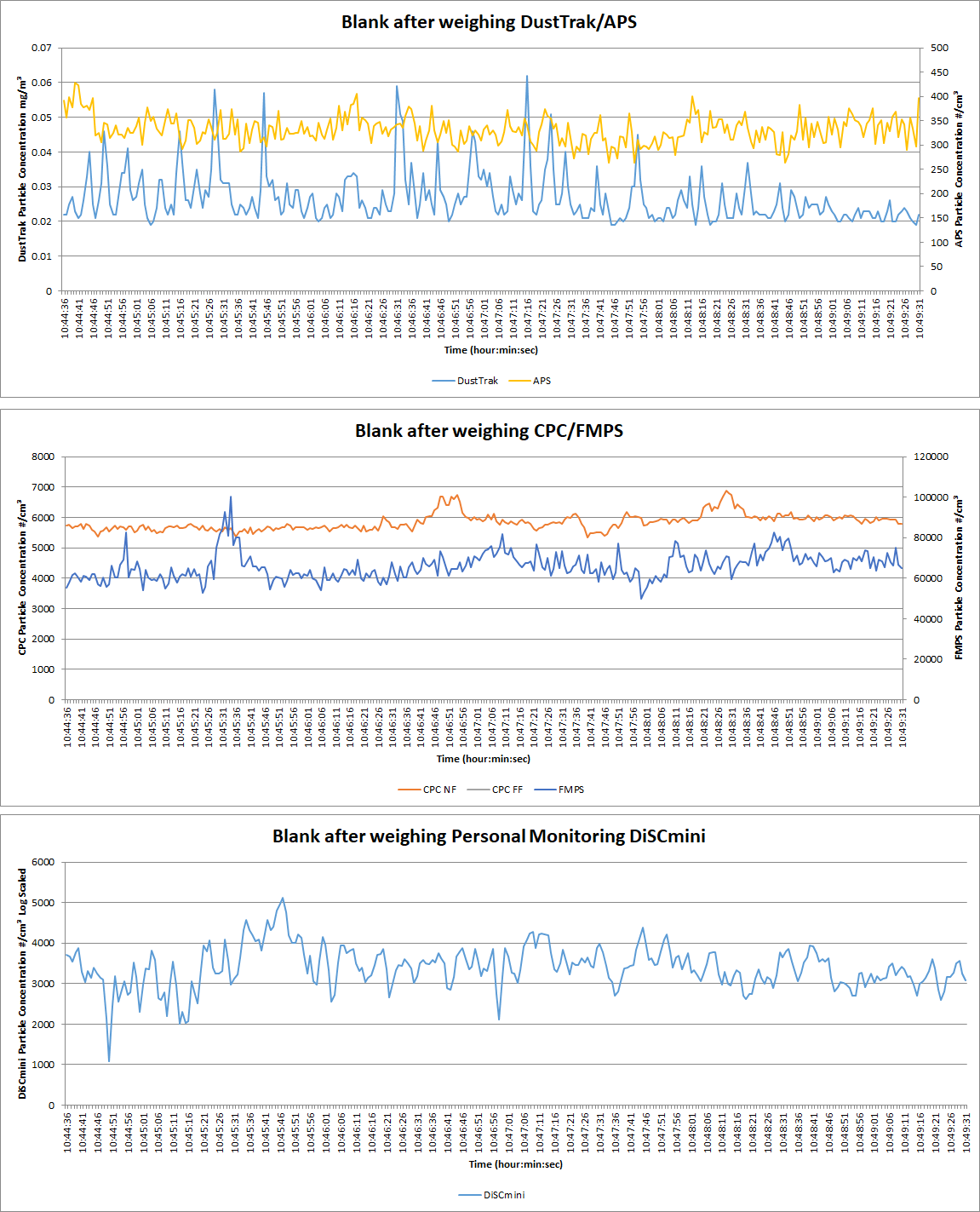


Figure S2b Time stamped particle detection during weighing – background 2. Measurements performed by DustTrak, APS, FMPS, CPC and DISCmini, conducted during weighing; includes background measurements, after activity.


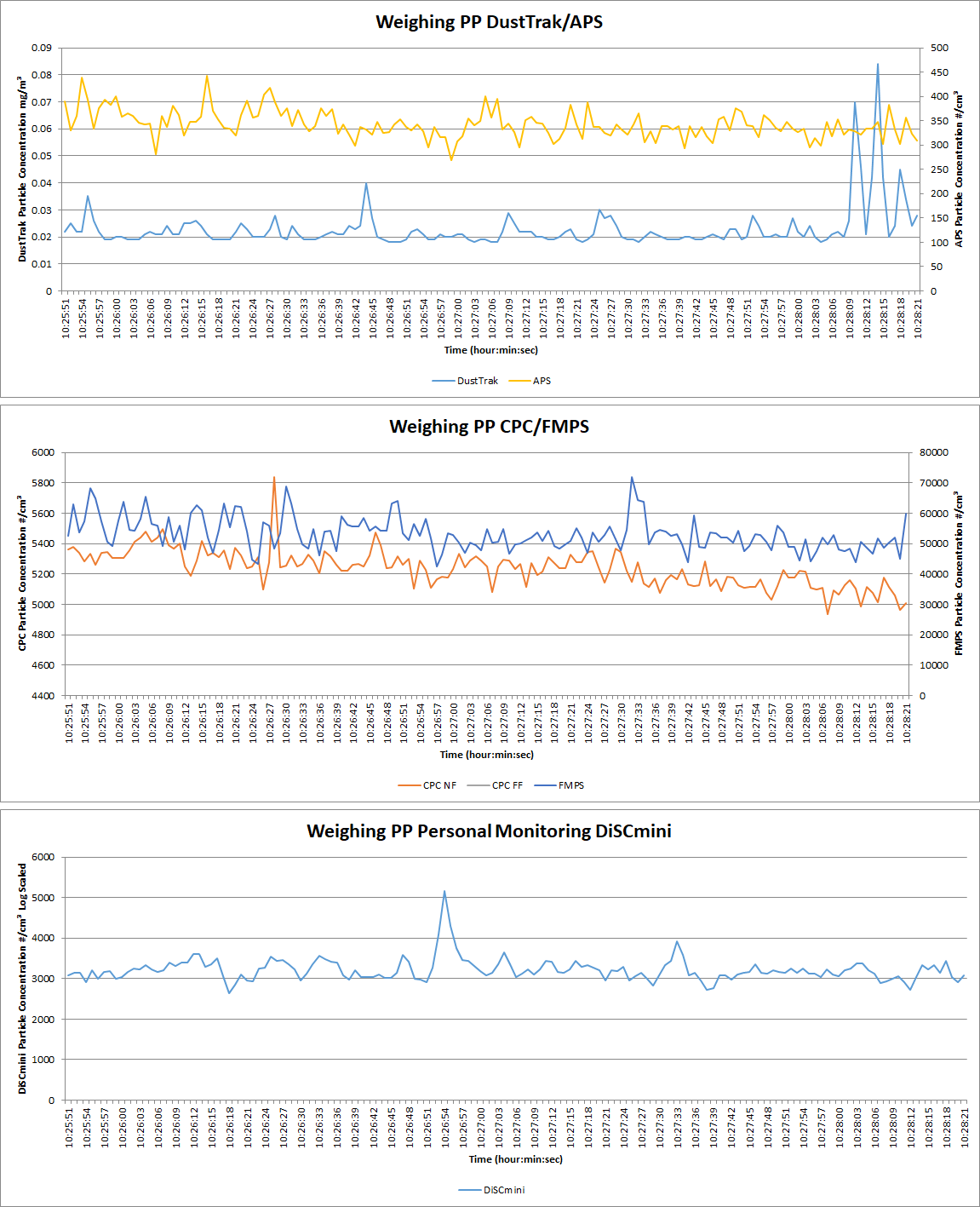


Figure S2c Time stamped particle detection during weighing – PP only. Measurements performed by DustTrak, APS, FMPS, CPC and DISCmini, conducted during weighing; includes weighing of PP only.


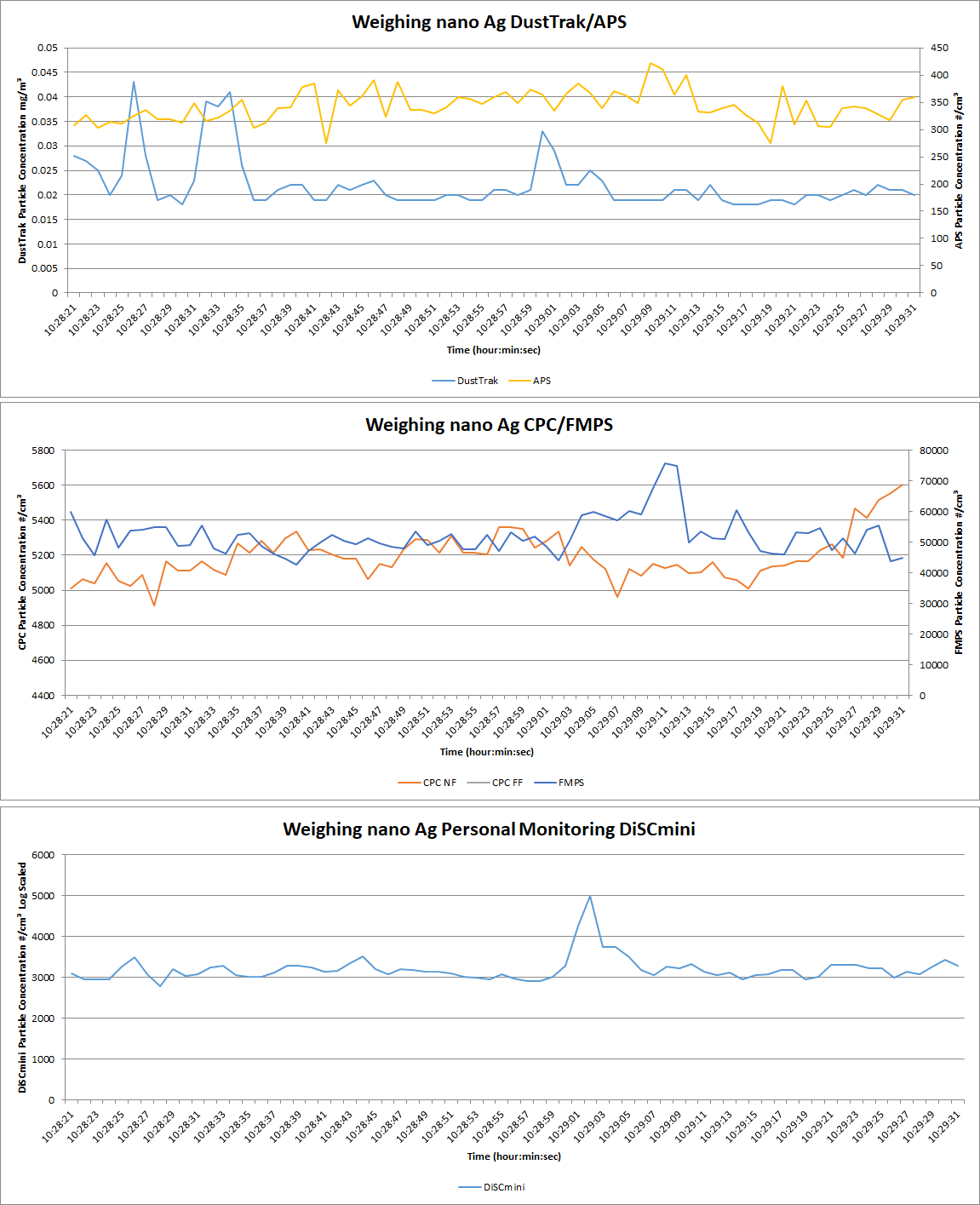


Figure S2d Time stamped particle detection during weighing – PP with nano-Ag. Measurements performed by DustTrak, APS, FMPS, CPC and DISCmini, conducted during weighing; includes weighing of PP with nano-Ag.


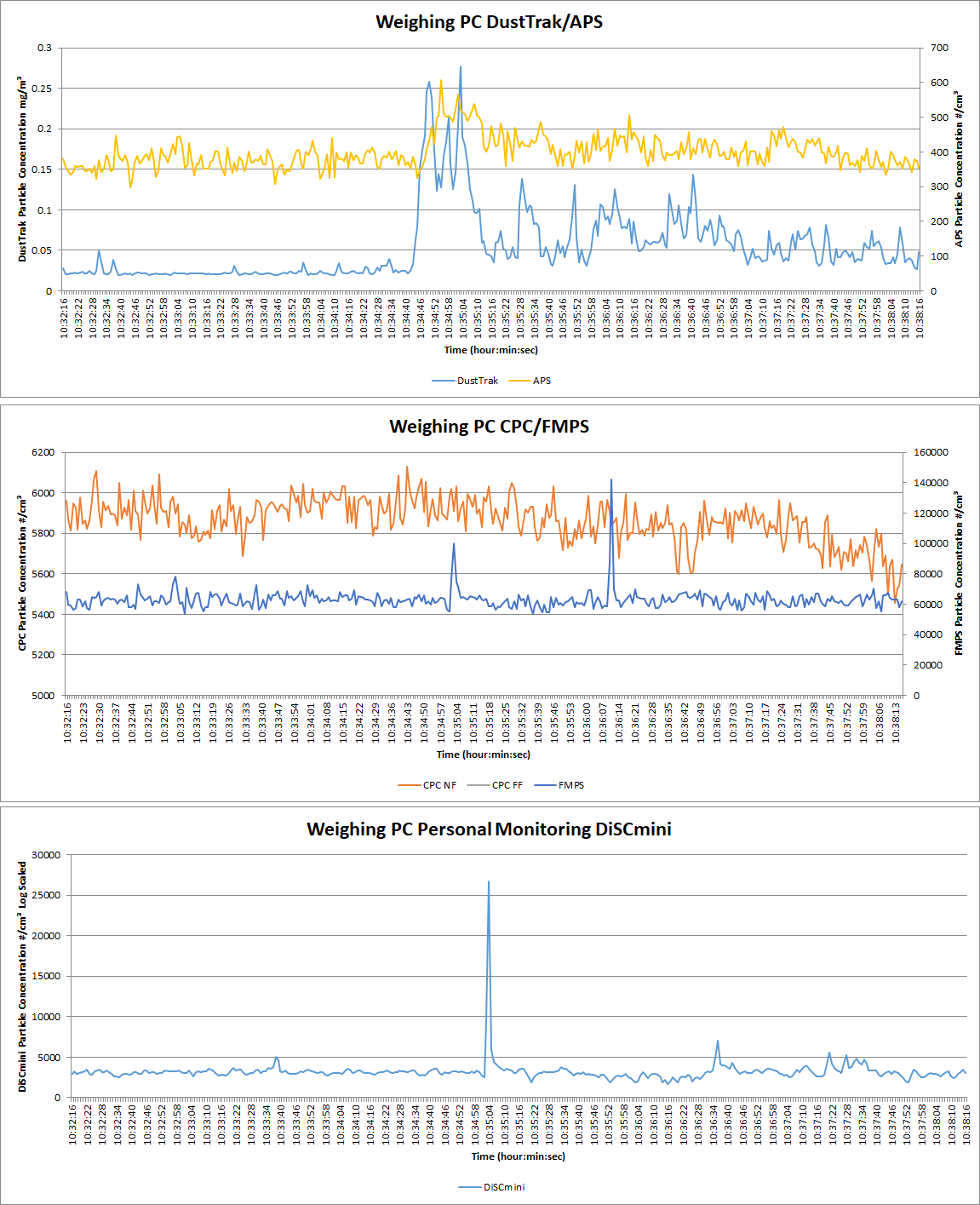


Figure S2e Time stamped particle detection during weighing – PC only. Measurements performed by DustTrak, APS, FMPS, CPC and DISCmini, conducted during weighing; includes weighing of PC only.


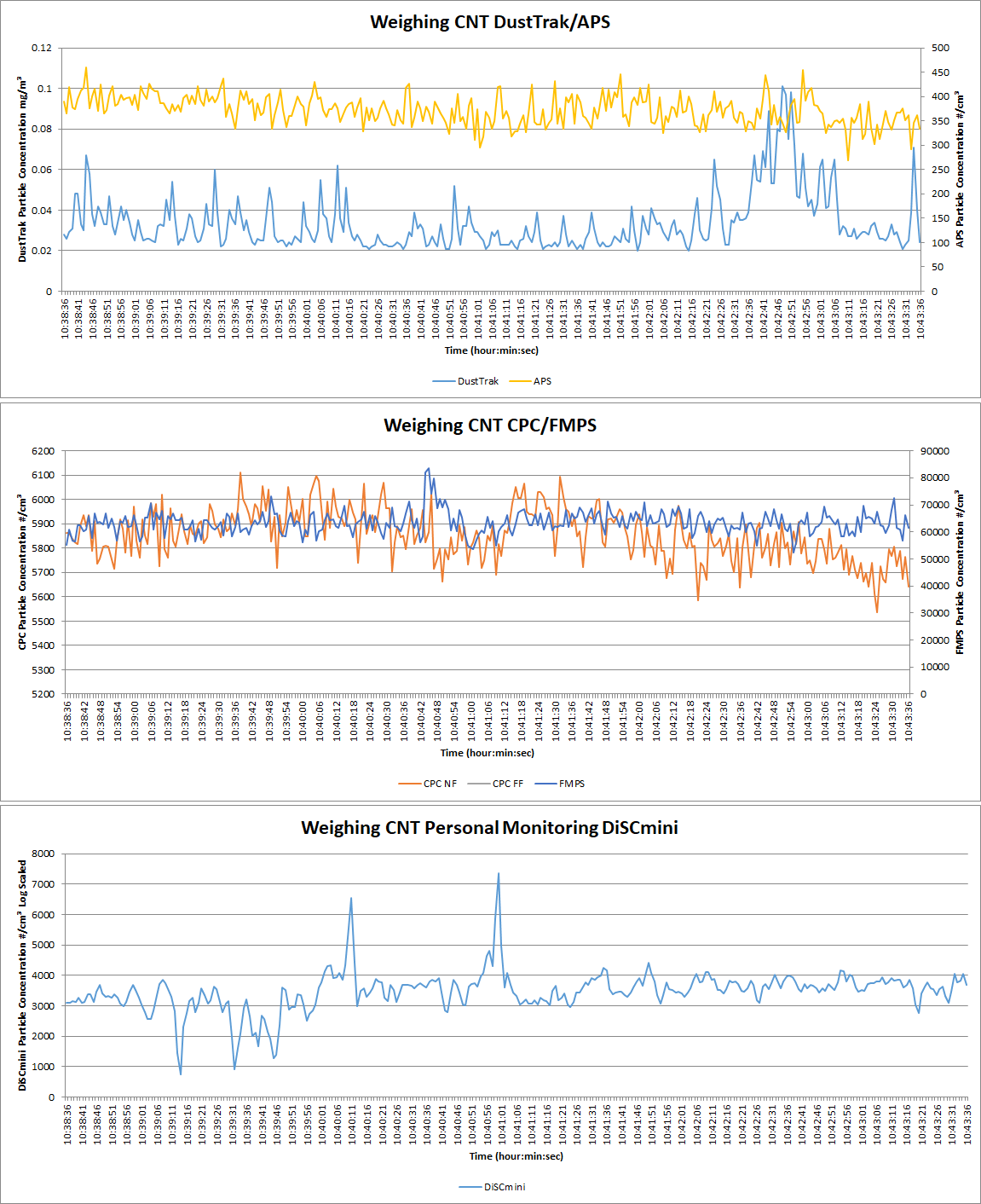


Figure S2f Time stamped particle detection during weighing – PC with SWCNTs. Measurements performed by DustTrak, APS, FMPS, CPC and DISCmini, conducted during weighing; includes weighing of PC with SWCNTs.


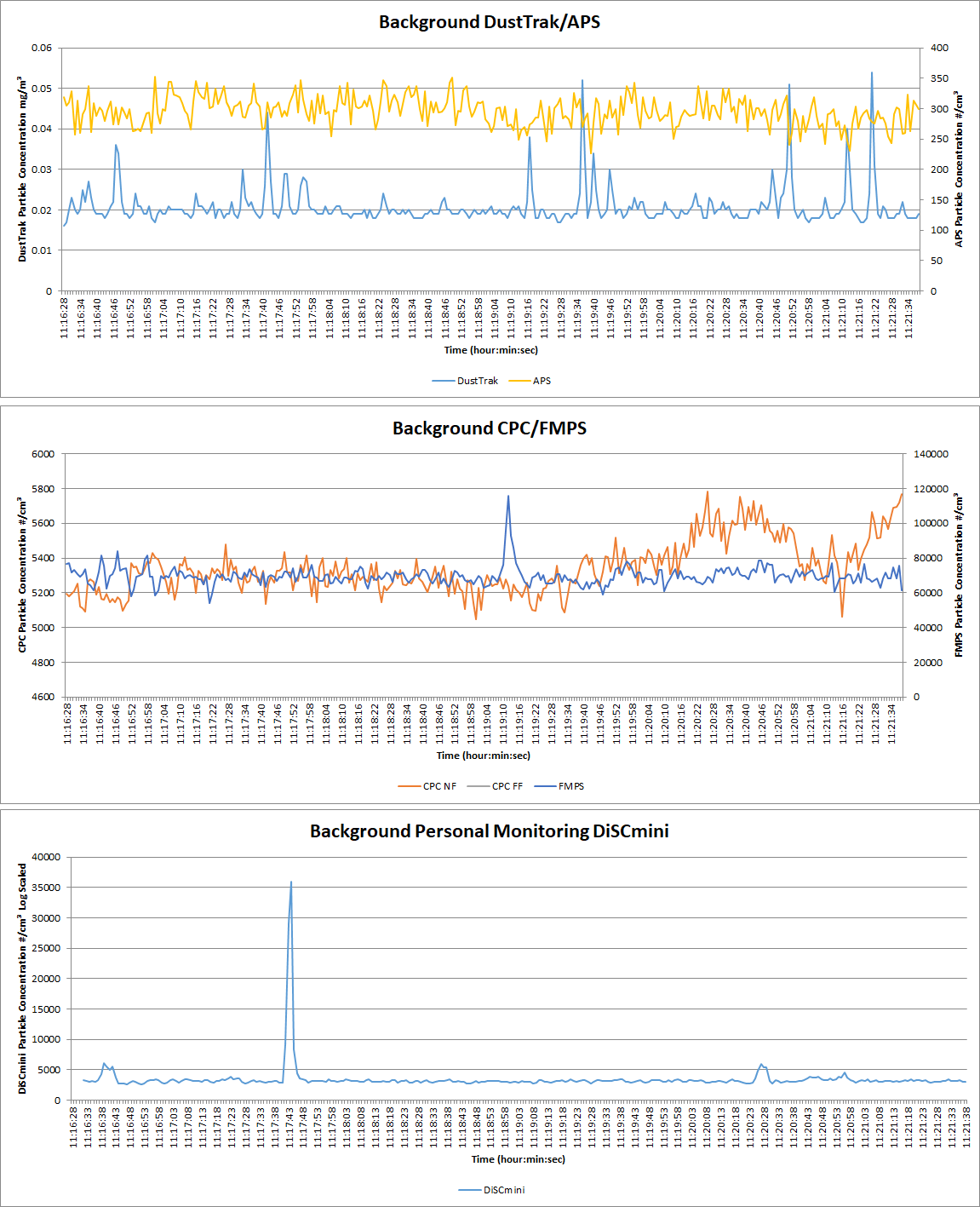


Figure S3a Time stamped particle detection during mixing – background 1. Measurements performed by DustTrak, APS, FMPS, CPC and DISCmini, conducted during mixing; includes background measurements before activity.


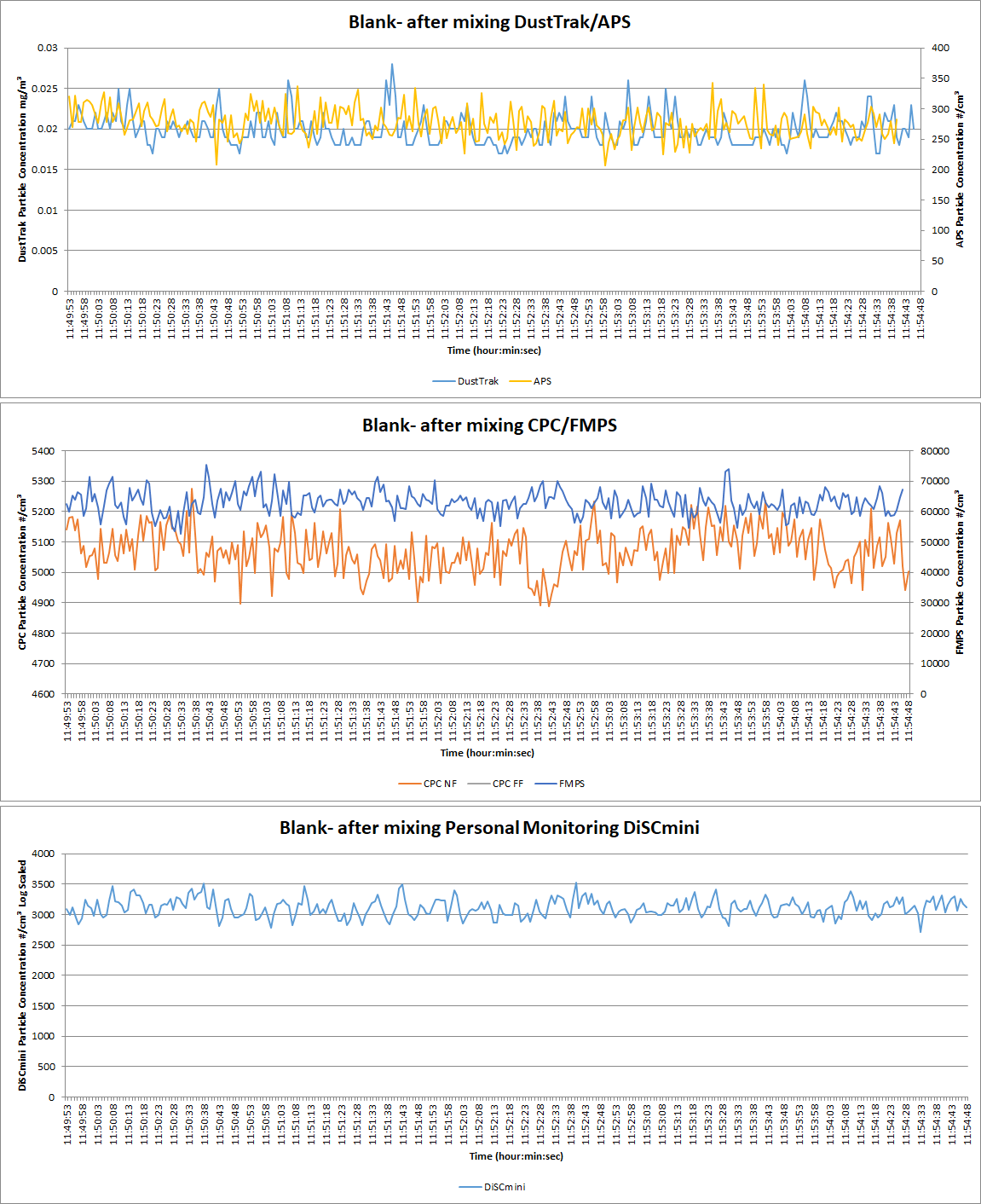


Figure S3a Time stamped particle detection during mixing – background 2. Measurements performed by DustTrak, APS, FMPS, CPC and DISCmini, conducted during mixing; includes background measurements after activity.


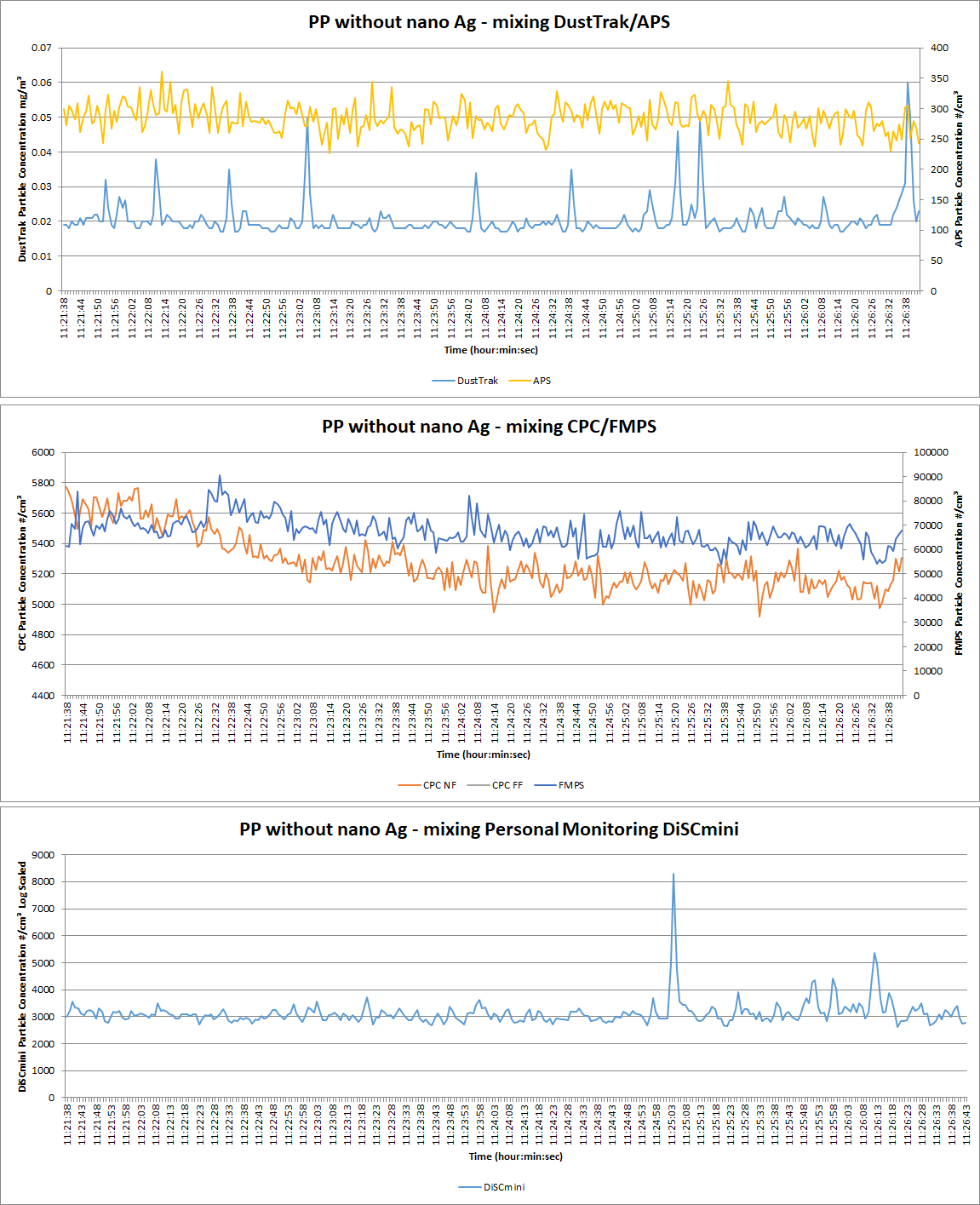


Figure S3a Time stamped particle detection during mixing – PP only. Measurements performed by DustTrak, APS, FMPS, CPC and DISCmini, conducted during mixing; includes mixing of PP only.


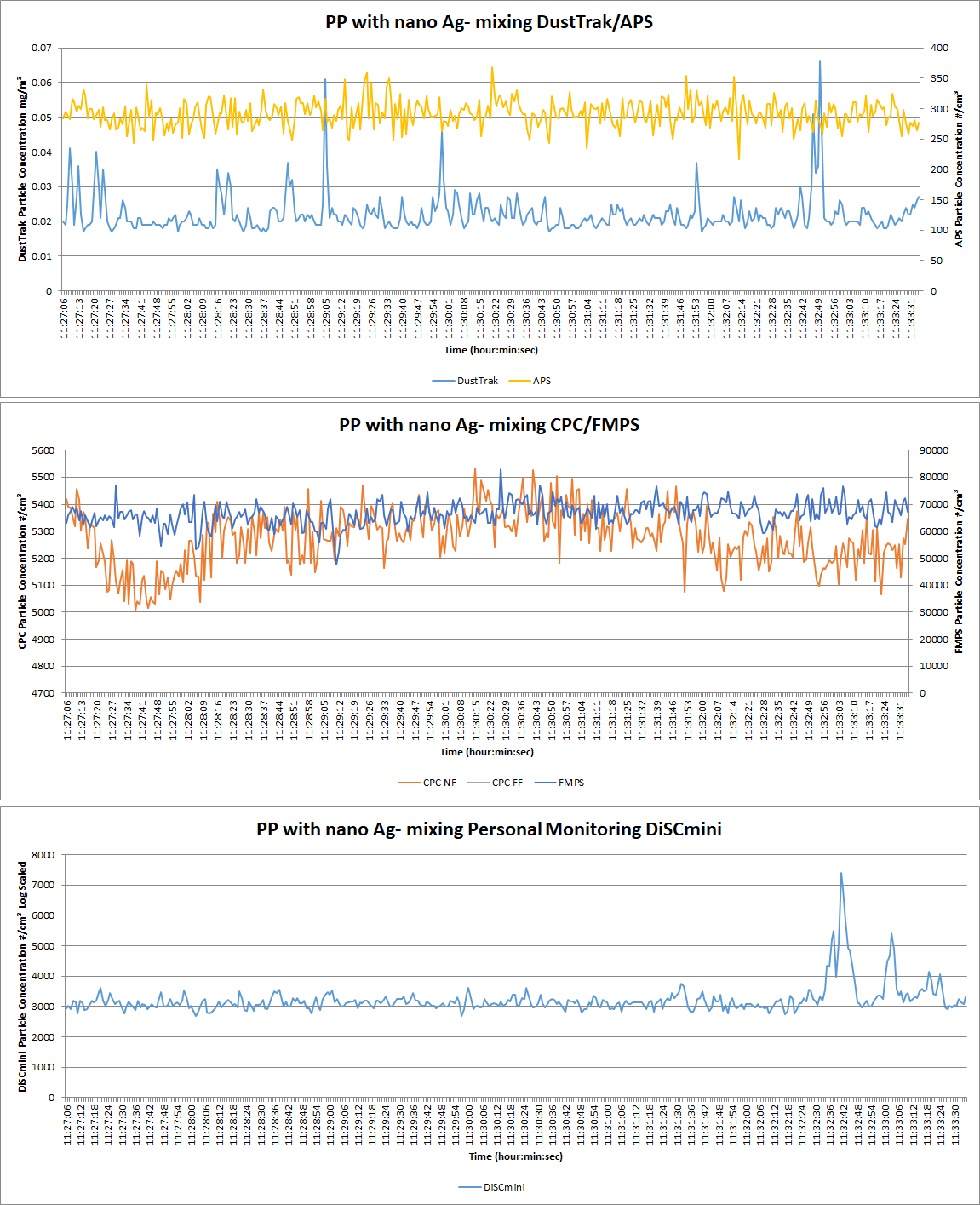


Figure S3a Time stamped particle detection during mixing – PP with nano-Ag. Measurements performed by DustTrak, APS, FMPS, CPC and DISCmini, conducted during mixing; includes mixing of PP with nano-Ag.


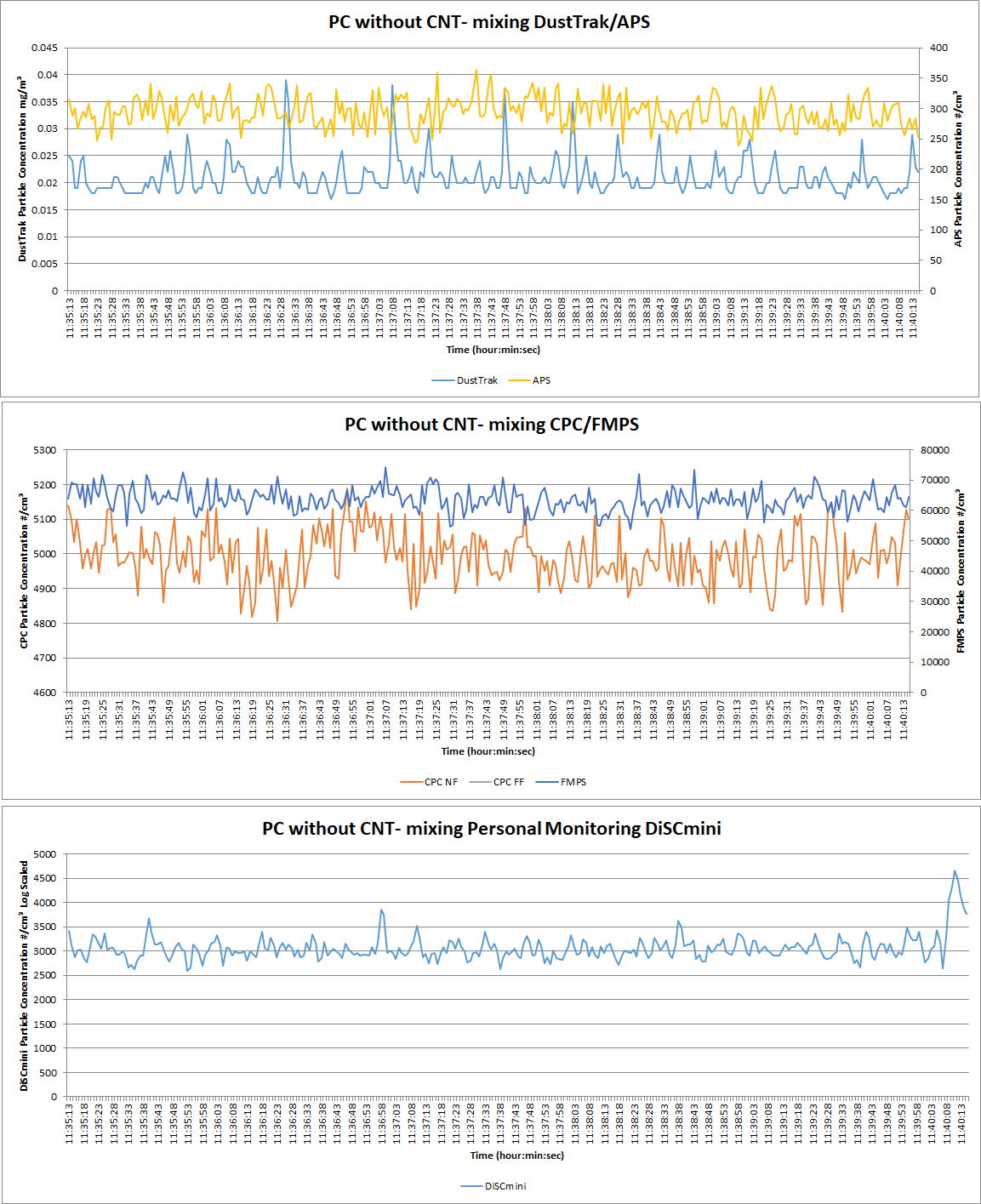


Figure S3a Time stamped particle detection during mixing – PC only. Measurements performed by DustTrak, APS, FMPS, CPC and DISCmini, conducted during mixing; includes PC only.


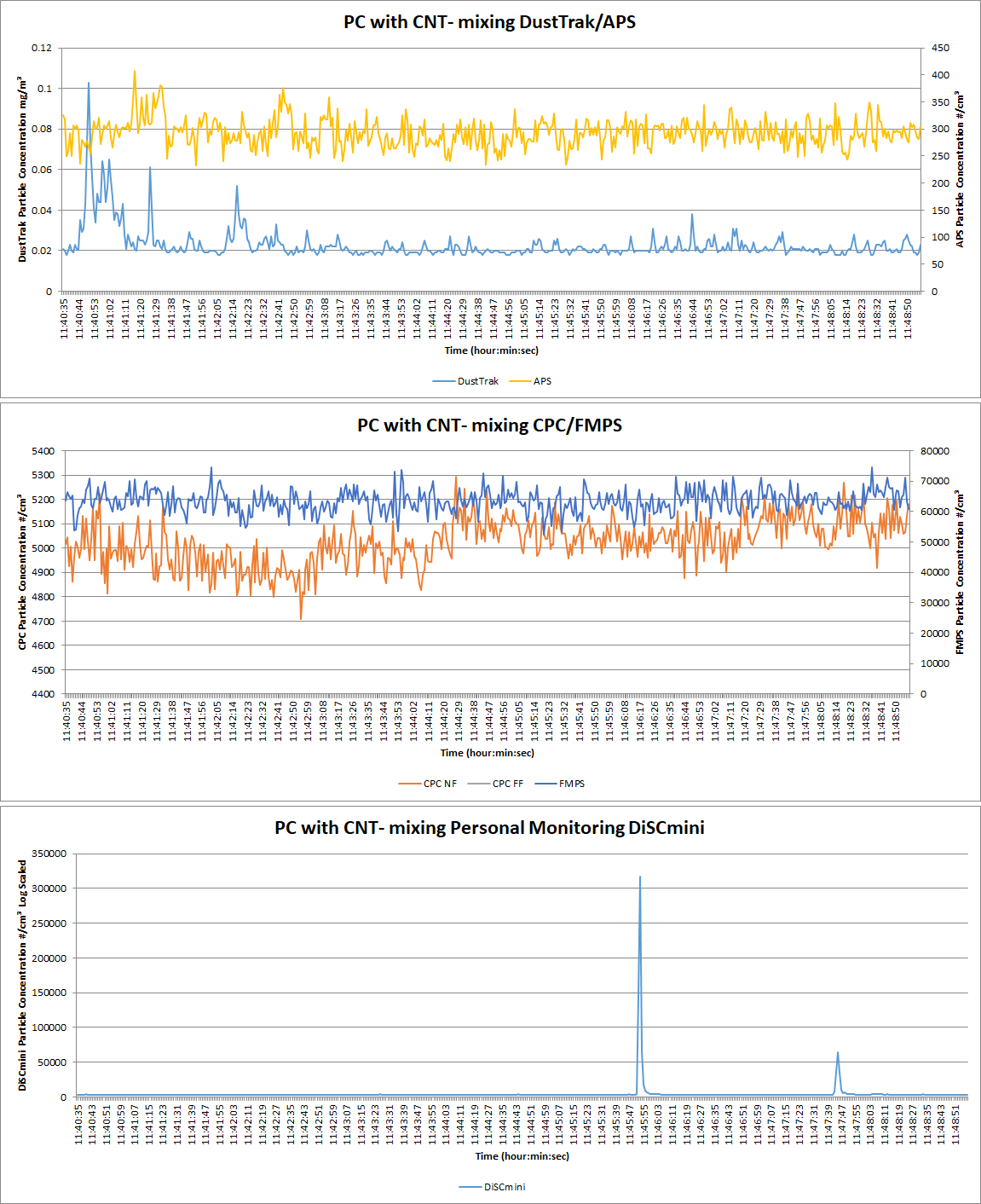


Figure S3a Time stamped particle detection during mixing – PC with SWCNTs. Measurements performed by DustTrak, APS, FMPS, CPC and DISCmini, conducted during mixing; includes PC with SWCNTs.


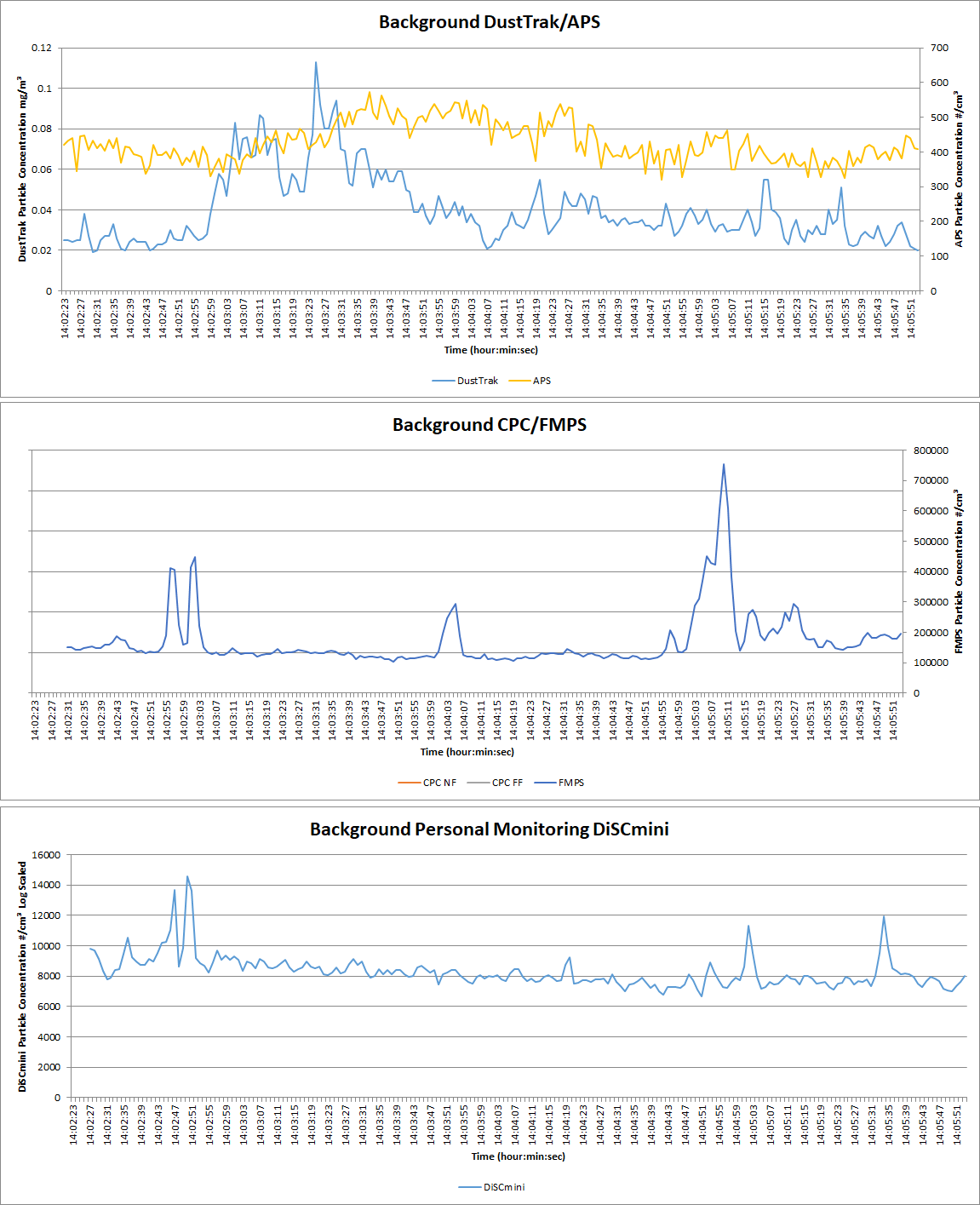


Figure S4a Time stamped particle detection during extrusion – background PP filament. Measurements performed by DustTrak, APS, FMPS and DISCmini, conducted during extrusion; includes background measurements on day of PP filament extrusion.


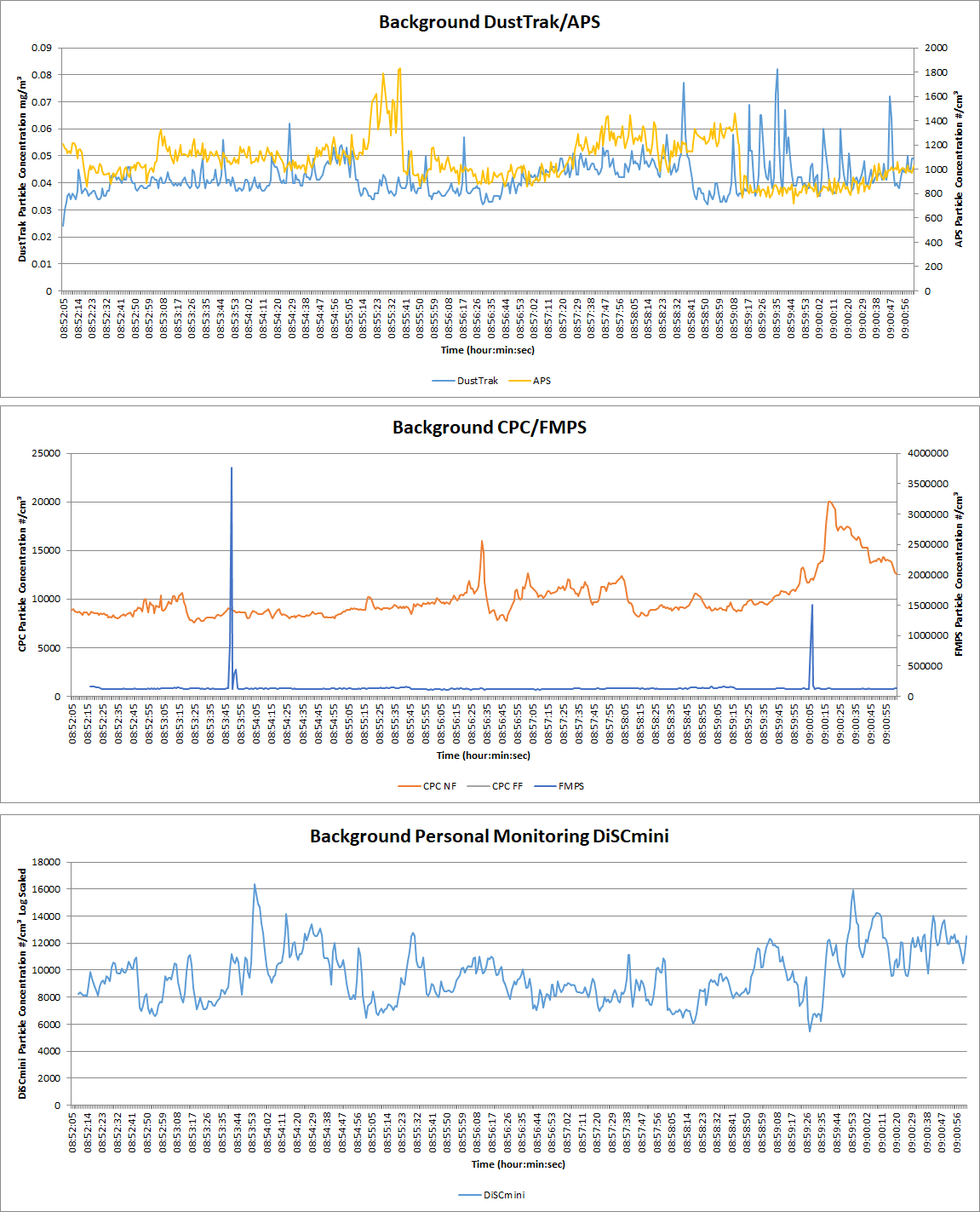


Figure S4b Time stamped particle detection during extrusion – background PC filament. Measurements performed by DustTrak, APS, FMPS, CPC and DISCmini, conducted during extrusion; includes background measurements on day of PC filament extrusion.


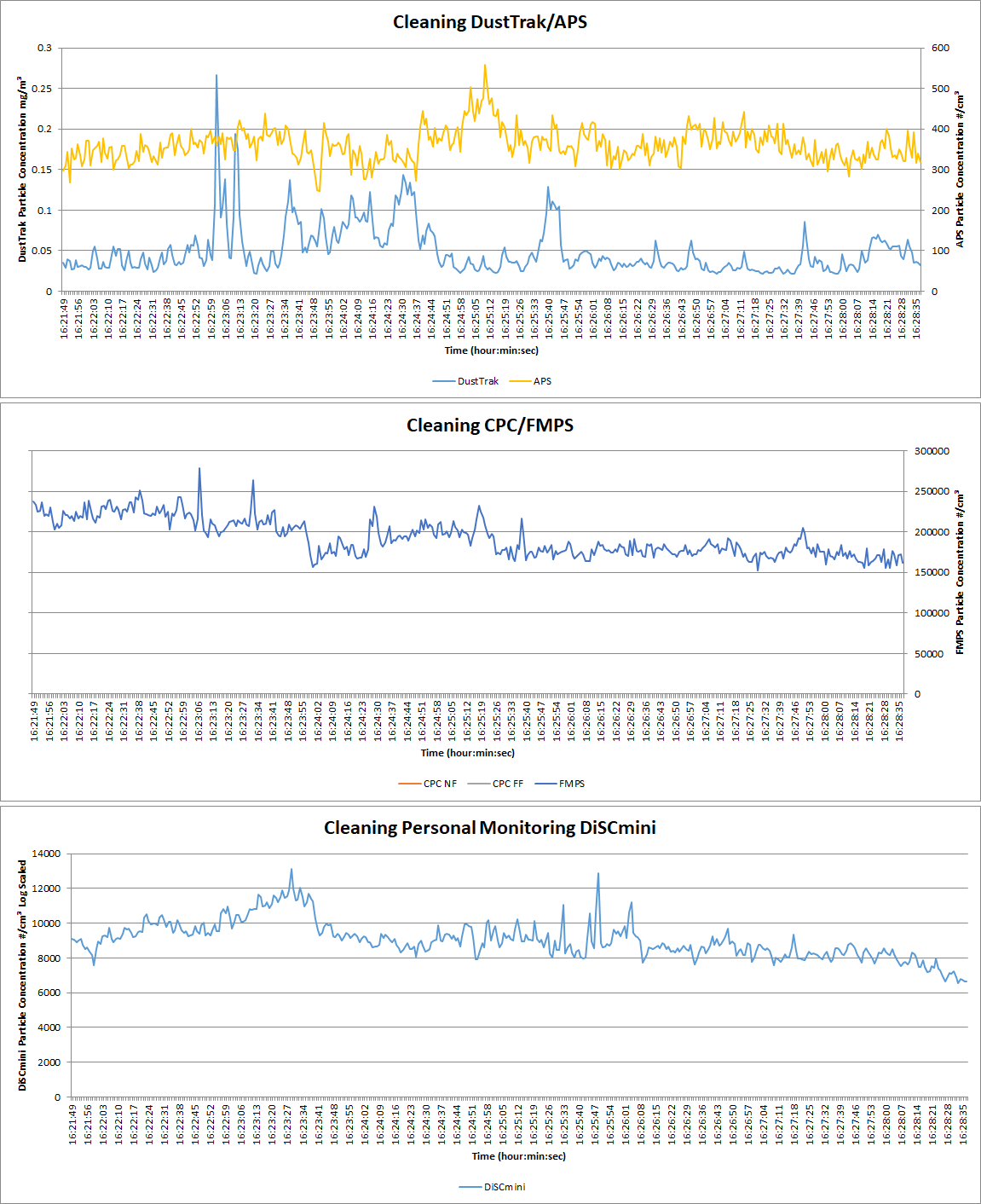


Figure S4c Time stamped particle detection during extrusion – cleaning PP filament. Measurements performed by DustTrak, APS, FMPS, CPC and DISCmini, conducted during extrusion; includes cleaning on day of PP filament extrusion.


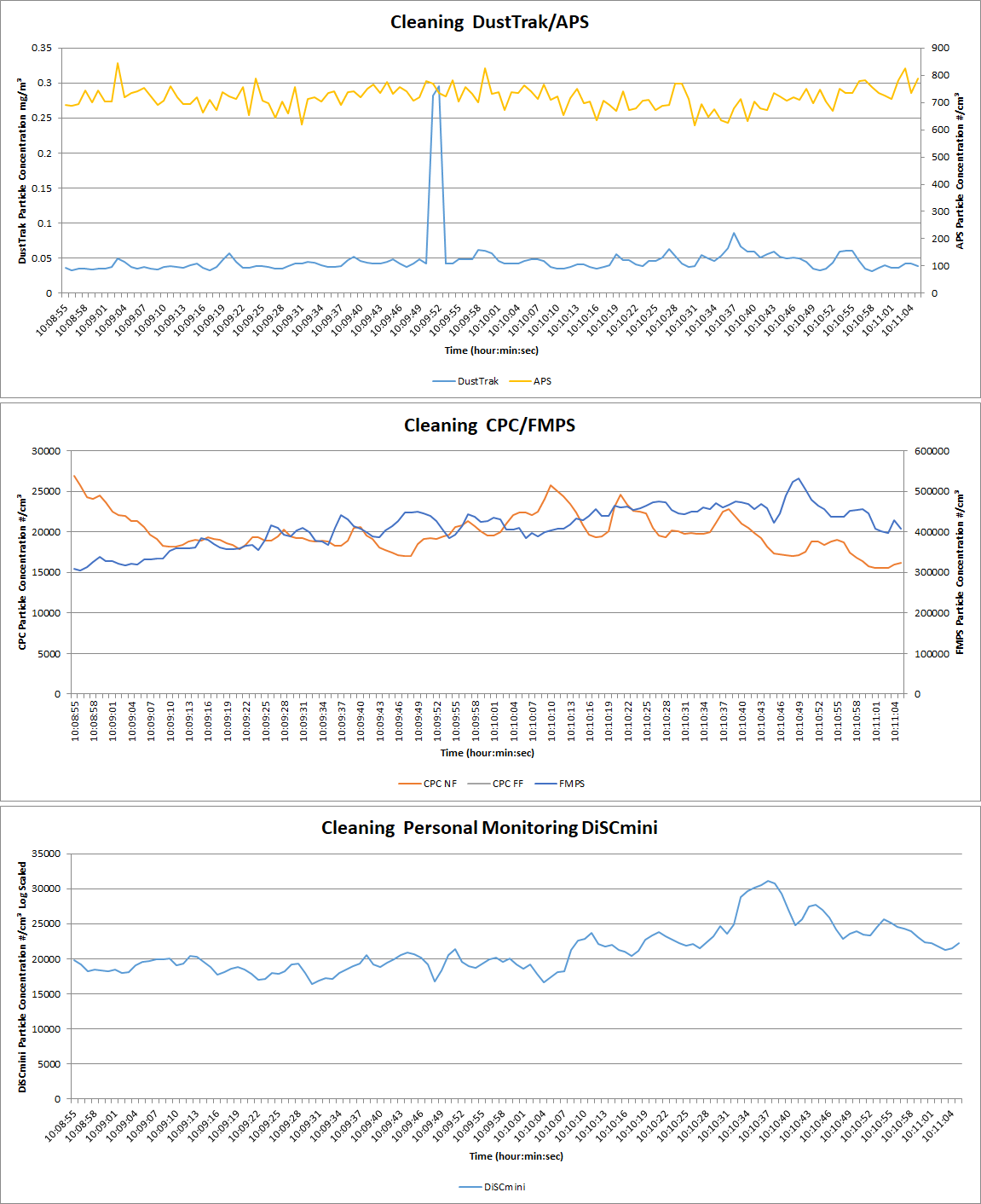


Figure S4d Time stamped particle detection during extrusion – cleaning PC filament. Measurements performed by DustTrak, APS, FMPS, CPC and DISCmini, conducted during extrusion; includes cleaning on day of PC filament extrusion.


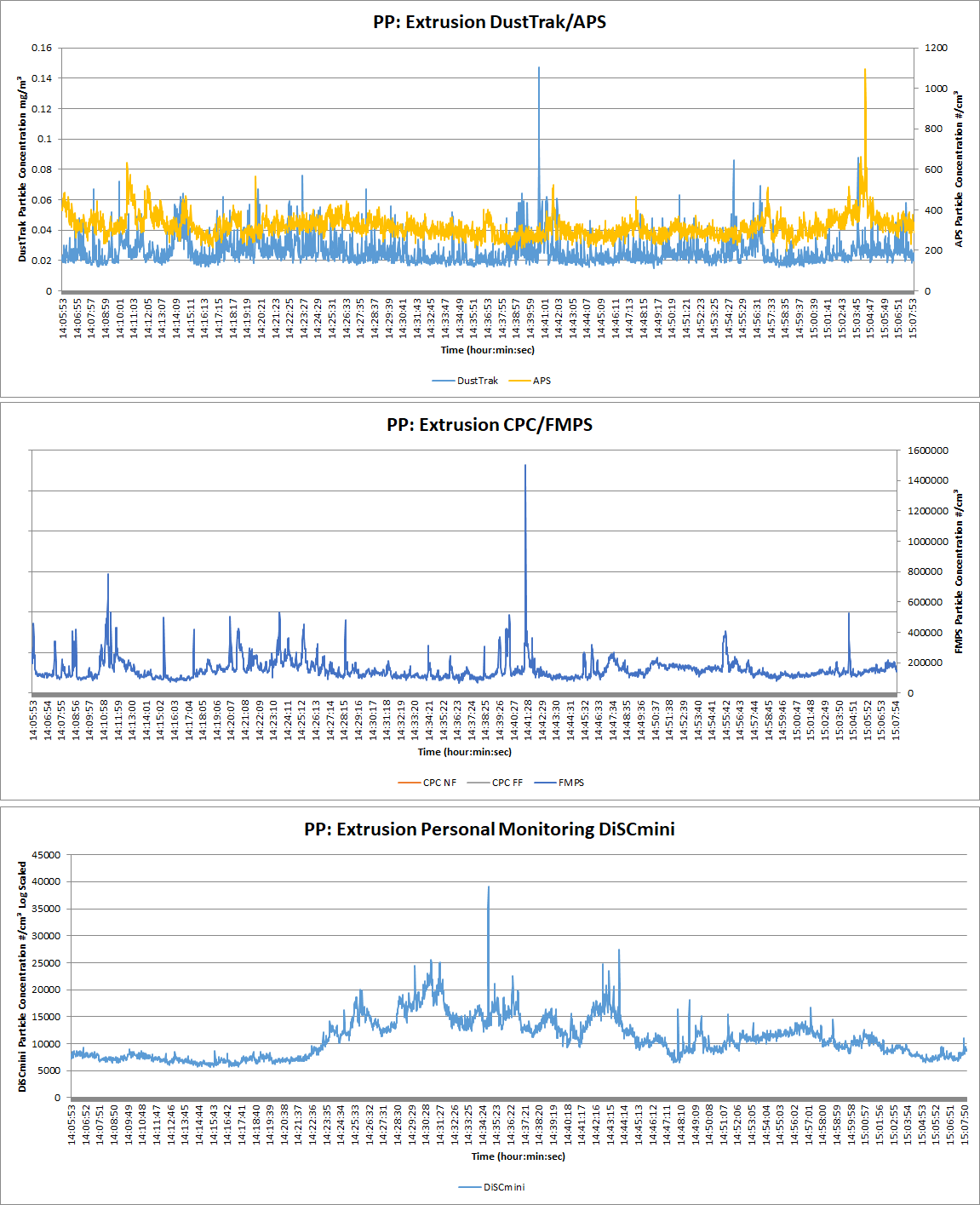


Figure S4e Time stamped particle detection during extrusion – PP only. Measurements performed by DustTrak, APS, FMPS, and DISCmini, conducted during extrusion; includes extrusion of PP only.


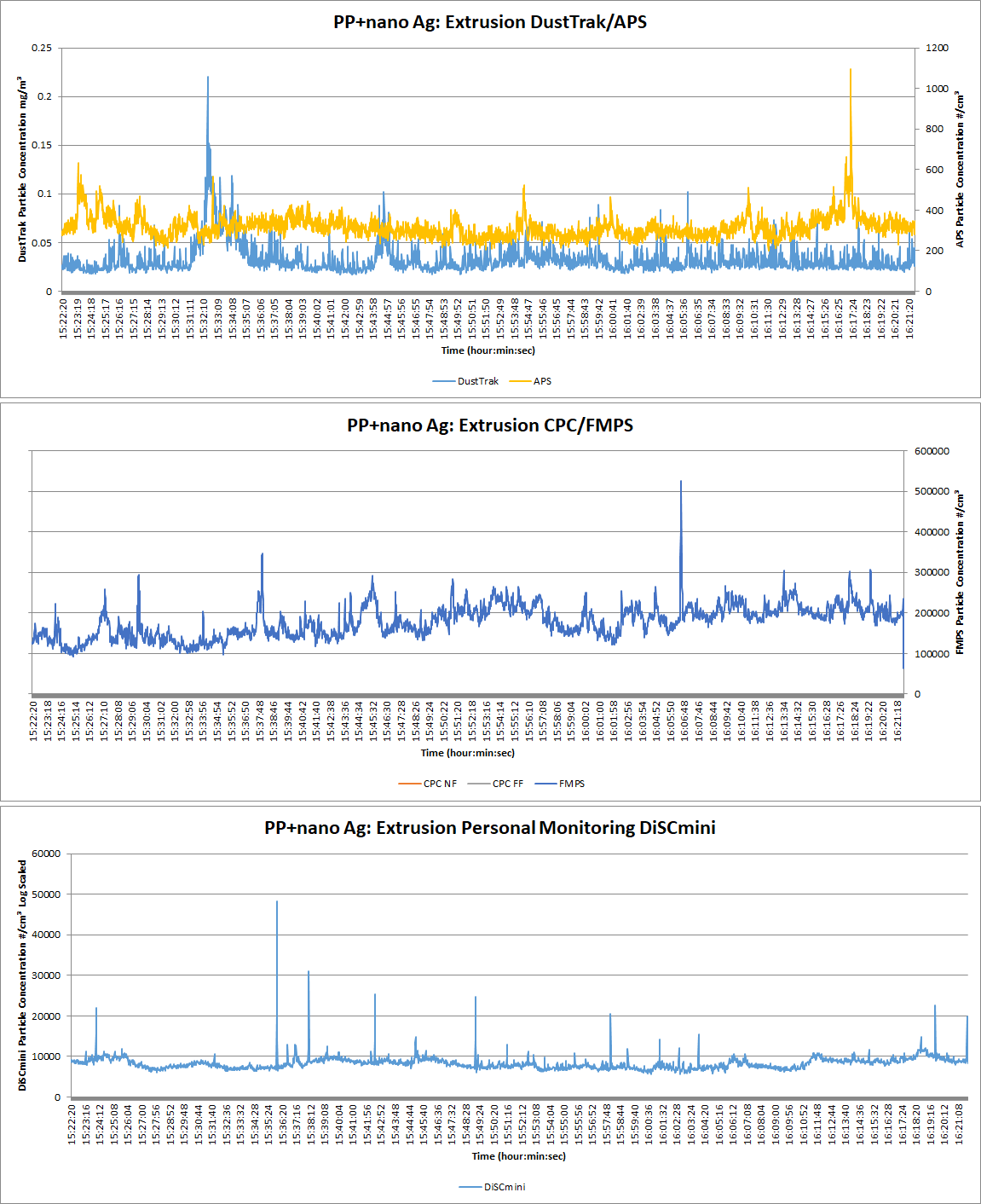


Figure S4f Time stamped particle detection during extrusion – PP with nano-Ag. Measurements performed by DustTrak, APS, FMPS, and DISCmini, conducted during extrusion; includes extrusion of PP with nano-Ag.


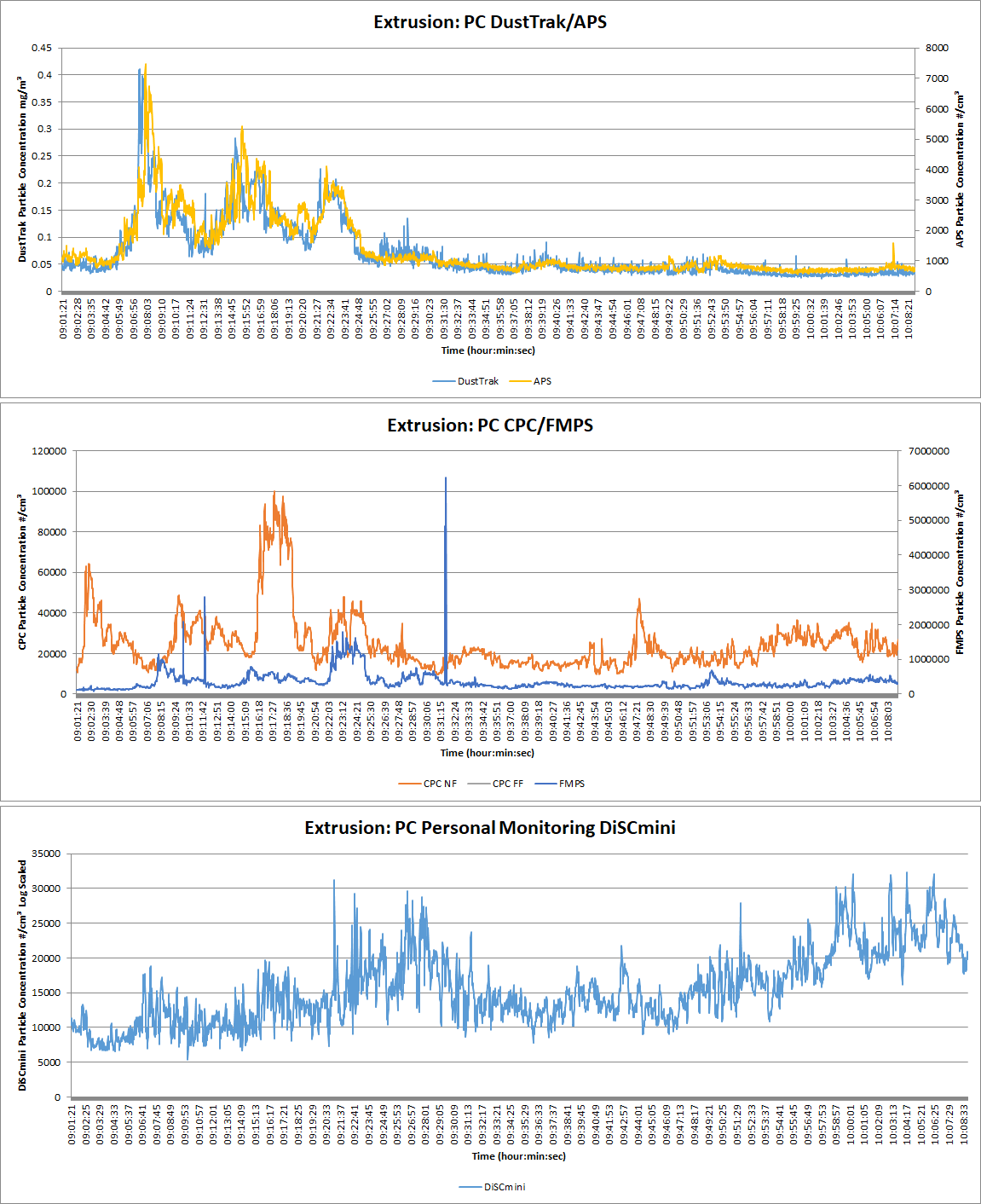


Figure S4g Time stamped particle detection during extrusion – PC only. Measurements performed by DustTrak, APS, FMPS, CPC and DISCmini, conducted during extrusion; includes extrusion of PC only.


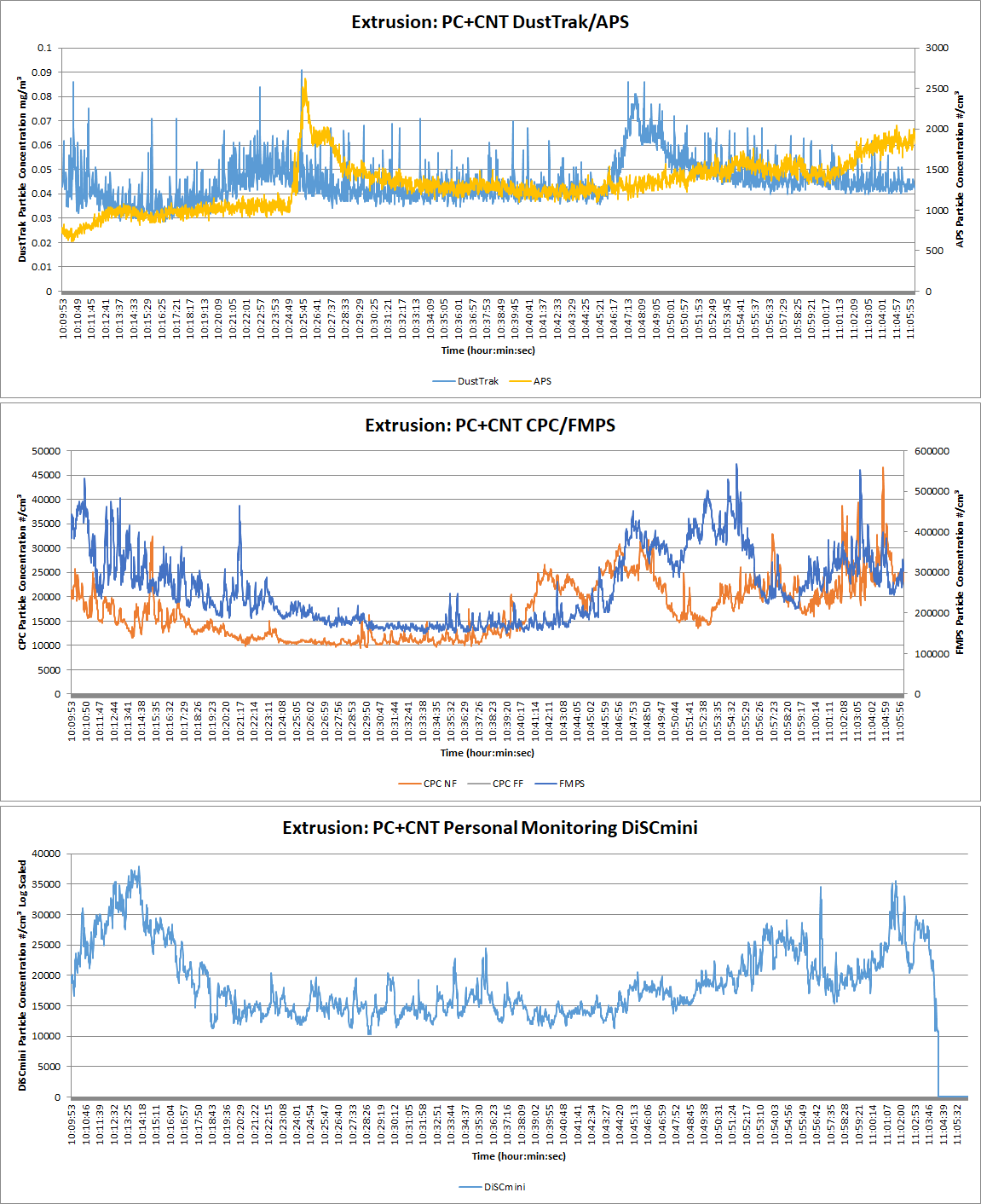


Figure S4h Time stamped particle detection during extrusion – PC with SWCNTs. Measurements performed by DustTrak, APS, FMPS, CPC and DISCmini, conducted during extrusion; includes extrusion PC with SWCNTs.

# Filter collection, analysed by SEM and EDX

Table 3 Sample collection details, during stages of PC/SWCNT filament production.

| Sample ID | Sample Description | Sample time (min) | Average flow rate (L/min) |
| --- | --- | --- | --- |
| 01 | Extrusion, NF Sample (extruder) | 141 | 2.018 |
| 02 | Extrusion, NF Sample (hopper) | 141 | 2.155 |
| 03 | Extrusion, Personal Sample | 147 | 1.575 |
| 04 | Filament production, NF Sample* | 154 | 1.777 |
| 05 | Filament production, FF Sample* | 157 | 2.204 |
| 06 | Filament production, Personal Sample* | 154 | 2.175 |
| 07 | Filament production, Residue collected from SWCNT-polycarbonate pellets | - | - |
| 08 | SWCNT Reference Sample (Tuball matrix 822: SWCNT + polyol ester) | - | - |

*For the filament production samples, the sample times and flow rates also include case study 1. NF=Near Field; FF = Fair Field

Table 5 SEM/EDXS results

| Sample ID | SEM Images | EDX Spectra |
| --- | --- | --- |
| 01 | 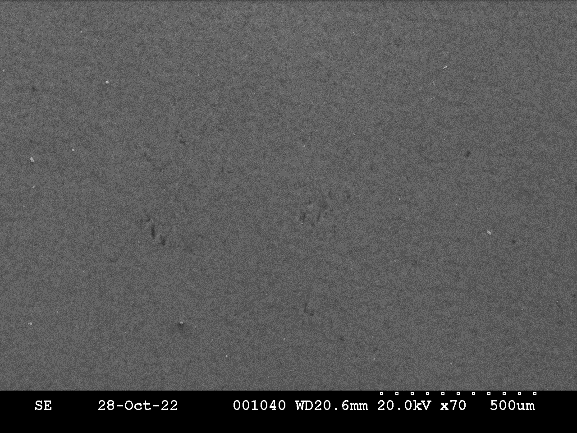 | NA |
| 01 - Filter had light loading. Sample showed typical inorganic and organic dust particles across filter. | | |
| 02 | 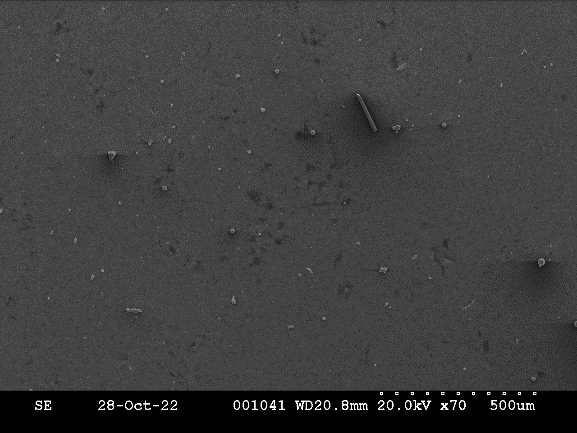 | NA |
|  | 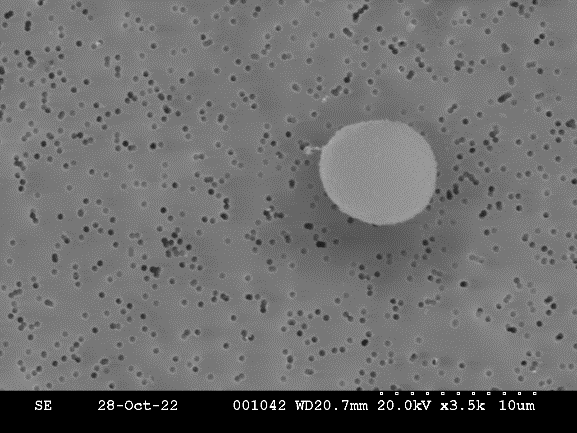  Width of particle approx. 7.32 µm | 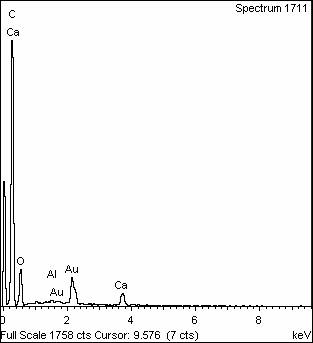 |
|  | 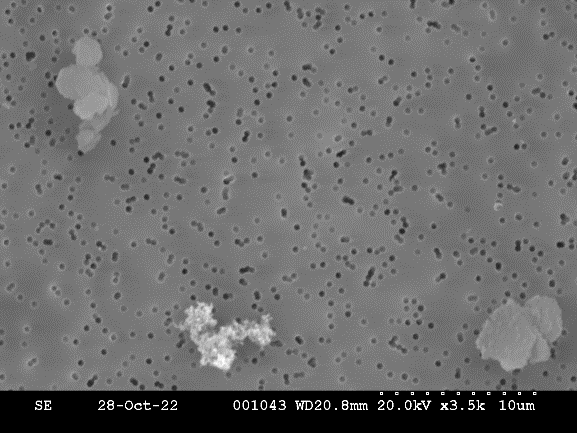  *  *  Width of agglomerates approx. 7.29 µm (left), 6.14 µm (middle, soot) and 5.84 µm (right) | *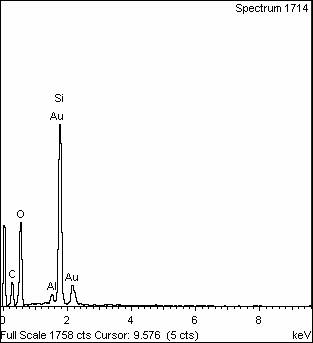  *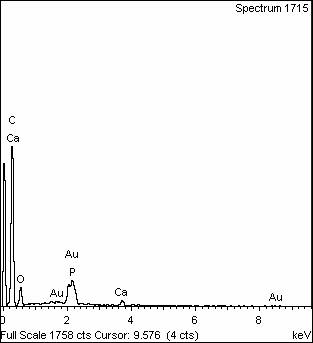 |
| 02 - Filter had light loading. Sample showed typical inorganic and organic dust particles across filter. Spherical particle in image 2 was observed across the filter. | | |
| 03 | 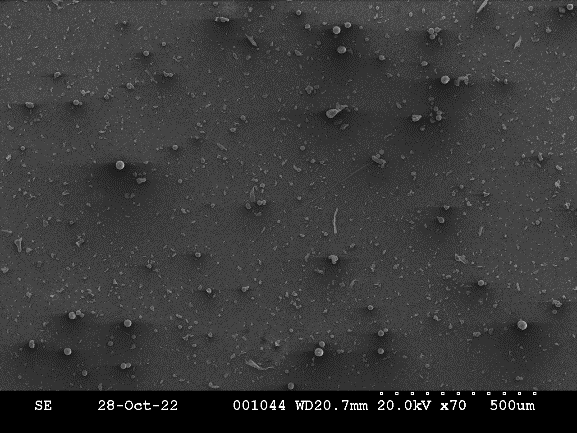 | NA |
|  | 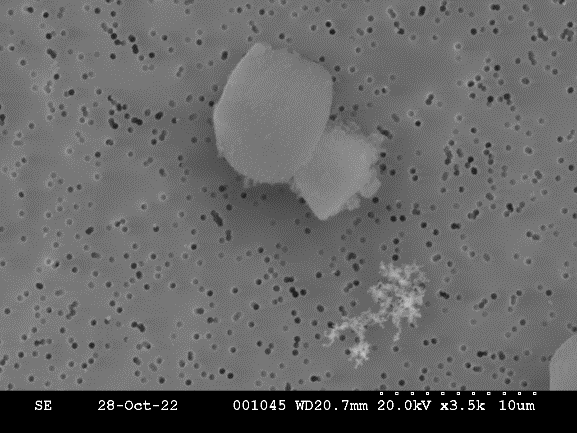  *  Width of particles/agglomerates approx. 8.38 µm (top left), 6.29 µm (top right) and 7.79 µm (bottom, soot) | *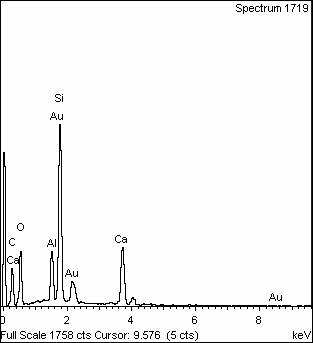 |
|  | 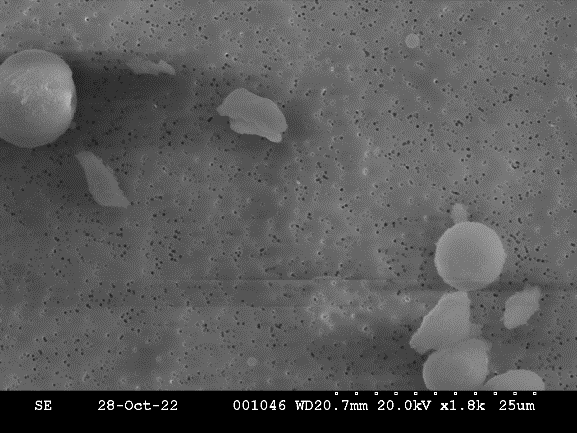  *  Particle widths approx. 1.76 – 12.48 µm | *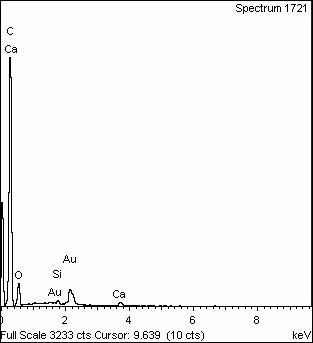 |
|  | 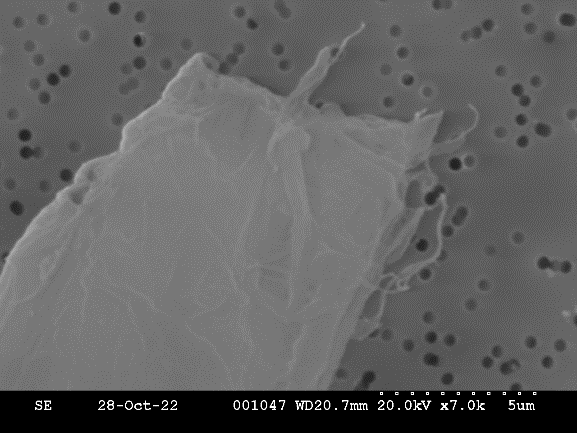  Width of fibrous strands at edges approx. 140 nm | 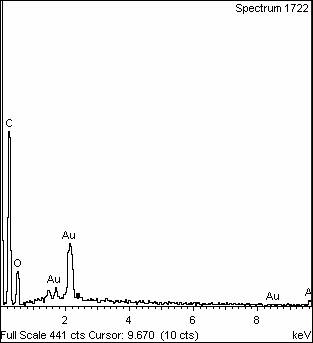 |
|  | 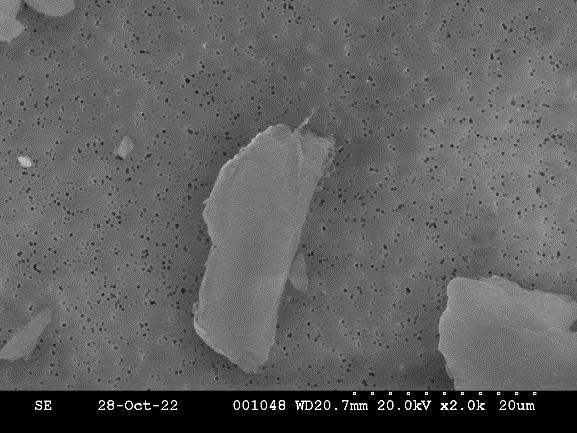  Dimensions of particle with fibrous strands approx. 26.92 x 10.53 µm | NA |
| 03 – Filter had moderate loading. Many different particles noted, mainly typical inorganic and organic dust particles. | | |
| 04 | 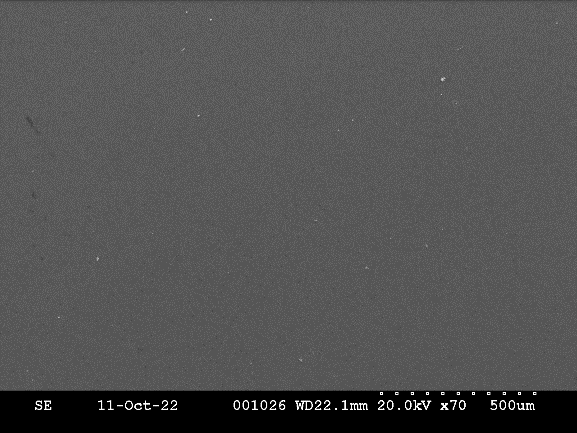 | NA |
|  | 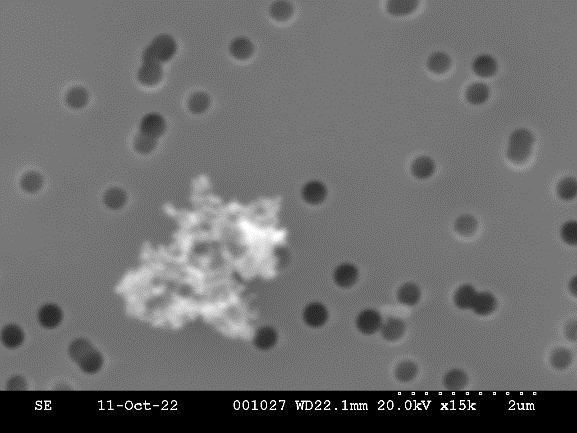  Width of bundle approx. 2.85 µm | 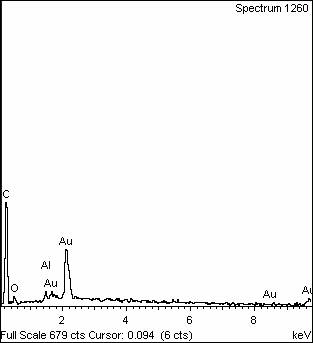 |
|  | 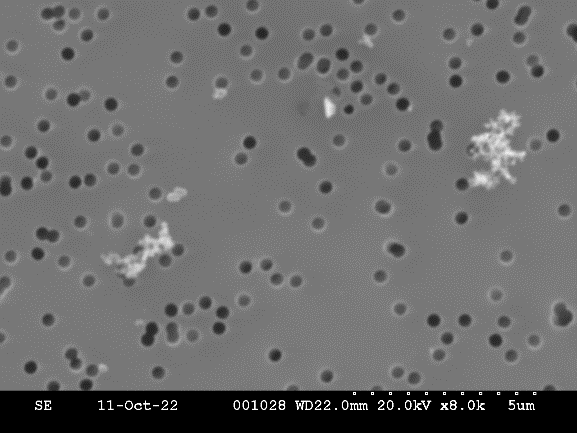  Width of bundles approx. 1.94 µm (left) and 2.25 µm (right) | NA |
| 04 - Filter had light loading. Sample showed typical inorganic and organic dust particles across filter. Image shows agglomerated carbon-based material with morphology of soot. | | |
| 05 | 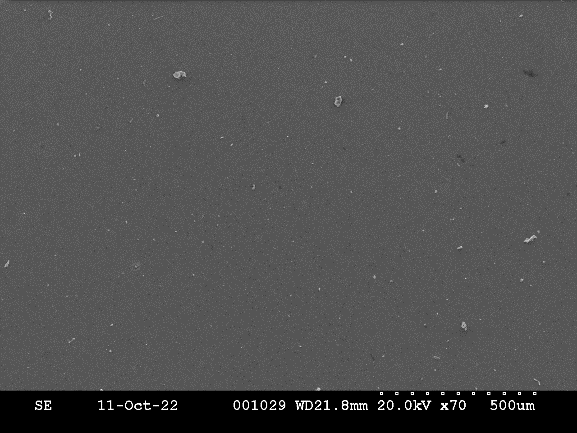 | NA |
|  | 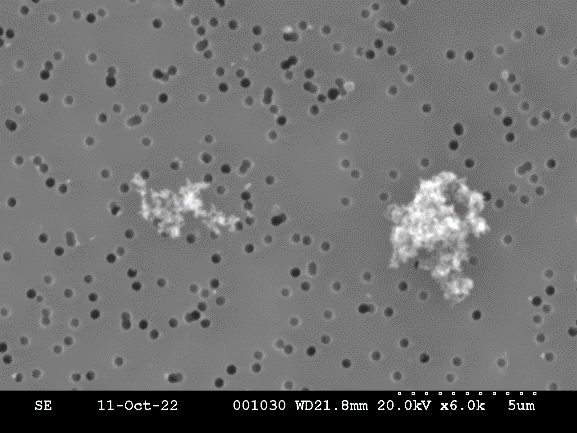  Width of bundles approx. 4.23 µm (left) and 4.90 µm (right) | 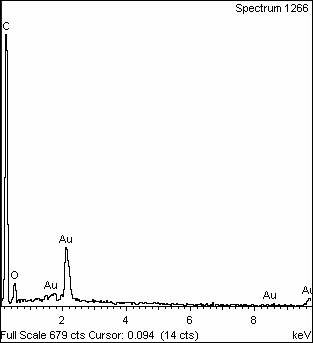 |
|  | 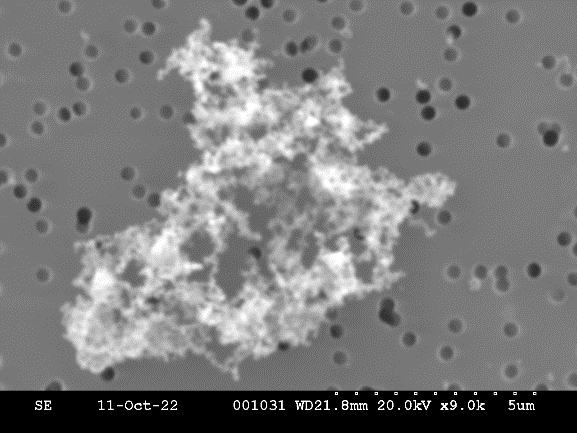  Width of bundle approx. 9.89 µm | 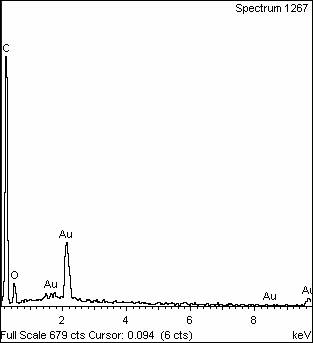 |
| 05 - Filter had slightly higher loading than NF (sample 04), with similar particle types observed across filter. Again image showing soot material. | | |
| 06 | 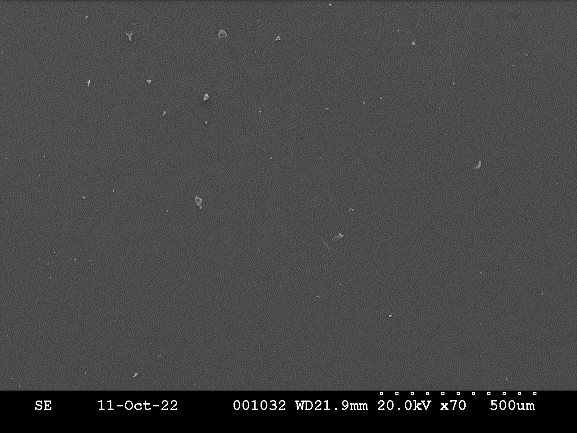 | NA |
|  | 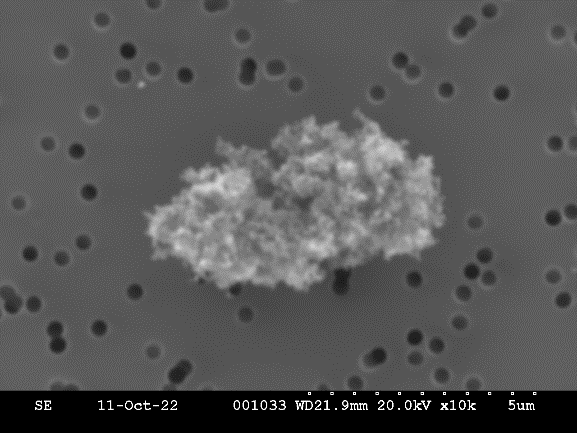  Width of bundle approx. 6.58 µm | 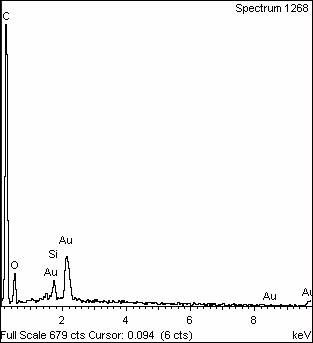 |
|  | 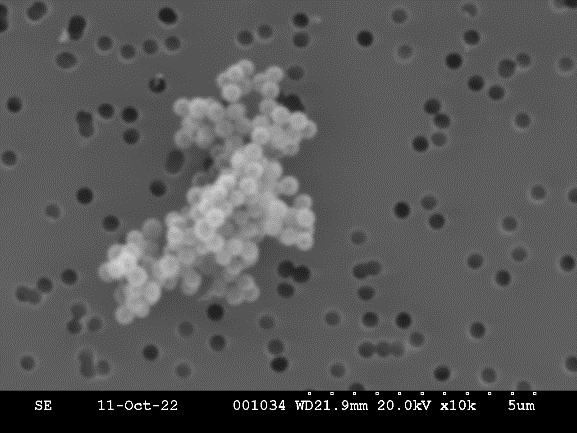  Width of bundle approx. 6.60 µm (individual particles approx. 400 nm) | 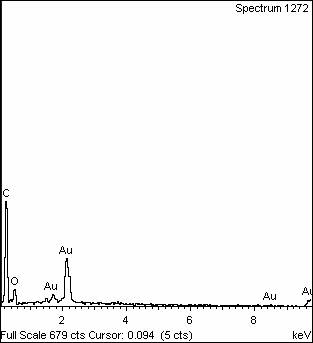 |
| 06 - Filter had similar loading to FF sample (sample 05). Again, typical inorganic and organic dust particles noted. Images show carbon-based materials observed with slightly different morphology. The image shows similar soot material to those shown for NF (sample 04) and FF samples (sample 05). | | |
| 07 | 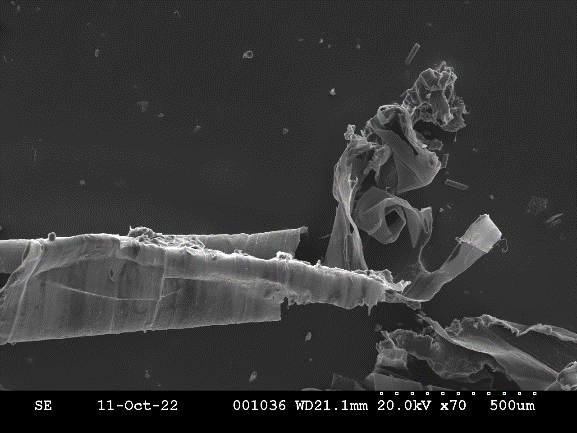 | NA |
|  | 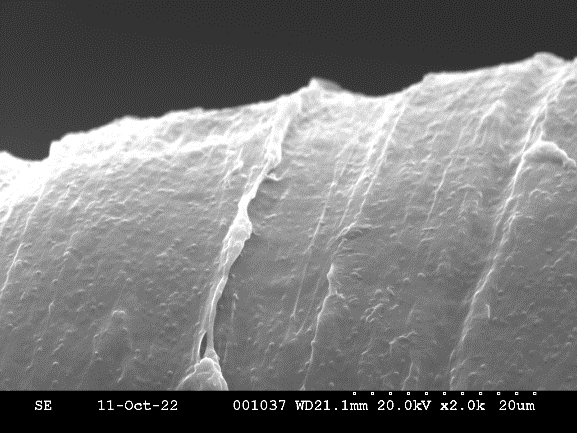 | NA |
|  | 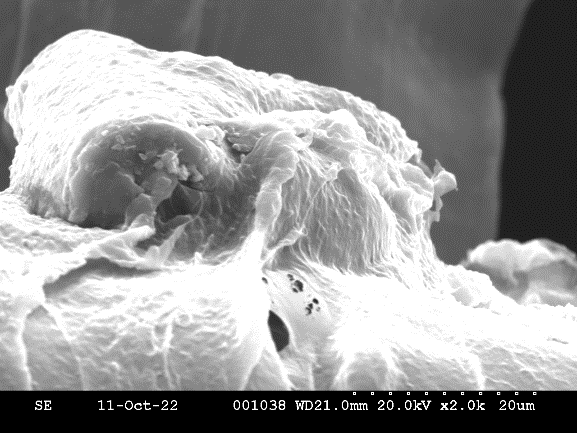 | NA |
|  | 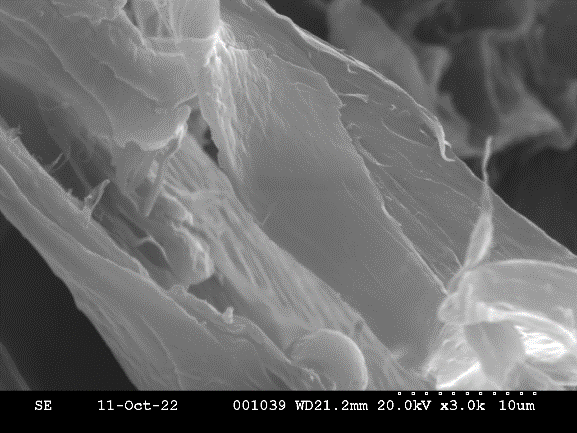 | NA |
| 07 - Surface of the residue from SWCNT-polycarbonate pellets shows some fibrous strands, however no individual SWCNT fibres can be observed. | | |
| 08 | 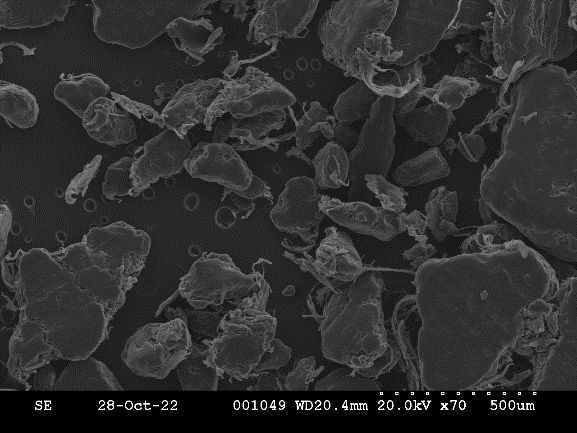 | NA |
|  | 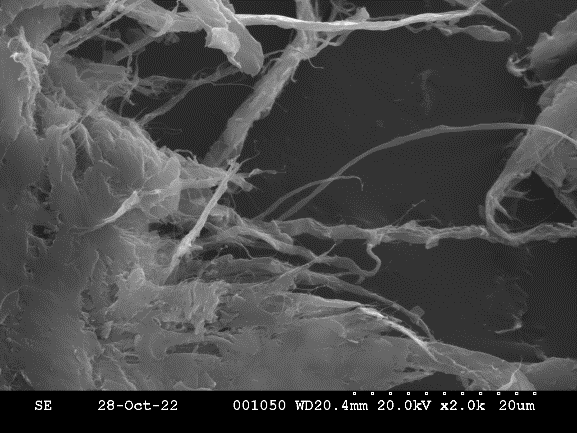 | 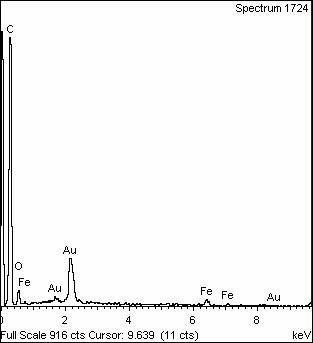 |
|  | 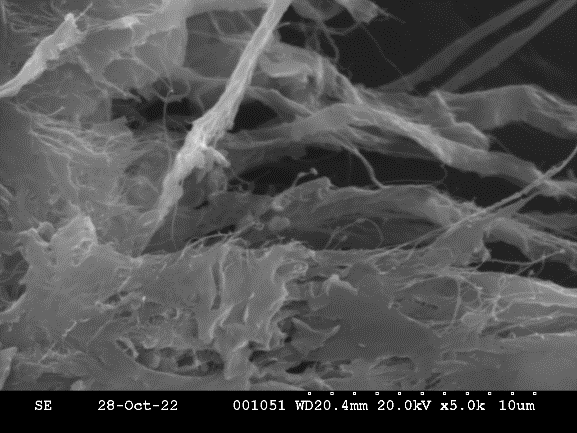  Widths of fibrous strands approx 125 – 158 nm | NA |
| 08 – Reference SWCNT material shows large agglomerates with many fibrous stands protruding from the edges. | | |
